# Harnessing the Anti-Inflammatory Properties of Polyphenols in the Treatment of Inflammatory Bowel Disease

**DOI:** 10.7150/ijbs.98107

**Published:** 2024-10-14

**Authors:** Diego Liviu Boaru, Oscar Fraile-Martinez, Diego De Leon-Oliva, Cielo Garcia-Montero, Patricia De Castro-Martinez, Alejandro Miranda-Gonzalez, Miguel A Saez, Leticia Muñon-Zamarron, Elisa Castillo-Ruiz, Silvestra Barrena-Blázquez, Rafael Cañonez-Zafra, Miguel Ángel Alvarez-Mon, Maria V Toledo-Lobo, Ana M Minaya-Bravo, Laura Lopez-Gonzalez, Raul Diaz-Pedrero, Jose V Saz, Agustin Albillos, Melchor Alvarez-Mon, Luis G Guijarro, Miguel A Ortega

**Affiliations:** 1 Department of Medicine and Medical Specialities, Faculty of Medicine and Health Sciences, Network Biomedical Research Center for Liver and Digestive Diseases (CIBEREHD), University of Alcalá, Alcalá de Henares, Spain.; 2 Ramón y Cajal Institute of Sanitary Research (IRYCIS), Madrid, Spain.; 3 Department of Surgery, Medical and Social Sciences, Faculty of Medicine and Health Sciences, University of Alcalá, Alcalá de Henares, Spain.; 4 Department of Biomedicine and Biotechnology, University of Alcalá, 28801 Alcalá de Henares, Spain.; 5 Department of General and Digestive Surgery, General and Digestive Surgery, Príncipe de Asturias University Hospital, Alcalá de Henares, Spain.; 6 Gastroenterology and Hepatology Departament. Ramón y Cajal University Hospital, Madrid, Spain; Ramón y Cajal Institute for Health Research (IRYCIS), Madrid, Spain; Network Research Center for Liver and Digestive Diseases (CIBEREHD), Madrid, Spain; University of Alcalá, Madrid, Spain.; 7 Immune System Diseases-Rheumatology, Oncology Departament and Internal Medicine (CIBEREHD), Príncipe de Asturias University Hospital, Alcalá de Henares, Spain.; 8 Unit of Biochemistry and Molecular Biology, Department of System Biology (CIBEREHD), University of Alcalá, Alcalá de Henares, Spain.

**Keywords:** Chron's disease, ulcerative colitis, inflammatory bowel disease, polyphenols, adjunctive therapy, immunomodulatory agents, antioxidants, gut microbiota

## Abstract

Inflammatory bowel disease (IBD) encompasses a spectrum of chronic inflammatory conditions affecting the gastrointestinal tract, notably ulcerative colitis (UC) and Crohn's disease (CD). Both UC and CD result from the interplay between genetic and environmental factors that trigger an exacerbated immune response against gut microorganisms, leading to non-resolving inflammatory damage in the mucosa of specific zones in the intestine. Despite extensive research, current treatments often entail invasive interventions with considerable adverse effects on patient well-being. Consequently, there is a pressing need to find alternative and complementary therapeutic strategies aimed at ameliorating chronic inflammation and restoring intestinal barrier integrity. Polyphenols are plant-based compounds formed naturally or as semi-synthetic/synthetic derivatives with proven health-promoting effects and translational applications in a broad spectrum of chronic diseases. Preclinical models of IBD largely support the efficacy of a broad variety of polyphenols due to their well-documented antioxidant and modulatory properties on the immune system and gut microbiota. Likewise, a growing number of studies using distinct types of polyphenols are being conducted in humans, although more efforts are still warranted. In the present review, the main polyphenols investigated *in vitro* and *in vivo* models of IBD will be summarized, as well as the available trials or observational data accessible in humans. Finally, the role of polyphenols in the clinical context of IBDs, along with the main problematics regarding their translational issues and concerns will be discussed, including bioavailability, their inclusion in healthy dietary patterns and foods, interaction with other drugs, and other important points to be addressed by future research.

## 1. Introduction

### 1.1. What are inflammatory bowel diseases?

Inflammatory bowel diseases (IBD) are defined as chronic intestinal inflammation resulting from the interplay between genetic and environmental variables that impact immune responses 1. The two primary categories of inflammatory bowel illnesses are ulcerative colitis (UC) and Crohn´s disease (CD) 2. Weight loss, diarrhea, stomach discomfort, and rectal bleeding are a few of the symptoms of them, but inflammation is the major characteristic shared by them 3. Men and women are equally affected by these illnesses, which can strike teenagers and adults. There are several distinctions in the symptoms of UC and CD, despite the similarities between the symptoms of these two illnesses 4.

**Figure 1:**
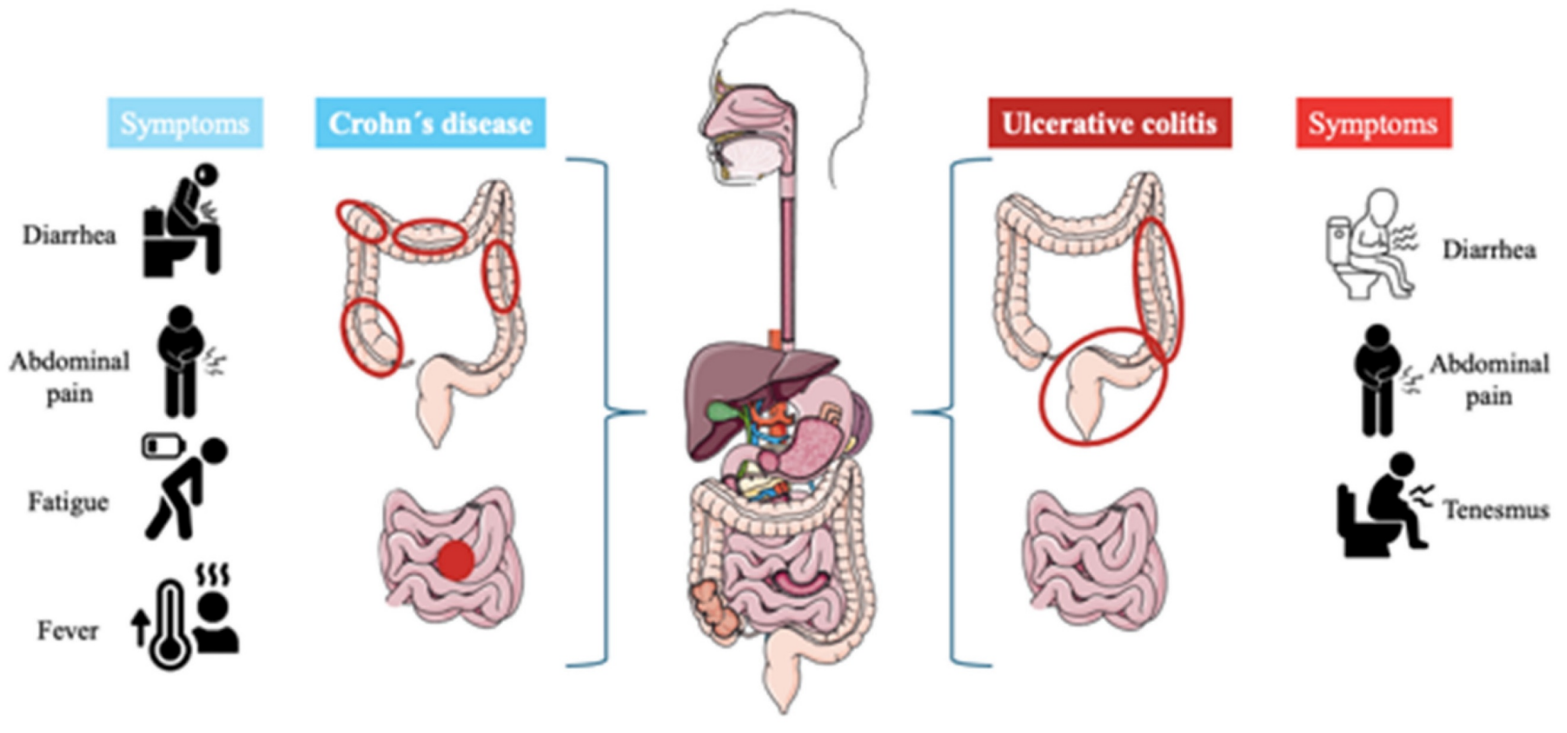
The left describes the progression of Crohn´s disease, the inflammation (red circles) can appear anywhere in the digestive tract and usually appears at the end of the small intestine, also it can occur in patches across the digestive tract. On the other hand, the right is described as ulcerative colitis, the inflammation affects the large intestine and rectum, and it can be extended to the entire colon or only part of its colon.

UC is a chronic disease characterized by generalized inflammation of the rectal and colonic mucosa 5. In 95% of the cases, UC primarily affects the rectum and may extend continuously and circumferentially to other parts of the large intestine. The clinical course usually includes periods of remission and flare-ups, which may occur spontaneously or in response to treatment 6. The incidence has increased in several regions of the world, especially in developing countries. Both genetic predisposition and environmental factors play a role in the etiology of the disease. Several studies have identified specific clinical and demographic features associated with distinct UC phenotypes and bad prognoses 7. On the other hand, Crohn's disease is a chronic inflammatory disorder affecting the gastrointestinal tract, characterized by lesions that can appear anywhere from the mouth to the anus and can lead to extraintestinal complications 8. The prevalence of Crohn´s disease is increasing in both adults and children. Genetic predispositions to this disease have been discovered, along with the identification of specific environmental factors related to its occurrence 9. Typical symptoms are diarrhea, abdominal pain, rectal bleeding, fever, weight loss, and fatigue 10. **Figure 1** describes the differences and similarities between UC and CD.

### 1.2. What are polyphenols and what are their mechanisms of action?

**Figure 2:**
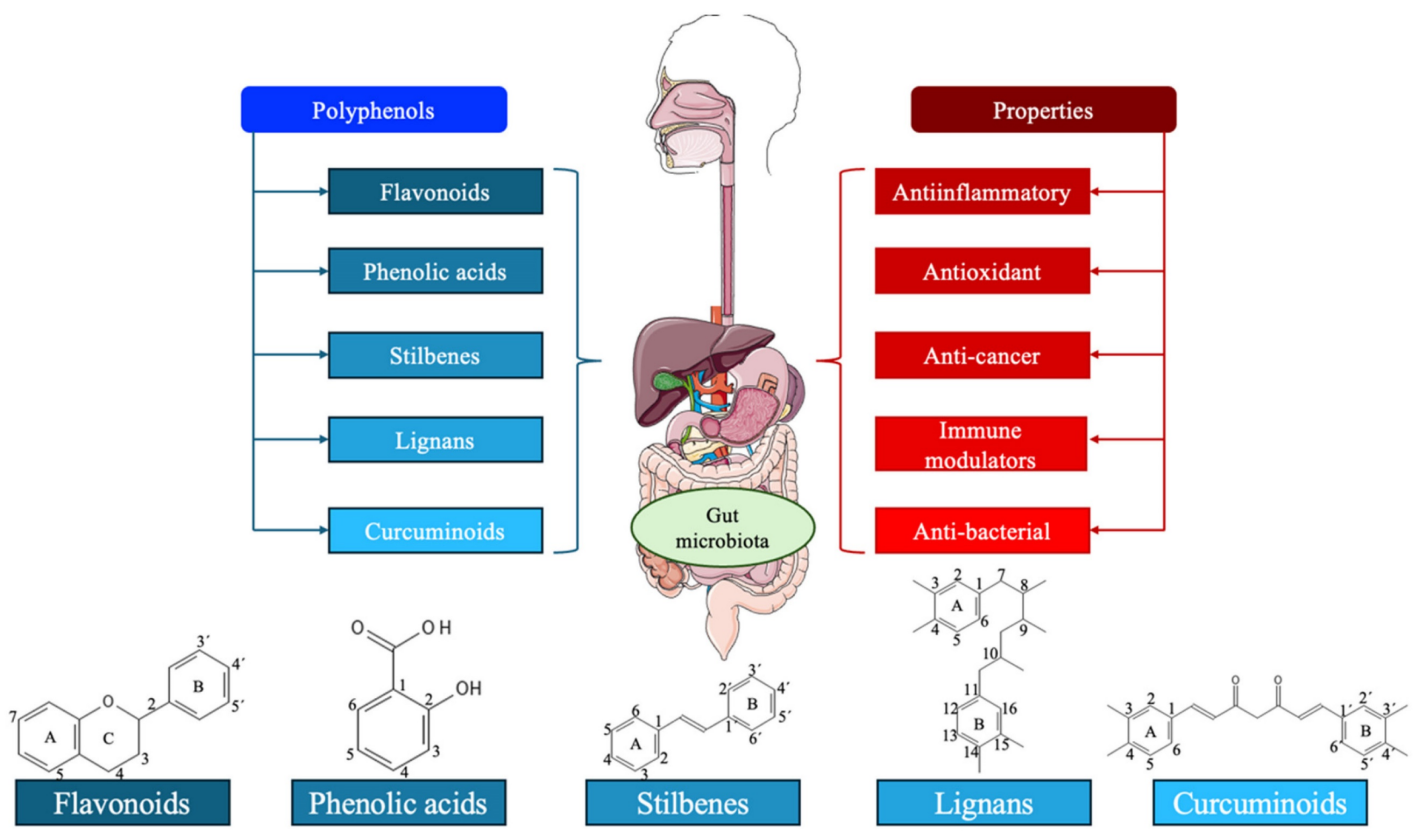
Polyphenols have a wide range of compounds. This narrative review describes the actions of five of them. First, it explains the different properties that have them, anti-inflammatory, antioxidant, ant-cancer, immune modulators, and anti-bacterial. Secondly, it describes the chemical structure of the five groups, which their derivates have in common, and will be described in the next point.

Polyphenols are a class of organic compounds that are predominantly found in fruits, green tea, vegetables, and whole grains 11. They can also be semi-synthetic or synthetic organic chemicals characterized by one or more hydroxyl moieties on one or more aromatic rings 12. With 8,000 different structural variations, they represent the largest group of secondary metabolites synthesized through shikimate/ phenyl propanoic or polyketide pathways in plants 13. Broadly, polyphenols can be classified into five groups: flavonoids, phenolic acids, stilbenes, lignans, and curcuminoids, or they can be divided into different subclasses attending to their number of phenol units, their molecular structures, the linkage types between phenol units, and the substituent groups. Thanks to their aromatic rings, double bonds, and numerous functional groups, polyphenols have effective antioxidant, anti-inflammatory, immune-modulatory, and anti-cancer properties 14-17. **Figure 2** summarizes the main types and properties of polyphenols.

One of the primary challenges faced by humans pertains to the presence of reactive oxygen species (ROS), which play intricate roles in various biological functions. These functions include combating pathogens, regulating blood pressure, and mediating cellular signaling processes 18. Their production can be modulated by physiological processes, or it can be introduced through the exogenous via 19. In normal conditions, there are more antioxidants than free radicals, but at the moment when the accumulation of ROS is higher than the antioxidants in cells or tissues, this process is called oxidative stress. It is caused because ROS has unpaired electrons, providing a higher chemical reactivity, and making it act as a potentially toxic molecule to induce an amount of degenerative disease by damaging the biomolecules 20. One of the key actions of polyphenols is their antioxidant capacity to scavenge ROS, including both free radicals and non-free radicals like hydrogen peroxide (H_2_O_2_), superoxide, and ions (O^-2^), hydroxyl radical (HO-), ozone (O_3_). They combat oxidative stress generated by lipids and nucleic acids by donating a single electron (SET) or through hydrogen atom transfer 21. As a consequence of this, they interrupt the initiation of radical reactions like the oxidation of proteins and sugar, peroxidation of lipids, and oxidative damage to nucleic acids 22. Also, they can chelate the ions of transition metals inhibiting the formation of free radicals in the Fenton and Haber-Weiss reaction 23. Another benefit, in this case, they act as co-antioxidants, which are involved in the regeneration of essential vitamins. Therefore, thanks to their antioxidant properties and their ability to propitiate ROS and free radicals, polyphenols are useful in improving human health, also aiding in the prevention of multiple diseases, and reducing the aging process 24.

On the other hand and as has been described before, polyphenols also exert a pivotal anti-inflammatory and immunomodulatory activity. In the last years, *in vivo* and *in vitro* models have shown that dietary phenolic compounds are able to modulate the NLPR3 pathway 25, having a protective activity on inflammation. NLRP3 is an important node that links the signaling pathways between inflammation and the redox response, thus influencing cellular responses against ROS 18. Also, it is suggested that they have a radical scavenging activity, NADPH oxidases (NOX) inhibition, the regulation of enzymes involved in arachidonic acid metabolism, arginine metabolism, MAPK pathway, and the inhibition of pro-inflammatory enzymes such as cyclooxygenase (COX) 2, inducible nitric oxide synthase (iNOS), lipoxygenase (LOX), inhibition of nuclear factor kappa B (NF-κB), and the activation of activator protein-1 (AP-1) DNA binding 26. On the other hand, some studies suggest that polyphenols have effects on the expression of various inflammatory mediators, including interleukin-1beta, (IL-1β), interleukin-6, (IL-6), and tumor necrotic factor alpha (TNF-α) 27.

Polyphenols can also exert direct effects on the gut microbiota —a diverse community of bacteria, fungi, viruses, and other microorganisms that play crucial roles in the host, including reinforcing intestinal integrity, regulating metabolism, defending against pathogens, and modulating the immune system 28. Although the precise mechanism remains incompletely understood, it is theorized that polyphenol metabolites may stimulate beneficial gut bacteria 29. Increasing research suggests that the presence of phenolic compounds may enhance the beneficial actions of probiotics 30. In parallel, polyphenols show antibacterial activity against a large number of bacteria (including Gram-positive and Gram-negative bacteria) and fungi 31, thus explaining their regulatory role on gut microbiota. However, more research is needed to delineate how polyphenols and related metabolites, either phase II metabolites or those generated by the gut microbiota, might interact with systemic tissues, using *in vitro* and *in vivo* models 32.

Finally, polyphenols are also being investigated for their antitumoral activities. Indeed, a broad spectrum of studies supports the multiple anticarcinogenic properties of plant-derived polyphenols, including their inhibitory effects on the proliferation of cancer cells, tumor expansion, angiogenesis, inflammation, and metastasis whereas some studies show potential synergistic effects when polyphenol treatment combined with chemotherapeutic agents 33.

### 1.3. Polyphenols and Inflammatory Bowel Diseases. Where is the potential?

As mentioned above, IBD is a complex and multifactorial disease triggered by the interaction between genetic and environmental factors. The interaction between genetic and environmental factors triggers an impaired immune response against gut microorganisms in IBDs leading to non-resolving inflammatory damage in the mucosa of specific zones in the intestine 34. Regarding the genetic component, at least 240 gene loci related to inflammatory responses (mainly in the nucleotide oligomerization domain -NOD- receptors, chemokines, cytokines), autophagy, and antimicrobial peptides seem to be associated with the predisposition and occurrence of IBD 35. Environmental factors associated with IBD pathogenesis include stress, smoking, unhealthy lifestyle, and poor hygiene, whereas the use of non-steroid anti-inflammatory drugs, antibiotics, or appendectomy has also been associated with IBDs 36.

An altered microbiota (gut dysbiosis) is a central mechanism implicated in the pathogenesis of IBD. A set of bacteria seems to be associated with IBD development, including *Mycobacterium paratuberculosis*, adherent-invasive *E. coli* (AIEC), *Helicobacter pylori* and non-pylori species, *Campylobacter concisus*, *Enterococcus faecium*, enterotoxigenic *Bacteroides fragilis* (ETBF), *Fusobacterium varium* and *Ruminococcus gnavus*, whereas alterations in the mycome and virome have also been observed influencing immune responses 37,38. Conversely, some microorganisms are inversely related to IBDs such as *Faecalibacterium prausnitzii*, Roseburia species, particularly *Roseburia hominis* and *Roseburia intestinalis*, Ruminococcaceae, including *Clostridium leptum* and *Clostridium sporogenes*, *E. coli*, *Bacteroides fragilis*, and *Akkermansia muciniphila*. These microorganisms are responsible for producing favorable microbial metabolites such as short-chain fatty acids (SCFAs), tryptophan derivatives, and secondary bile acids, among other products, playing crucial roles in regulating immunity, reducing inflammation, and maintaining gut homeostasis 37. Collectively, the phenomena of gut dysbiosis are directly involved in the exacerbated inflammatory responses related to IBD via direct interactions with the immune system or through the production and release of toxins/microbial metabolites with potential immunomodulatory effects 39.

Regarding the immunological changes occurring in IBDs, a broad spectrum of changes affecting both the innate and adaptative immune systems have been reported. The aberrant innate immunity occurring in the gut of patients affected by IBD encompasses immune and non-immune cells, involved in the sensing and response to the gut microorganisms. These cells include 1) Paneth cells, tuft cells, and other epithelial cells (implicated in the secretion of antimicrobial peptides that contribute to limiting bacterial growth and invasion); 2) globet cells (responsible for producing mucine, serving as prevents the entry and invasion of microorganisms in the different gut layers); and 3) gut epithelial cells (enterocytes) and stromal cells, responsible for detecting invading bacteria through extracellular and intracellular pattern recognition receptors (Toll-like receptors — TLRs and NOD-like receptors-NLRs). Innate immune cells include macrophages, granulocytes, innate lymphoid cells (ILCs), and dendritic cells (DCs), involved in the rapid initiation of inflammatory responses mediated by the secretion of cytokines and chemokines and recruitment of inflammatory adaptative cells 40,41. Adaptative immune responses associated with IBDs are mainly represented by B cells (implicated in humoral response and T helper cells, particularly Th1, Th2, Th17, and regulatory T cells (Tregs) 42. Within these cells, compelling evidence seems to defend that Th17 and Treg could have greater relevance in the development of IBDs; however, the dual role that these cells and their released products partly explain the difficulties in the available therapies directed against these and other inflammatory mediators 40,43. Immune dysfunction is accompanied by aberrant levels of a broad spectrum of cytokines tightly linked to IBD pathogenesis, including IL-1β, IL-18, IL-33, IL-6, IL-10, IL-17 (and their isoforms), TNF-α, tumor growth factor beta (TGF-β), along with chemokines IL-8, chemoattractant protein (MCP)-1, macrophage inflammatory protein (MIP)-1α and 1β, MCP-3, MIP-3α, CXCL5, CXCL8, CXCL10, and RANTES 40. Interestingly, inflammatory responses associated with UC are dominated by cytokines such as IL-4, IL-5, IL-9, and IL-13 secreted by Th2 cells, whereas in the case of CD, IL-1, IL-6, IL-8, TNF-α, and IFN-γ secreted by Th1 and Th17 cells are more abundant 44. Therefore, cytokine and immune response patterns can be important for understanding and discerning both entities.

Finally, other important processes beyond altered immunity and dysbiosis are playing a pivotal role in IBD development. For instance, oxidative/nitrosative stress is also a critical factor implicated in the initiation and progression of IBD. Overproduction of ROS and oxidative stress is triggered during inflammation because of the inflammatory responses that occur in the colonic tissue 45. All these mechanisms lead to significant changes in the functioning of the gut, from a molecular to a systemic level.

**Figure 3:**
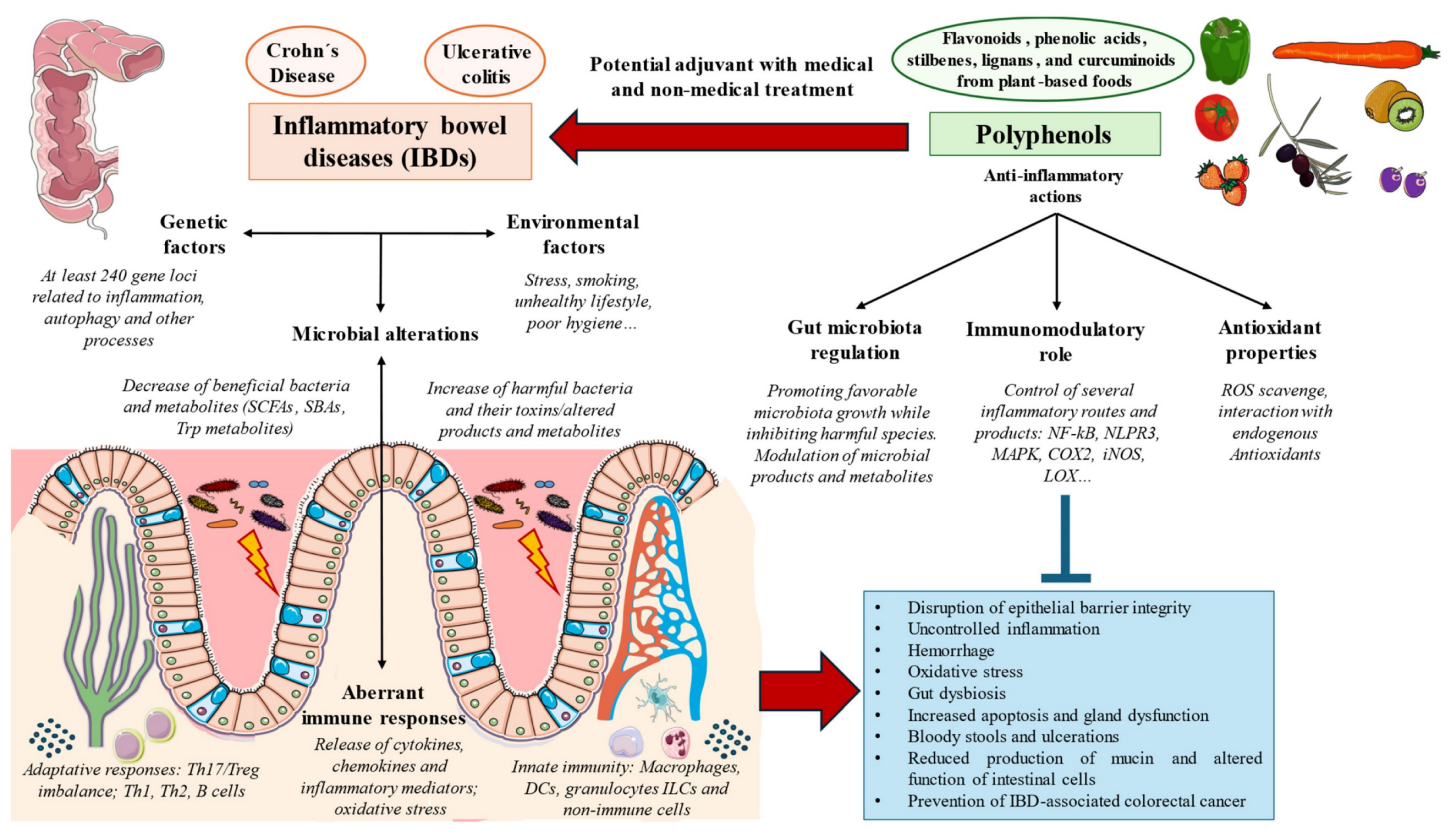
Pathogenesis of IBDs and the mechanism of action of polyphenols in this condition.

Polyphenols belong to a group of natural compounds contained in foods and plant sources known as nutraceuticals with proven benefits either in health promotion or disease prevention 46. In the event of IBDs, the relevance of polyphenols has been described in previous literature, exerting their benefits in this condition by many different mechanisms including the reduction of epithelial damage, inflammation, hemorrhage, oxidative stress, gut dysbiosis, apoptosis, gland dysfunction, bloody stools, and ulcerations while promoting an increase in mucin content, number of crypts and reinforcing the integrity of the epithelial barrier 47. Because of this, a growing number of studies suggested that these actions make polyphenols a potential therapeutic approach as an available adjuvant to medical and non-medical treatment to aid in the clinical management of IBD, although the available evidence to support their clinical use is still limited 48. Besides, previous works have demonstrated that patients with IBDs show an increased risk of developing colorectal cancer (CRC) 49, making the use of polyphenols a promising strategy to prevent and also aid in the management of this concern 50,51. **Figure 3** summarizes the pathophysiological basis of IBDs and the main actions of polyphenols.

In the present narrative review, a search for the principal polyphenols currently investigated in the context of IBDs (including flavonoids, phenolic acids, stilbenes, lignans, curcuminoids, and other polyphenols from plant-based sources) will be conducted using the bibliographic databases PubMed, Scopus, and ScienceDirect. For each polyphenol explored, the search terms will be ("inflammatory bowel disease" OR "Crohn's disease" OR "ulcerative colitis"). Subsequently, the available studies about these terms will be evaluated, highlighting the main findings/conclusions obtained and discerning their origin (preclinical or clinical). As most studies have been conducted *in vitro* or *in vivo*, we will highlight those polyphenols explored in humans with a specific subsection, differentiating into observational or intervention studies. Finally, a critical perspective will be provided on the main limitations and issues surrounding the use of polyphenols in general and in the context of IBDs in particular, based on the most updated scientific evidence.

## 2. Polyphenols in inflammatory bowel disease

In this section, the use of the main types of polyphenols (flavonoids, phenolic acids, stilbenes, lignans, curcuminoids, and others) in IBDs will be summarized. As aforementioned, most studies have been conducted *in vitro or in vivo*. *In vitro* models to study IBDs commonly include Caco-2, HT29, or RAW264.7 cells, as well as organoids 52. Animal models of IBD are commonly mice or rats. IBD can be induced chemically by agents like dextran sulfate sodium (DSS), 2,4,6-trinitrobenzene sulfonic acid (TNBS), acetic acid (AA), and oxalazone, genetically (knock-out models), or being induced by specific bacteria 53,54. Thus, *in vitro* and animal models used in the different studies will also be remarked.

### 2.1. Flavonoids

Plants produce their synthesis and are extensively distributed in the tissue plant as a glycoside form. The flavonoid structure generally comprises aglycones (the non-sugar fragment part of the appropriate glycoside) or glycosides. They all have a basic structure made of diphenyl propane (C6-C3-C6), their phenolic rings (ring A and ring B) are connected by a heterocyclic ring, and their ring C is usually a closed pyran 55.

The diversity of the structures of the flavonoid molecules rises from different modifications in the oxidation situation of the central pyran ring, and the hydroxylation sequence. As a result of these combinations, there are described a wide range of compounds such as flavones, flavonols, flavanones, isoflavones, flavanols, and anthocyanidins/anthocyanins. These modifications are determined by whether a double bond exists between C2 and C3, and the formation of a carbonyl group by C4 56.

Besides, flavonoids are not considered a stated nutrient yet due to their physiological functions in plants, they are important in the human diet as healthy ingredients. According to epidemiological studies, diets based on an abundance of flavonoids are relational with an increase in longevity and the reduction of cardiovascular disease incidence, and cancer risk 57. Their biological function is determined by their bioavailability, their anti-inflammatory, and antioxidant properties, and other activities such as vasodilatory, anticancer effects, and anti-ischemia 58.

#### 2.1.1. Flavones

This subgroup is an important part of flavonoids. Their skeleton comprises 2-phenyl-γ-pyrone, which is involved in the heterocyclic pyrone ring connected with two phenyl rings 59. Another characteristic of the flavones is the position of the O-glycoside joint, it appears in the C3 and C7 positions. This linkage units the sugar group with the aglycone 60. Their C-glycosides joints do have not much research about their function. In nature, they are found in herbs like parsley and celery and grains such as oats, rye, barley, and sorghum.

As part of the flavone subgroup, it is formed by many components, here it is described the action of apigenin, baicalein, baicalin, wogonoside, wogonin, luteolin, tangeretin, galangin, nobiletin, and chrysin.

##### A) Apigenin

**Apigenin** is a natural compound present in parsley, chamomile, celery, vine spinach, artichokes, and oregano 61. It suggests having anti-inflammatory and antioxidant properties that are beneficial in IBD therapy. Also, it can inhibit the transactivation promoted by tumor necrosis factor (TNF)-α 62. There is one study that shows the efficacy of apigenin in the murine DSS colitis model through blocking the inflammasome vias by the production of IL-1β and downregulation of iNOS and COX-2 and decreasing serum levels of matrix metalloproteinase-3 63. Apigenin can also significantly relieve the intestinal pathological injury in these animal models, increasing goblet cell quantity and mucin secretion, promoting anti-inflammatory cytokines IL-10 expression, and inhibiting the expression of proinflammatory cytokines, TNF-α, IL-1β, IL-6, and MPO activity of colon tissue 64. It is shown that in combination with epigallocatechin-3-gallate (EGCG) in the Flavo-Natin has protective functions in the oxazolone-induced colitis model, another of the benefits is the maintenance of the intestinal epithelial barrier, shaping microbiota, and their proinflammatory and antioxidant properties 65. Also, apigenin is found to inhibit the inflammation promoted by carcinogenesis in general, by repressing the signal transducer and activator of transcription (STAT)-3- NF-κB signaling 66. Apigenin lead to increased zonulin 1 (ZO-1), claudin-1 and occludin expressions to restore the integrity of the intestinal barrier, regulating the microbial populations of Akkermansia, Turicibacter, Klebsiella, Romboutsia, etc., and its metabolites (SCFAs), thus attenuating DSS-induced colon injury 64. Apigenin can also inhibit both canonical and non-canonical NLRP3 inflammasome pathways by decreasing proinflammatory IL-1β and IL-18 cytokine levels and regulating cleaved caspase-1 and caspase-11 enzymes 67.

##### B) Baicalein and baicalin

**Baicalein** and its aglycone baicalein possess multi-fold therapeutic properties and are mainly found in the roots of *Oroxylum indicum* (L.) Kurz and *Scutellaria baicalensis* Georgiis (SBG) 68. Both baicalein and baicalin have shown significant and potential benefits for IBDs. *In vitro* and *in vivo* models have shown that **baicalein** reduces the impact of IBD by inhibiting the COX-2 activity and decreases the phosphorylation of Ikappa kinase (IKK)-β degrading the Ikappa-beta-alfa (IκΒα) 69, also ameliorating UC by improving intestinal epithelial barrier via AhR/IL-22 pathway in ILC3s 70 and through the inhibition of TLR4/MyD88 signaling cascade as well as inactivation of NLRP3 inflammasome 71. Moreover, the modulatory effects of baicalein in Ahr receptors can restore the balance of Th17/Treg cells and diminish proinflammatory cytokines such as IL-17, IL-6, and TNF-α while increasing anti-inflammatory cytokines such as IL-10 and TGF-β; and epithelial protective cytokine IL-22 in UC mice 72. Also, baicalein is involved in the reduction of the phosphorylation of p65 and the nuclear translocation and downregulation of the DNA-binding activity of the NF-κB in the STAT3 and cyclin D1 expression 73. It seems that engineered formulas like baicalein-decorated zinc phosphates could exert greater improvements in animal models of colitis when compared to baicalein alone 74. In parallel, baicalein can also make synergy with other compounds such as betaine, alleviating colonic inflammation and preventing associated tumorigenesis 75.

**Baicalin** exerts similar actions to baicalein on autoimmune diseases by regulating cell proliferation and STAT gene expression 76. Liang *et al.* 77 explored *in vivo* the use alone or in combination of baicalin and baicalein from using Scutellaria baicalensis herb (SB). They observed that Baicalin and baicalein had significantly different effects on UC, as well as when both compounds were combined. They show that the combination of the two drugs provides a more comprehensive treatment; and also that compared with baicalein, baicalin was more potent for the treatment of large intestine disease. Baicalin has also been shown to act as an anti-inflammatory, anticarcinogenic antioxidant, and immunomodulatory drug, also aiding in the maintenance of intestinal barrier and flora balance 78. The studies highlight its effects in the regulation of Th17/Treg populations 79, promoting the proliferation of CD4(+)CD29(+) cells and modulating immunosuppressive pathways 80, enhancing the polarization of the anti-inflammatory phenotype in macrophages (M2) 81. Also, it is implicated in the down-regulation of the expression of MIF, the number of macrophages, and the amount of macrophage-related cytokines, including MCRP1 MIP-3α 82, inhibition of IL-33 expression and subsequent NF-κB activation 83, blockage of the TLR4/NF-κB-p65/IL-6 signaling pathway 84, regulation of the autophagic flux 85, modulation of gut microbiota and SCFAs 86, amelioration of inflammation, oxidative stress and apoptosis in intestinal cells 87,88, and control of sphingolipid metabolism and sphingolipid signaling pathway 89. The combination of baicalin with berberin or emodin has also demonstrated significant synergic benefits in the treatment of animal models of UC 90,91.

Two studies conducted by Yu *et al.* 80,92 assessed the efficacy and effects of baicalin in human cells from patients with UC (N=33 divided into active and inactive groups) and compare it with irritable bowel syndrome and healthy subjects. In these studies, peripheral mononuclear immune cells were extracted from these groups and cultured them *in vitro*. Baicalin was added at different concentrations (5, 10, 20, or 40 µmol). In one study, they observed that the percentages of CD4+CD29+ T cells were lower with 40 and 20 μmol/L baicalin treatments compared to the no baicalin treatment, driving a significant increase in the expression of IL-4, TGF-β1, and IL-10, and the p-STAT6/STAT6 ratio. In parallel, these treatments decreased the expression of IFN-γ, IL-5, IL-6, RORC, Foxp3, and T-bet, as well as the ratios of T-bet/GATA-3, p-STAT4/STAT4, and p-NF-κB/NF-κB 80. Likewise, 40 µmol baicalin significantly decreased IL23R gene expression in UC patients, whereas the 20 µmol and 40 µmol baicalin treatments significantly decreased p-STAT4/STAT4 ratios IFN-γ and IL-4 and increased p-STAT6/STAT6 ratios and IL-10 levels 92.

##### C) Luteolin

**Luteolin** is a natural compound found in carrots, parsley, broccoli, peppers, celery, olive oil, onion leaves, cabbages, apple skins, chrysanthemum flowers, peppermint, thyme, rosemary, and oregano 93. Its expression is involved in inhibiting pro-inflammatory mediators such as COX-2, TNF-α, and interleukin (IL)-6, and appears in regulating multiple vias like NF-κB 94. Recent studies suggest that this natural recurse decreases inflammation in rats with ulcerative colitis by regulating the gut microbiota 94. In the cell line HT-29 colon epithelial cells, luteolin negatively affects the regulation of inflammatory signaling cascades due to their anti-inflammatory action, inhibiting the JAK/STAT pathway 95. Likewise, luteolin seems to inhibit TNF-α-induced IL-8 production in this cell line through blockade in the phosphorylation of MAPKs, following IkappaB degradation and NF-kappaB activation 96 Also, in RAW264.7 cells, luteolin acts as an antagonist of the IKKα/β by blocking its phosphorylation and the action of NF-κB 97. Regarding DSS-induced UC in rats, luteolin seems to reduce colonic inflammation and intestinal barrier damage through the modulation of various pathways including the suppression of the STAT3 signaling pathway by SHP-1 98 or restoring the balance between NCR-ILC3/NCR+ILC3 99. Likewise, the administration of luteolin seems to drive favorable changes in the gut microbiota, enhancing the levels of lactobacillus, Bacteroides, Roseburia, and Butyricicoccus while reducing DSS-induced enhanced ratios of Lactobacillus and Prevotella_9 100. Intraperitoneal administration of luteolin was also shown to improve the relative abundance of anti-inflammatory microorganisms (i.e. Clostridia UCG-014, Enterorhabdus, Blautia and Lachnospiraceae NK4A136 group) while attenuating pro-inflammatory species (From the genera Turicibacter, Streptococcus, Staphylococcus, Clostridium sensu stricto 1, Romboutsia, Parasutterella, and Escherichia-Shigella) 101. Interestingly, luteolin strongly demonstrated utility in alleviating associated physical UC symptoms compared to apigenin or Xanthohumol administration. On the other hand, luteolin (20 and 50mg/kg) significantly attenuated the disease activity index (DAI), colon shortening, and histological damage while decreasing the expression of inflammatory mediators, such as iNOS, TNF-α, and IL-6 102. Luteolin can also stimulate total antioxidant defenses (promoting the activity of the superoxide dismutase (SOD) or the catalase (CAT) and alleviating oxidative stress, mainly through the Nrf2 signaling pathway and the decrease of malondialdehyde (MDA) 103. Finally, luteolin led to metabolomic changes in UC rats, leading to reductions in l-malic acid, creatinine, l-glutamine, and l-lactic acid levels accompanied by elevations in dimethyl sulfone, 5-methylcytosine, cysteine-S-sulfate, and jasmonic acid levels 103. Furthermore, differential metabolites primarily participated in d-glutamine and d-glutamate metabolism, glutathione metabolism, and citrate cycle pathways, thus demonstrating the multiple roles of this polyphenol.

##### D) Wogonoside and wogonin

Together with baicalein, baicalin, wogonoside, and wogonin are polyphenols representative of the flavone group, extracted from plants of the genus Sculletaria such as *SBG* 104. **Wogonoside** seems to alleviate colitis by protecting against intestinal barrier dysfunction through the reinforcement of tight junctions via regulation of the MLCK/pMLC2 signaling pathway in Caco2 cells 105, whereas in DSS-induced UC mice this compound seemed to lead to dual inhibition of NF-κB and NLRP3 inflammasome 106. **Wogonin** treatment effectively prevented colonic ulceration, neutrophil infiltration, oxidative stress, pro-inflammatory cytokines, and histological changes in DSS mice models. In more detail, Zhou *et al.* 107 showed that this compound promoted apoptosis by inhibiting Bcl-2 and enhancing the expressions of Bax, caspase-3, and caspase-9. Likewise, it led to a marked downregulation of COX-2 and iNOS, which led to the suppression of NF-κB. Moreover, wogonin also regulated the Nrf2 signaling pathway and decreased the activation of TLR-4/NF-κB. Recently, Ye *et al.* 108 showed that the effects of this polyphenol were also partly mediated by regulating the plasticity of ILC3/ILC1. They hypothesized that its specific mechanism is to bind to AhR directly or activate the AhR pathway indirectly by altering the tryptophan metabolisms of gut microbiota.

##### E) Other relevant flavones

Other less studied but relevant flavones investigated include tangeretin, nobiletin, and chrysin. **Tangeretin** is another flavone member and a main compound of the *Citrus* Spp. pericarp. It is studied that in DSS-induced colitis mice, it improves the reduction of colonic tissue damage, and increases the activity of the gut microbiota 109. Through oral administration, it also can inhibit the IL-12 and TNF-α expression, as other flavones can interact in the NF-κB pathway in UC attenuated 110. **Nobiletin**, a flavone found in citrus peels, exerted anti-inflammatory effects in TNBS-induced colitis through the downregulation of iNOS and COX-2 expression, restoring barrier function through the inhibition of the Akt-NF-κB-MLCK pathway 111. Lastly, **chrysin** is a flavone extracted from honey, propolis, and various plants, fruits, and even fungi 112,113. This polyphenol was able to prevent chemically induced colitis *in vivo* through the regulation of the PXR/NF-κB pathway 114.

Overall, these results support the relevance of flavones in the clinical management of IBDs, particularly demonstrated in vitro and *in vivo*. More studies in humans are still required.

#### 2.1.2. Flavonols

This group has the typical structure within the plane of the 3-hydroxyflavone base. They are different from the rest of the flavonoids because they only have one hydroxyl group at the C-3 position, also their O-glycoside is in the C-7 position 115. Their main representatives are quercetin and kaempferol; however other flavonols like galangin, myricetin, or isorhamnetin should also be highlighted.

##### A) Quercetin

**Quercetin** is one of the bioflavonoids with a wide range of uses in treating metabolic and inflammatory diseases. It is abundantly present in citrus, and green leafy vegetables like broccoli, flowers, and nuts 116. It is known to act in the intestinal by integrating the mucosa barrier, improving the increase of the colonic microbiota, moderating the oxidative stress response, and resettling the local immune homeostasis 117. Through a systematic review and meta-analysis, Hu *et al.* 118 concluded that preclinical evidence suggests that quercetin is a potential agent to consider in IBD treatment. In more detail, they observed that quercetine could reduce histological score, DAI, IL-1β, TNF-α, nitric oxide (NO), MDA, myeloperoxidase (MPO) activity and increase colon length, weight change degree, interleukin-10 (IL-10), glutathione (GSH), SOD and CAT. However, they observed that due to the low methodological quality and the small number of studies included some cautions must be considered with these results. Other preclinical works have also found significant anti-inflammatory effects of polyphenols in the management of IBD. For instance, quercetin seems to act *in vivo* through the modulation of intestinal microbiota, leading to the re-establishment of healthy microbiomes that favor mucosal healing, and the inhibition of PI3K/AKT signaling 119. Likewise, it was shown to balance the proportion between anti-inflammatory M2 and proinflammatory M1 macrophages, facilitating intestinal repair 120. In one study where quercetin is administrated orally in a water-soluble inclusion form, accompanied by hydroxypropyl-b-cyclodextrin (Que-HP-β-CD) in an experimental model of UC in mice, they show a therapeutic/prophylactic potential of this combination to treat the UC 121. On the other hand, in rat intestinal microvascular endothelial cells (RIMVECs), quercetin downregulates pyroptosis, the levels of inflammatory factors, and the elimination of the intestinal barrier produced by the lipopolysaccharide (LPS) by reducing the activation of the NLRP3 inflammasome 122. Quercetin was also able to repair intestinal barrier dysfunction *in vitro* by activating AhR-mediated enhancement of TJs to alleviate UC 123. Also, some studies have remarked on the relevance of quercetin as a non-toxic and safe bioactive compound with a marked antiviral activity, being suggested as a potential tool against viral-associated IBD 124. Similarly, quercetin appears to exert therapeutic effects on *C. rodentium*-induced colitis 125, suggesting the relevance of this polyphenol against viral and bacterial infections.

##### Observational and intervention studies in humans

Two observational studies have specifically focused on the relationship between quercetin intake and IBDs in humans. Lu *et al.* 126 analyzed a prospective cohort of 187,709 IBD-free participants from the UK Biobank collecting dietary information to estimate the daily quercetin intake. After almost 10 years of follow-up, they reported 863 incident IBD, finding that participants in the highest quintiles were associated with a lower risk of IBD and UC but not CD when compared to those in the lowest quintile 126. Similarly, Wang *et al.* 127 included 2,293 participants with IBD (764 with CD and 1529 with UC) from the UK Biobank and follow them for almost 10 years. During this period, they observed that patients with higher dietary intake of quercetin presented a lower risk of enterotomy and all-cause mortality in IBD when compared to patients located in the lowest quartile intake of quercetin.

One intervention study 128 evaluated the relevance of quercetin in patients with IBD. The study compared two groups of patients receiving different flavonoid mixtures for hemorrhoidal disease (diosmin, troxerutin, rutin, hesperidin, and quercetin as the study group versus hesperidin, diosmetin, isoroifolin, and linarin in purified micronized fraction as the control group. They report that both groups presented bleeding improvement with no significant difference between the groups after 1 and 6 months. However, patient satisfaction after 6 months was significantly higher in the study group receiving the mixture of diosmin, troxerutin, rutin, hesperidin, and quercetin. Therefore, these studies support a potential association between dietary quercetin intake with prevention and improved outcomes related to IBDs, although further intervention studies are required to evaluate the therapeutic effects of quercetin in humans. In a pilot study, Ryan *et al.* 129 aimed to identify the effects of a nutrition support formula on blood nutrient parameters in adults with IBD. The formula contained a mixture of micronutrients (including methylated forms of folate and vitamin B12), macronutrients, and phytonutrients (including curcumin, XN, ginger compounds, and quercetin). 10 participants with Crohn's disease or ulcerative colitis consumed a micronutrient and phytonutrient-rich beverage twice daily for 12 weeks. Significant increases in serum folate and decreases in red cell distribution width were observed. Modulation of leukocyte subtypes was noted, with a decrease in neutrophils and an increase in lymphocytes. Other parameters, including RBC count, hemoglobin, hematocrit, electrolytes, albumin, and inflammatory markers, did not change significantly.

##### B) Kaempferol

**Kaempferol** also known as 3,5,7-trihydroxy-2-(4-hydroxyphenyl)-4H-1-benzopyran-4-one, forms part of the flavonols group due to its properties as an anti-inflammatory, and anti-ulcerative properties, having multiple activities in different molecular pathways 130. This flavonoid is mainly found in many edible plants like tea, broccoli, cabbage, kale, beans, endive, leek, tomato, strawberries and grapes, and herbal medicinal plants (e.g. Ginkgo biloba, Tilia spp, Equisetum spp, Moringa oleifera, Sophora japonica and propolis) 131. Similar to quercetin, kaempferol seems to be effective at protecting colonic mucosa from DSS-induced UC 132. Qu *et al.* 133 showed that kaempferol may be relevant for treating IBD by regulating the gut microbiota and TLR4-related signaling pathways. In more detail, kaempferol seems to act by elevating the levels of ZO-1, occludin, and claudin-1, reducing the levels of IL-1β, IL-6, and TNF-α and the transcription of an array of inflammatory signaling molecules, accompanied by an increase in IL-10 mRNA expression. In RIMVECs is demonstrated that kaempferol reduces LPS-induced inflammatory mediators, such as TNF-α, IL-1β, IL-6, and vascular cell adhesion molecule-1 (VCAM-1), acting at the toll-like receptor 4 (TLR4), NF-κB, and STAT 134. Also, kaempferol reduces the IL-8 secretion and barrier dysfunction of the cell line Caco-2 monolayer by the LPS-induced epithelial-endothelial model through the blocking of the NF-κB signaling pathway 135. Apart from these effects, Kaempferol can balance the intestinal microbiome in mice by elevating the Firmicutes to Bacteroidetes ratio; increasing the linear discriminant analysis scores of beneficial bacteria, such as Prevotellaceae and Ruminococcaceae; and reducing the richness of Proteobacteria in DSS-challenged mice 133.

##### C) Other relevant flavanols

As previously mentioned, other important types of flavonols studied in IBD include galangin, myricetin, fisetin, or isorhamnetin. **Galangin** is a natural flavonoid isolated from ginger, gangal, honey, and propolis. This compound seems to act as an HSP90β inhibitor, a component significantly increased in the mucosal biopsies of UC patients and the colons of colitis mice, which show a direct correlation with disease severity 136. Indeed, galangin was shown to potentially alleviate colitis by inhibiting HSP90β and perturbing fatty acid synthesis-mediated NLRP3 inflammasome activation. Galangin demonstrated dose-dependent regulatory effects *in vitro*, reducing nitrites, IL-6, and TNF-α levels. *In vivo*, oral administration of galangin alleviated colitis, reduced proinflammatory cytokines (TNF-α and IL-6), increased anti-inflammatory IL-10, and decreased MPO, nitrites, and TBARS levels while increasing SOD 137. Other works have also found that galganin downregulates Toll-like receptor 4 (TLR4) expression, and suppresses NF-κB p65 activation 138, also leading to significant increases in autophagy proteins and recovery of beneficial bacteria like *Lactobacillus spp.*, and increased *Butyricimonas spp.* 139.

**Myricetin** is another flavonol mainly found in fruits, vegetables, berries, teas, and wine, including cranberry, dock, sweet potato leaves, chard, broad beans, and immature seeds the foods with the richest content in this polyphenol 140. Qu *et al.* 141 have found that myricetin can reduce the severity of inflammation in acute UC and significantly improve the condition. The administration of myricetin (80 mg/kg) increased the levels of IL-10 and TGFβ while augmenting the proportion of Treg cells. In a similar line, Zhao *et al.* found that myricetin administered orally at 200, 100, or 50 mg/kg to DSS-induced UC mice alleviated body weight loss in a dose-dependent manner and significantly reduced histology scores. Besides, myricetin decreased the production of NO, MPO, MDA, IL-1β, and IL-6 while increasing the activity of SOD and GSH 142. Likewise, other studies have found that myricetin and M10, a myricetin-3-O-β-d-lactose sodium salt can normalize Firmicutes and Actinobacteria populations, leading to a marked increase in Akkermansia muciniphila and decrease in pathogenic microorganisms, such as Ruminococcus and Parabacteroides 143. Some studies have found that M10 exerts higher activities in preventing UC through inhibiting necroptosis 143 however, it has also been demonstrated that the majority of M10 is metabolized to myricetin via fecal microbiota and that both compounds are mostly located in inflamed tissues, exerting their immunomodulatory actions 144.

**Fisetin** is abundantly found as a dietary flavonoid found in various fruits (strawberries, apples, mangoes, persimmons, kiwis, and grapes), vegetables (tomatoes, onions, and cucumbers), nuts, and wine 145. Past works have found that fisetin can inhibit senescence markers (p53, Bcl2, Cxcl1, and Mcp1)in DSS-induced UC in mice, upregulating the expression of micro RNAs (miRNAs) miR-149-5p, miR-96-5p, miR-34a-5p, and miR-30e-5p and the abundance of *Akkermansia muciniphila*, which is negatively correlated with senescence and inflammation 146. Likewise, fisetin may exert an important anti-inflammatory activity via inhibition of Akt, p38 MAPK, and NF-κB signaling in the colon tissues of DSS-exposed mice, also enhancing GSH and reducing MDA levels 147.

**Isorhamnetin** glycosides are primarily extracted from various plant-based foods or medicinal plants such as *Opuntia ficus*-indica, *Hippophae rhamnoides, and Ginkgo biloba* 148. Animal models have shown that isorhamnetin can alleviate IBD via PXR-mediated up-regulation of xenobiotic metabolism and down-regulation of NF-κB signaling 149. Isorhamnetin can also inhibit ferroptosis, a special type of programmed cell death mediated by iron independent of its previously reported targets MEK1 and PI3K, but alleviated oxidative stress by targeting and activating NRF2.

#### 2.1.3. Flavanones

Their structure is based on the generic structure of flavonoids, a flavan nucleus formed of two aromatic rings linked through a dihydropyrone ring (2,3-dihydro-2-phenylchromen-4-one). They are particularly abundant in fresh fruits and citrus 150. The most relevant flavanones studied in IBDs include naringin, naringenin, hesperidin, hesperitin, eriodictol, and to a lesser extent, eriocitrin and poncirin.

##### A) Naringin and naringenin

**Naringin** (4′,5,7-trihydroxyflavanone-7-rhamnoglucoside) and its aglycone form **naringenin** are two flavanones found mainly in citrus fruits, including lemon, orange, mandarin, and grapefruit 151. Preclinical models have shown that naringin can ameliorate the pathogenic symptoms of UC by inhibiting inflammatory response and regulating intestinal microbiota *in vivo* Among other mechanisms, naringin can improve DAI, colon length shortening, and pathological damage, decrease tissue and serum secretion of inflammatory cytokines, as well as the oxidative stress markers 152. Similarly, treatment with naringin significantly increased rat body weight and various hematological parameters including hemoglobin, red blood cells, and platelet count, while decreasing spleen weight, colon weight, colon weight to length ratio, macroscopic score, adhesion score, diarrhea score, stool consistency, rectal bleeding score, and white blood cell count 153. Naringin also significantly increased colonic levels of SOD, GSH, and CAT, while decreasing MDA, xanthine oxidase (XO), colonic NO, and MPO levels 153,154. Some of the underlying mechanisms associated with the beneficial effects of naringin include the stimulation of PPARγ and the inhibition of the NF-κB and the NLRP3 inflammasome 155,156. Likewise, naringin also increases the expression of TJ proteins and the relative abundance of Firmicutes/Bacteroides while reducing the content of Proteobacteria to improve the intestinal flora disorder caused by DSS 157. Because of the pleiotropic effects of naringin, some studies have also evidenced that this compound can prevent important medical complications associated with IBDs. For instance, Liu *et al.* 158 found that this polyphenol can attenuate intestinal fibrosis, a common complication associated with CD. Similarly, naringin was able to prevent colorectal carcinogenesis by suppressing robust ER stress-induced autophagy in colorectal mucosal cells 159. Li *et al.* 160 have also found that naringin may have great potential for the treatment of bone loss in glucocorticoid-treated IBD rats via blocking oxidative stress and promoting bone formation, whereas combined effects of poncirin and naringin from *Poncirus trifoliata* extracts can alleviate depressive behavior in DSS-induced models of colitis 161.

On the other hand, naringenin is suggested to act as an important immunomodulator against T cell-mediated autoimmune diseases like IBDs 162. Naringenin was also able to alleviate acetic acid-induced UC in rats in a dose-dependent manner, increasing colonic mucus content and reducing the expression of various inflammatory and oxidative stress markers 163,164.

##### B) Hesperidin and hesperitin

**Hesperidin** and its aglycone form, hesperetin can be richly found in citrus fruits such as lemon, sweet oranges, bitter oranges, citron, clementines, and mandarins as well as in *Menthae piperitae*, *Hypericum perforatum*, and *Salvie officinalis* 165. Treatment with hesperidin significantly reduced neutrophil infiltration, edema, colon shortening, and macro and microscopic damages induced by intracolonic administration of acetic acid in mice 166. The improvement of colitis after hesperidin treatment is related to the inhibition of pro-inflammatory cytokines TNF-α, IL-6, IL-1β, and IL-33 as well as NF-κB activation in the colon. Likewise, hesperidin can alleviate colonic sphingosine phosphate phosphatase 2 messenger RNA expression and sphingosine kinase-1 levels, thus suppressing the subsequent downstream inflammatory and apoptotic cascades represented by decreased macrophage inflammatory protein-1α and enhancement of B-cell lymphoma 2 immunohistochemistry expression. While improving mitochondrial biogenesis by increasing the peroxisome proliferator-activated receptor-gamma-coactivator 1-α level 167. Similarly, this marker can act as a potent antioxidant evidenced by marked alleviations of the NO and peroxynitrite levels, increasing total antioxidant capacity, and activating the SOD enzyme 166,167 also improving DAI, MPO activity and MDA content 168.

Regarding **hesperitin**, past works have evidenced that this compound may ameliorate DSS-induced colitis by maintaining an epithelial barrier via blocking the intestinal epithelial necroptosis 169. Hesperetin was also shown to alleviate TNBS-induced ulcerative colitis through antioxidant (increasing GSH and SOD while decreasing NO content), anti-inflammatory properties (reducing IL-6, TNF-α, CD45 and NF-kB), antiapoptotic (diminishing caspase 3 and Bax expression) and through modulating JAk2/STAT3/SOCS3 170,171.

##### C) Eriodictyol, eriocitrin and poncirin

**Eriodictyol** is another polyphenol abundantly found in citrus fruits, vegetables, and most of the medicinal plants 172. Eriocitrin is prominently found in lemons 173 and poncirin in hardy oranges and mandarins 174. Previous studies have demonstrated that erodicytol is able to decrease MPO expression and regulate the cytokine parameters and oxidative stress in TNBS-induced intestinal tissues of rats. Specifically, the levels of TNF-α, IL-1β, IL-6, IL-10, IL-2, and IL-12 SOD, CAT, GSH-Px, and MDA were modulated in rats with colitis, also inhibiting TLR4/NF-κB pathway activation 175. Likewise, it seems to upregulate the Sonic hedgehog (Shh) pathway, reducing DAI, colon shortening, histological score, and apoptosis in the colon while augmenting the expression of the tight junction proteins ZO-1 and occluding 176. One study shows that **eriocitrin** (30 mg/kg) demonstrated significant attenuation activity against the DSS-stimulated severe colitis in experimental animals, counteracting body-weight loss, colon shortening, histopathological injury, inflammatory cells infiltration, and the secretion of inflammatory cytokines 177. Together with naringin, the use of **poncirin** from *Poncirus trifoliata* extract seems to exert antidepressant effects in mice by restoring vascular endothelial cell integrity in the hippocampus and controlling the neuroinflammatory responses of microglia at the Cornu Ammonis 1 (CA1) and dentate gyrus (DG) regions of the hippocampus 178.

#### 2.1.4. Isoflavones

They are mostly defined as phytoestrogens because they present a chemical structure similar to human estrogen, acting as a physical mimic of natural estrogens by binding to their receptors 179. They are also considered polyphenols because of their chemical structure, which is formed by two benzene rings (A and B rings) linked with a heterocyclic pyran ring (C ring) 180. The most important dietary sources of isoflavones are soybeans and soy derivatives, although they can also be found in other legumes such as green beans, and mung beans and in various medicinal plants 181. Daidzein and Genistein are the most relevant isoflavones explored in the context of IBD, although other isoflavones like glycitein, formononetin, biochanin A, equol, and irilone should also be mentioned herein.

##### A) Daidzein

Daidzein is a critical isoflavone with pleiotropic effects in intestinal cells. Apart from soy and legumes, currants and raisins are another important source of both daidzein and genistein 182. *In vitro,* studies have shown that this compound is able to upregulate metallothionein gene expression and induce CAT activity while decreasing SOD activity in unstimulated Caco-2 cells, but not when the cells were challenged with lipid hydroperoxides 183. Likewise, other works have also found that daidzein can also improve TJ integrity in Caco-2 cells 184, also being able to attenuate LPS-induced inflammatory responses from intestinal cells, interfering with NF-kB-dependent molecular mechanisms 185.

One of the derivates of Daidzein the 8-Hydroxydaidzein can also play a role as an anti-inflammatory compound in activated macrophages such as RAW 264.7 cells by controlling the proinflammatory cytokines and NF-κB pathway, suggesting that Daidzein could block DSS-induced UC and reducing inflammatory factor expression 186. Another study found that Daidzein interacted with soybean meal diet-induced intestinal inflammatory responses, in the anti-inflammatory response of the action of Daidzein involved p38, JNK, and NF-κB pathways, leading Daidzein to act as an antioxidant to resist the oxidative damage produced 187. Daidzein-rich isoflavone aglycones administered to mice for 1 week before inducing UC by DSS leaded to decreased inflammation and tissue damage in the colon than the control mice 188. More specifically, a decrease in various cytokines such as interferon-gamma, IL-6, and IL-12p40 secretion, and an increase in IL-10 secretion was observed, along with low cell-activation status of antigen-presenting cells (APC) and an inhibition of IL-6 and IL-8 production by TLR2 and TLR4-stimulated monocytes in a dose-dependent manner. Similarly, supplementation with daidzein was shown to reduce the level of myeloperoxidase MPO and inhibit the expression of TNF-α, IL-1β, and IL-6, in the colonic tissues, inhibiting the production of NO and prostaglandin E2 in LPS-stimulated RAW 264.7 macrophages 189.

##### Observational studies in humans

Observational studies conducted in humans have obtained interesting results regarding the association of daidzein with IBDs. Skolmowska *et al.* found that high intake of daidzein and tota [190]l isoflavones seemed to reduce the mucus in the feces of UC patients; whereas, a high intake of daidzein alone might drive to an increased fecal pus. In a similar line, Ohfuji *et al.* 191 found that dietary isoflavone consumption seemed to be associated with an increased risk of UC, particularly in females. It should be highlighted that whereas the study of Skolmowska *et al.* was conducted in European subjects, the work made by Ohfuji *et al.* was developed in Japan, existing significant differences in the consumption of isoflavones per day across these regions (25,000-50,000 µg of isoflavones/day in Japan and <1000 µg in Europe) 192. In agreement with this, Głąbska *et al.* 193 also found in European patients with UC in remission a direct association between lack of gastrointestinal pain with higher intakes of daidzein, daidzein per 1000 kcal of diet and total isoflavone consumption when compared to those reporting abdominal pain. Therefore, the dose is a relevant factor to consider regarding the role of daidzein and polyphenols in general in human health and IBDs.

##### B) Genistein

**Genistein** is shown to be involved in reducing DSS-induced colitis, and also slants M1 macrophages to an M2 phenotype, suggesting that genistein works to be part of the treatment of IBD 194. Chen *et al.* 195 also found in DSS-induced colitis mice that genistein was able to inhibit NLRP3 inflammasome via TGR5-cAMP signaling in macrophages. Genistein increased LPS-induced COX2 expression and decreased LPS-induced phosphorylation of IκBα in IEC18 cells 196. In the colon, expression of COX-2 mRNA and protein was reduced after geinistein treatment together with a decrease in MPO activity 197. Both genistein and daidzein seemed to inhibit signal translation and activator of transcription 1 (STAT-1) leading to decreased expression of iNOS 198. Genistein at high concentrations (300µM) can also prevent oxidative stress-induced tyrosine kinase-mediated phosphorylation of the TJ proteins occludin and ZO-1, preventing the breakdown of the intestinal epithelial barrier in Caco-2 cells 199. In these cells, genistein reduced (4- to 8-fold) IL-1beta-induced IL-8 secretion but promoted NF-kB activity 200. Genistein can also exert anti-inflammatory effects through the modulation of the gut microbiota. For instance, past works have found that genistein can reduce the growth rate of *Lactococcus lactis subsp. lactis, Slackia equolifaciens, and Bacteroides fragilis*, while augmenting the growth rate of *F. prausnitzii* and *Lactobacillus rhamnosus*, being these changes associated with increased SCFA production 201. In line with this, Jia *et al.* claimed that the benefits of genistein in macrophage polarization balance can also be attributed to their associated improvements in intestinal microbiota and its metabolites like SCFAs in DSS-induced UC in mice 202. UC rats treated with 25-mg/kg genistein showed improved inflammatory cell infiltration, hemorrhage, and destruction of intestinal glands by enhancing the expression of peroxisome proliferator-activated receptor-gamma coactivator (PGC-1), mitochondrial transcription factor A (TFAM), nuclear factor erythroid 2-related factor-2 (Nrf2), heme oxygenase-1 (HO-1), and BCL2 and reduced the expression of BAX, caspase-3, caspase-8, and caspase-9 203. Likewise, genisteine can also reduce the activation of the INF-γ/JAK1/STAT1 and INF-γ /TLR-4/ NF-κB signaling pathways and modulate the IRF-1/iNOS/NO and IL-6/JAK2/STAT3/COX-2 pathways and consequently, reduced the levels of TNF-α and IL-1β 204. Furthermore, it seems that when combined with EVOO, genistein showed more beneficial effects in decreasing inflammation in comparison with pure oils or genistein alone 205

On the other hand, some negative effects related to genistein have also been observed. For instance, high prenatal and postnatal exposure to genistein and daidzein (genistein: 240 μg/g feed; daidzein: 232 μg/g feed) seemed to enhance acute inflammation markers, influencing the expression of MPO and COX-2 when compared to those having a very low intake of both genistein and daidzein (<10 μg/g feed) 206. In another study employing human colonic organoids (hCOs), genistein exerts its detrimental effects on the intestinal mucosa via negative regulation of stem/progenitor cell function 207. More studies are warranted to find proper doses before considering the administration of both genistein and daidzein in IBD patients.

##### C) Other isoflavones: Glycitein, formononetin, biochanin A, and irilone

Glycitein, formononetin, biochanin A, and irilone are also important isoflavones mainly found in soybeans in the case of the former and red clover in the case of the latter 208. The relevance of these isoflavones in IBDs has been less studied than the previously mentioned.

Regarding **glycitein**, molecular docking demonstrated that this compound together with other polyphenols plays a critical role in treating UC with Fuzi-Lizhong Pill (FLP) and Huangqin Decoction (HQT) 209. Similarly, this compound was also identified as a key modulator of the activity of Codonopsis pilosula, responsible for alleviating UC through the inhibition of the PI3K/Akt signaling pathway 210. Finally, Głąbska *et al.* 211 observed that patients with UC in remission reported a lack of constipation and lower intakes of glycitein and glycitein per 1000 kcal of diet.

**Formononetin** has also been recognized as a critical bioactive compound explaining the success of UC therapy of FLP and HQT 209, Sijunzi Decotion 212, Radix Astragali 213, *Sophora flavescens* 214,215, *Hedysarum multijugum* 216, *Lizhong Decotion* 217 and hydroalcoholic extract of Brazilian red propolis (HERP), also rich in daidzein and biochanin A 218. **Biochanin A** inhibited the elevation of ROS, IL-1β, IL-18, and TNF-α release, nitrite production, and the expression of iNOS and COX-2 in RAW 264.7 cells under LPS stimulation 219. In Muc2-/- mice, biochanin A alleviated UC by restoring the intestinal barrier and promoting autophagy (upregulating TJ proteins, AMPK/mTOR/ULK1 pathway), inhibiting apoptosis and favoring proliferation through reducing caspase 3 expression, and increasing PCNA and Ki67 levels 220. Biochanin A has also been shown to ameliorate UC in DSS mice thanks to its anti-inflammatory activity by inhibiting the MAPK/NF-κB (p65) axis 221.

On the other hand, **equol** is a bacterial metabolite of isoflavones with multiple health benefits associated 222. The role of equol in IBDs however is still controversial. For instance, Sakai *et al.* 223 described that equol promoted DSS-induced UC in mice by downregulating the production of IL-10 by T cells. Conversely, Li *et al.* observed that indole 3 acetic acid, an indole derivative, alleviates DSS-induced colitis by promoting the production of Equol from *Bifidobacterium pseudolongum* 224. Also, it is known that equol can lead to an increased growth rate of *Lactobacillus rhamnosus*, a bacteria with anti-inflammatory effects and a protective effect on the intestinal barrier 225.

#### 2.1.5. Flavanols

They form part of the flavonoids group thanks to their C2 and C3 rings, they do not present a double bond between them, and the absence of a carbonyl group on the C4 ring. In nature, flavanols are divided into four groups: flavan-3-ols, flavan-4-ols, isoflavan-3,4-ols and flavan-3,4-ols. The most used of them is flavan-3-ols, followed by flavan-4-ols. They have a wide range starting with simple monomers and going through oligomers and also divided into aglycones or glycosides 226. Flavanols are found in many foods, including cocoa, tea, cereals, legumes, fruits, vegetables, forages, hops, beers, red wine, grapes, and apples. The main flavanols explored in IBDs include catechins and proanthocyanidins/procyanidins.

##### A) Catechins

**Catechins** are natural polyphenols that are present in green tea, cocoa beans, and grapes 227. Catechins are formed by **epicatechin (EC), epicatechin gallate (ECG), epigallocatechin (EGC), and epigallocatechin-3-gallate (EGCG)** 228,229. Their natural properties control the infiltration and proliferation of immune cells like colonic epithelial cells, macrophages, T lymphocytes, and neutrophils 230. As the other flavonoids, they exert an anti-inflammatory activity increasing or decreasing the inflammation induced by oxidative stress, through different vias such as MAPK NF-κB, Nrf2, and STAT 1/3 pathways. Also, it is demonstrated that catechins have important modulatory effects on the gut microbiota, which could be of great relevance in the management of IBD. Among other changes, catechins can kill certain pathogenic bacteria, like *Clostridium perfringens, Erwinia, Pseudomonas, Clavibacter, Xanthomonas, Agrobacterium spp. Staphylococcus spp., Vibrio parahaemolyticus, Bacillus cereus*, and *Plesiomonas shigelloides*, promoting the growth of beneficial bacteria, like *Bifidobacterium spp.* in human studies while modulating the proportion of Firmicutes, Bacteroidetes, and other bacterial phyla *in vitro* 231.

Specifically, **EC** was shown to ameliorate inflammation in animal models of UC through the inhibition of NF-κB activation along with a decrease in TNF-α, IL-6, NO, MPO, and MDA and an increase in antioxidant enzymes 232. However, most studies have focused on **EGCG** as a potential treatment for IBDs. EGCG forms part of the catechins and their biological activity in IBD is related to its anti-inflammatory properties 233. Yu *et al.* 234 showed that both high- and low-dose EGCG treatment (50 mg/kg/day and 20 mg/kg/day, respectively) alleviated body weight loss and DAI of DSS-induced colitis, preventing colon shortening and improving intestinal permeability and histopathological changes. Moreover, EGCG treatment attenuated colon inflammation by reducing the levels of pro-inflammatory cytokines IL-6, MCP-1, and TNF-α, and inhibiting CD3+ T cell and CD68+ macrophage infiltration. These results were supported by a preclinical meta-analysis conducted by Wei *et al.* 235 that included 19 studies involving 309 animals, showing that EGCG was associated with a decrease in IL-1β, IL-6, and interferon-γ; together with alterations in MDA, SOD, GSH, and CAT levels. Besides, they claimed that oral administration exhibited superior efficacy over other forms of administration, whereas the optimal dosage range was 32-62 mg/kg/day, with an intervention duration of 4.8-13.6 days in animals. However, more studies are still warranted in humans. Other works have found synergic effects of EGCG with peracetylated-epigallocatechin-3-gallate (AcEGCG) 236 and quercetin 237.

##### B) Proanthocyanidins and procyanidins

**Proanthocyanidins** (also known as condensed tannins) are compounds formed by the polymerization of flavan-3-ol (catechin, epicatechin, epigallocatechin, and epiafzelechin) commonly found in fruits, nuts, bark, chocolate, wine, and some plant seeds and flowers 238. Depending on the type of monomers, PACs can be classified into procyanidins, prodelphinidins, and propelargonidins. **Procyanidins** are the most common types of proanthocyanidins and are exclusively formed by catechin and epicatechin molecules 239. Prodelphinidins and propelargonidins are composed of (-)-gallocatechin/(-)-epigallocatechin and (+)-afzelechin/(-)-epiafzelechin monomers, respectively, although they are notably less distributed in nature 240. Procyanidins can be categorized into A-type and B-type depending on the stereo configuration and linkage between monomers. Consequently, there are different types of A and B-type procyanidins, being procyanidin A1 and A2, B1, B2, B3, and B4 the most common members 241.

The use of **grape seed proanthocyanidin extract (GSPE**) has demonstrated multiple benefits in animal models of UC. Wang *et al.* 242 demonstrated in DSS-induced UC in mice that after administration of GSPE the serum and colonic tissue levels of IL-1β, IL-6, TNF-α, NF-κB, Keap-1 NO, and MDA decreased, whereas SOD, Nrf2 and HO-1 proteins content increased. They reported that catechin, epicatechin, and procyanidins B1, B2, and B4 were mostly responsible for the beneficial effects observed. In a similar line, Sheng *et al.* 243 also found that GSPE improved DAI, pathological scores, and oxidative stress in these animals and promoted upregulation of ZO-1 occludin, and claudin-1 mRNA levels of colon tissue. Likewise, the expression level of proinflammatory cytokines and the NLRP3 inflammasome mRNA levels of colon tissue were also reduced, whereas 16S rRNA analysis showed that GSPE rebalanced the gut microbiota, by reducing Bacteroidetes, Dubosiella, and Veillonella, increasing Verrucomicrobia and Akkermansia, and elevating the Firmicutes to Bacteroidetes ratio. Chu *et al.* also reported that GSPE elevated the expression of anti-inflammatory cytokine IL-10 in the colon tissues and serum of DSS-induced colitis mice by suppressing the NF-κB signaling pathway, being able to ameliorate LPS-induced inflammation in RAW264.7 cells 243. In canins with IBDs, favorable effects from GSPE on the gut microbiota were also accompanied by improvements in bile acid metabolism 244. **Persimmon-derived proanthocyanidins** can also ameliorate colon inflammation, DAI, and macrophage activation in DSS-induced UC mice, while influencing gut microbiota composition, leading to an increase of alpha diversity and Bacteroidetes along with a decrease in Enterobacteriaceae and Enterococcus 245. Importantly, it should be noted that the microbiota and polyphenols have a bidirectional interaction that should be considered. For instance, the metabolism of proanthocyanidin dimers from cranberries can also be affected by an altered microbiota associated with UC, as shown by Diaz *et al.* 246, thus affecting the response to this compound.

Regarding **procyanidins**, various studies have shown promising results from their use in *in vivo* and *in vitro* models. Previous works have found that procyanidins can prevent the polarization of macrophages to the M1 type and downregulate the levels of proinflammatory factors in cells, whereas *in vitro* studies found that this effect was attributed to the regulation of the STAT3 and NF-κB pathways 247. Other *in vitro* models have also shown that procyanidins can have a significant effect on ROS clearance, also suppressing the expression of MMP9, NF-κB, and the NLRP3 inflammasome in colonic tissue of DSS-induced UC mice 248. Procyanidins from the peanut skin (PSP) can also attenuate DSS-induced UC inflammation in mice by increasing the relative abundance of Clostridium XlVb and Anaerotruncus, along with SCFA production and reducing the relative abundance of Alistipes at the genus level 249. PSP is particularly rich in procyanidins A, which are shown to modulate inflammatory (TNF-α, IL-β, IL-6, and IL-10) and oxidative stress markers (MDA, T-SOD, NO, and iNOS) in DSS-induced UC mice and an increase in the relative abundance of Lachnospiraceae_NK4A136_group, Oscillibacter, and Roseburia and a decrease of Bacteroides, Helicobacter, Parabacteroides, Escherichia-Shigella, and Enterobacter after PSP treatment 250. Procyanidin A can also influence DSS-induced colitis in mice, procyanidins exert their activity in the AMPK/mTOR/p70S6K pathway by decreasing levels in p-p70S6K and p-mTOR in this pathway 251. Procyanidin B2 seems to suppress oxidative stress by modulating Nrf2/ARE signaling 252, whereas it can also alleviate intestinal inflammation and protect intestinal mucosal functions and structural integrity by inhibiting intestinal PI3K/AKT signaling pathway 253.

#### 2.1.6. Anthocyanins and anthocyanidins

**Anthocyanins** are found as a red pigment in a variety of berries, currants, and grapes, and consist of an anthocyanidin bound to one to three sugar molecules like arabinose, galactose, glucose, rhamnose, and xylose 254. Therefore, **anthocyanidins** are the aglycone form of anthocyanins, and the main types are cyanidin, delphinidin, pelargonidin, peonidin, petunidin, and malvidin 255. In general, anthocyanins are only partially absorbed and have shown limited biological activity in enterocytes 256. Numerous studies have focused on the antioxidant properties of anthocyanins, but their anti-inflammatory effects have also been extensively studied in non-intestinal tissues 257. Research has demonstrated radical scavenging and modulatory activities in the gut microbiota from natural compounds containing anthocyanins 258,259. Anthocyanin-rich fruits can be divided into three groups based on the types of aglycones of their anthocyanins: pelargonidin group, cyanidin/peonidin group, and multiple anthocyanidins group. This fact can have important implications as compelling evidence suggests a trend for fruits rich in cyanidin, peonidin, or pelargonidin glycosides to exhibit more reproducible-anti-inflammatory effects than blueberries, which contain mostly delphinidin, malvidin, and petunidin glycosides 260. Delphinidin, malvidin, and petunidin glycosides are unstable and can be further fragmented into smaller molecules, being this fact possibly associated with these observations.

However, a broad spectrum of studies have evaluated the role of anthocyanins in cellular and animal models of IBD. Anthocyanins are generally used from berry extracts, including barberry, bilberry, blueberry, mulberry, raspberry, or strawberry, although purple (sweet) potato or dark-purple rice extracts or red cabbage have also been used.

#### A) Barberry, bilberry, bulberry, and cranberry anthocyanins

**Barberry anthocyanins** belong to the *Berberidaceae* family and consist of full anthocyanins, their main property is their ability to reduce macroscopic ulcer index and ulcer area, colon wet weight/length ratio, and inflammation-inducing cell infiltration 261.

**Bilberry and blueberry** anthocyanins are members of the *Ericaceae* family, and as aforementioned, the anthocyanins are mainly malvidin, delphinidin, and petudin, although they also have an important content of cyanidin 262,263. In *Drosophila melanogaster*, anthocyanins from bilberries seem to ameliorate DSS-induced UC and improve the antioxidant capacity by modulating NRF2 signaling pathways 264. Oral administration of bilberries reduced disease severity and inflammation in both acute and chronic DSS-induced colitis mice, decreasing IFN-γ and TNFα secretion. Both bilberries and their anthocyanins prevented apoptosis in colonic epithelial cells caused by inflammation 265. On the other hand, treatment with blueberry extract promoted a significant decrease *in vitro* in nuclear and cytoplasmic generated ROS compared to controls, also increasing cell viability following treatment with the pro-inflammatory cytokines 266. Pereira *et al.* compared the efficacy of anthocyanin-rich fraction from Portuguese blueberries and 5-aminosalicylic acid in the TNBS-induced UC rat model. They observed that despite both agents exerting anti-inflammatory and antioxidant properties, the greater actions of anthocyanins to downregulate iNOS to decrease leukocyte infiltration and to increase antioxidant defenses in the colon may account for the much higher anti-inflammatory action of anthocyanins 267.

**Cranberry** is also a member of the Ericaceae family plant. Differentially from bilberries and blueberries, the main anthocyanins in cranberry samples are cyanidin and peonidin glycosides 268. However, their use in animal models of IBD has also been evaluated in the past. Xiao *et al.* 269 reported that both cranberry extract and dried cranberries significantly reduced DAI in a murine colitis model, with dried cranberries demonstrating superior efficacy in preventing colitis and mitigating inflammatory markers compared to cranberry extract, suggesting the potential utility of cranberries in IBD prevention and symptom reduction. Most of the benefits associated with the anthocyanins and other polyphenols of cranberries are attributable to their regulatory role on gut microbiota. Dietary cranberry supplementation was shown to mitigate DSS-induced alterations in fecal microbiota, increasing the abundance of beneficial bacteria such as Lactobacillus and Bifidobacterium while decreasing potentially harmful bacteria such as Sutterella and Bilophila 270. Zhang *et al.* 271 investigated the effects of cranberry concentrate Type M (CTM) on adherent-invasive Escherichia coli (AIEC) LF82, associated with CD at different infection stages, revealing significant reductions in AIEC LF82 levels in a simulated mucus layer with 0.5 and 1 mM CTM concentrations. Moreover, both fermented and unfermented CTM at 1 mM demonstrated decreased adhesion and invasion of AIEC LF82 in human-derived Caco-2 epithelial cells, indicating potential antipathogenic effects mediated by gut microbiome modulation. It should also be noted that the gut microbiota can also exert a significant influence on the effects of cranberries. Sirven *et al.* 272, compared the metabolism of cranberry polyphenols between healthy individuals and those with UC, revealing that healthy microbiomes generated higher concentrations of specific metabolites, possibly due to differences in microbiota composition.

#### Intervention studies in humans

Biedermann *et al.* 273 tested the effect of a daily standardized anthocyanin-rich bilberry preparation in a pilot study on 13 patients with mild to moderate UC after a follow-up of 9 weeks. They reported that at the end of the 6-week treatment interval, 63.4% of patients achieved remission, while 90.9% of patients showed a response. Equally, they observed a decrease in total Mayo score in all patients, whereas fecal calprotectin levels significantly decreased during the treatment phase. Despite no serious adverse outcomes observed, an increase in calprotectin levels and DAI was noticed after cessation of bilberry intake. Roth *et al.* 274 also studied the molecular mechanisms and effects of anthocyanins extracted from bilberries *in vitro* by analyzing colonic tissue and serum samples of 13 mild to moderate UC patients treated with an oral anthocyanin-rich bilberry preparation during an open-label clinical trial. The histopathological analysis determined that reduced amounts of the pro-inflammatory cytokines IFN-γ, TNF-α, and phosphorylated (activated) p65-NF-κB were reduced in these patients. Likewise, responsive patients to the received treatment showed enhanced levels of Th17-cell specific cytokine IL-22 and immunoregulatory cytokine IL-10 as well as reduced serum levels of TNF-α and MCP-1, but enhanced levels of IL-17A, in contrast to patients that did not reach remission after the use of this compound.

#### B) Mulberry, raspberry, and strawberry anthocyanins

**Mulberry** (Morus alba L.) is a moraceous plant rich in many anthocyanins, highlighting cyanidin-3-O-glucoside, followed by cyanidin-3-O-rutinoside 275. Mulberry anthocyanins have also proven significant effects on IBD. Mo *et al.* 276 demonstrated that these compounds can inhibit DSS-induced clinical symptoms and colonic damage, reduce intestinal inflammation and oxidative stress, restore intestinal barrier integrity, and maintain immune homeostasis. Likewise, these compounds were able to regulate the structure of intestinal microbiota by reducing the level of potentially harmful bacteria (Escherichia-Shigella) and enriching the relative abundance of beneficial bacteria (Allobaculum, Akkermansia, and Muribaculaceae) 276.

**Black and red raspberries** are two Rubus species with differential content in anthocyanins. Black raspberries seem to have a higher content of anthocyanins, being cyanidin-3-xylosyl-rutinoside as the most common member 277. Red raspberries contain ∼92.1 ± 19.7 mg anthocyanins/100 g of fresh fruit in a ratio of 32:1 cyanidin- and pelargonidin-based anthocyanins 278. The relevance of the anthocyanins of both raspberries has been supported in past works. Montrose *et al.* 279 found that dietary intervention of freeze-dried black raspberries (BRB) improved body mass maintenance and reduced colonic shortening and ulceration in DSS-induced UC mice, but did not affect plasma NO or colon MDA levels. It suppressed several pro-inflammatory cytokines and significantly reduced colonic phosphor-IκBα and cyclooxygenase 2 levels, along with plasma prostaglandin E₂. Similarly, Huang *et al.* 279 also reported that BRB reduced colonic inflammation in IL-10 knockout mice by correcting dysregulated TLR-4 signaling, which downregulated PGE2. BRBs also decreased spleen macrophage percentages and altered plasma levels of inflammatory mediators, reducing PGE2 and PGI2 while increasing 15-lipoxygenase and its product, 13-S-hydroxyoctadecadienoic acid. Wang *et al.* also determined whether black raspberries (BRBs) affect promoter methylation of Wnt pathway suppressors in DSS-induced UC. BRB-fed mice showed reduced ulceration, decreased macrophage and neutrophil staining, and decreased NF-κB p65 nuclear localization. By day 7, BRBs demethylated the dkk3 promoter, increasing its mRNA expression, and decreasing levels of β-catenin, c-Myc, DNMT3B, HDAC1, HDAC2, and MBD2 in the colon and bone marrow 280. Moreover, another study 281 also found that BRB inhibited colonic ulceration and, ultimately, colon cancer partly through inhibiting aberrant epigenetic events that dysregulate Wnt signaling. Regarding red raspberry (RB), one work 282 found that RB supplementation reduced body weight loss, DAI, and colon shortening in DSS-treated mice, and protected colonic structure by suppressing NF-κB signaling and reducing inflammatory markers. It also decreased neutrophil infiltration, MCP-1 mRNA expression, and xanthine oxidase content, while enhancing catalase, claudin-3, ZO-1 protein, MUC-2 mRNA, and AMPK activation. In more detail, Bibi *et al.* 283 observed in the same animal models that RB supplementation reduced the DAI score and histologic damage by 38.9%, decreased inflammatory mediator expression by 20-70%, CD4 T cell infiltration by 50%, and α4β7 integrin and related adhesion molecules by 33.3%. RB also promoted epithelial repair, goblet cell density, and expression of Klf4, Hes1, Muc2, and intestinal alkaline phosphatase by 20-200%, while reducing proliferating cell nuclear antigen by 70% and β-catenin and STAT3 signaling by 19-33%, enhancing p53 stability and reducing oncogenic gene expression by 50-60%. Overall, these studies suggest that BRBs and RBs are promising agents to consider for the clinical management of IBDs, although clinical studies in humans are still warranted.

**Strawberries** belong *Rosaceae* family and their anthocyanins are mainly derived from pelargonidin and cyanidin aglycones 284. Some animal studies have found that the supplementation of anthocyanins from strawberries, through oral and rectal administration, reduced significantly the inflammation focus and mitigated epithelial necrosis and lesions 47,285,286. Ghattamaneni *et al.* 287 evaluated the possible role of pelargonidin 3-glucoside (P3G)-enriched strawberry added to the diet for the final 6 weeks in IBD rats to provide a dose of 8 mg P3G/kg/day. They observed that P3G consumption reversed DSS-induced UC with healthy stools and mucosal lining of the ileum and colon including increased villi, crypts, and goblet cells and reduced inflammation. Thus, despite clinical evidence being needed, anthocyanins from strawberries also seem an interesting option to treat IBDs.

#### C) Other sources of anthocyanins: Purple (sweet) potato, black/purple rice extracts, and red cabbage

Anthocyanins can also be found in other foods rather than berries, including purple (sweet) potato and dark/purple rice. Pigmented potato (*Solanum tuberosum L*.) and purple sweet potato (*Ipomoea batatas L.*) are two major sources of anthocyanins. The former has abundant acylated derivatives of anthocyanins 288, whereas the latter is one of the major sources of anthocyanins with a content ranging from 3.31 to 13.90 mg/g of fresh weight depending on the variety, climate, soil, and harvest conditions 289. Animal models have shown that purple-fleshed potato supplementation prevented DSS-induced weight loss, colon shortening, and increases in spleen and liver weights in mice 290. It reduced intestinal permeability, colonic MPO activity, pro-inflammatory interleukins (IL-6 and IL-17), pathogenic bacteria, and flagellin levels, with P25 also decreasing systemic MPO and increasing *Akkermansia muciniphila*. In a similar line, Li *et al.* also found that supplementation with purple- or red-fleshed potatoes at 20% w/w mitigated the DSS-induced reduction in colon length and mucin 2 expression levels, and the increase in permeability, spleen weight, myeloperoxidase (MPO) activity, and expression levels of inflammatory cytokines (IL-6, IL-17, and IL1-β) in non-antibiotic mice, but not in gut microbiota ablated mice 291 In a recent study, Sun *et al.* 292 divided six-week-old C57BL/6J male mice into two groups, one receiving a standard diet and the other with 10% purple potato powder, for 7 weeks. At week 5, each group was subdivided into two, with or without 2.5% DSS induction for 7 days, followed by 7 days of recovery. Purple potato supplementation improved DAI, reversed colonic damage induced by DSS, restored tight junction proteins and homeobox 2 levels, and enhanced mitochondrial function, suggesting its potential for IBD intervention. Chen *et al.* 293 evaluated the role of pelargonidin-3-galactoside (Pg3gal) from purple sweet potatoes on DSS-induced colonic inflammation in a murine model of UC. They observed that Pg3gal significantly attenuated DSS-induced UC, improved colon size, tissue condition, and inhibited proinflammatory cytokine production. It also modulated gut microbiota, reducing Proteobacteria and Deferribacteres while increasing Firmicutes, Bacteroidetes, and Verrucomicrobia.

On the other hand, **purple rice** is also a major source of anthocyanins, in particular C3G (Cyanidin-3-O-glucoside) and P3G (Peonidin-3-O-glucoside) 294. **Black rice** is also a major source of anthocyanins, although contains a lesser amount of polyphenols than purple rice 295,296. Thipart *et al.* 297 evaluated the effects of the anthocyanins from purple rice in acetic acid-induced UC and indomethacin-induced CD rats, showing that the microbiota was modulated in both cases. The relative abundances of beneficial bacteria, especially the Lachnospiraceae NK4A136 group and Lactobacillus, were decreased in the AA-induced UC model, whereas some opportunistic pathogens (Bacteroides, Escherichia/Shigella, Fusobacterium, and Veillonella) seemed to be raised by indomethacin-induced CD, suggesting that beneficial effects from this extract could be reported in UC models. Regarding black rice anthocyanins, one study 298 demonstrates that dietary black rice anthocyanin-rich extracts and rosmarinic acid could alleviate the symptoms and inflammation of DSS-induced colitis in mice by modulating MPO, NO, IL-6, IL-1β, TNF-α, iNOS and COX-2 levels, exerting more notable effects when used in combination.

Lastly**, Red cabbage** also represent a major source of anthocyanins, which are mainly derivatives of cyanidin-3-diglucoside-5-glucoside 299. In a study using C57BL/6J mice, the use of red cabbage juice (RCJ) significantly improved body weight, survival, and reduced DAI scores in DSS-induced colitis. RCJ enhanced colonic barrier integrity, increased SCFA-producing bacteria, activated PPAR-γ, and suppressed NFκB signaling, leading to reduced inflammatory cytokine production, indicating potential for IBD prevention and treatment 300. Overall, compelling evidence support the use of anthocyanins in the management of IBDs, although most studies have been performed *in vivo* and *in vitro*.

#### 2.1.7. Chalcones

Chalcones are phenolic compounds categorized as 'open-chain flavonoids' synthesized through the shikimate pathway, serving as precursors to flavonoids. Structurally, chalcones consist of two aromatic rings connected by an α,β-unsaturated ketone unit, with some variants known as dihydrochalcones featuring a saturated ketone instead 301. Natural chalcones commonly include phenolic hydroxyl groups and are frequently substituted with prenyl or geranyl moieties on the aromatic rings, with numerous examples documented in the literature. In the field of IBDs, some compounds like xanthohumol, isoliquiritigenin, cardamon in, or phloretin must be highlighted, with others like licochacone A, butein, flavokawain B or artepillin C showing some promising implications.

##### A) Xanthohumol

Xanthohumol (XN) is a prominent prenylated flavonoid present in the hop plant (*Humulus lupulus L*.) with many favorable effects in health and translational applications, acting as an anti-inflammatory, antimicrobial, antioxidant, immunomodulatory, antigenotoxic and antiangiogenic agent 302. XN can exert its benefits through different mechanisms, including the inhibition of the TLR4/MD-2 complex or the suppression of macrophage iNOS expression, NO, and IFN-γ production 303. This compound has also been shown to inhibit the TNF-α-activated NF-κB pathway *in vitro* and *in vivo* 304. Cho *et al.* 305 evaluated the use of xanthohumol in DSS-induced colitis mice. They observed that XN alleviated colitis symptoms, prevented colonic lesions, and inhibited pro-inflammatory cytokines, oxidative stress, and COX-2 expression. Besides, they show that this compound also suppressed the IKKβ/NF-κB signaling pathway, highlighting XN's potential as a therapeutic agent for colitis. In parallel, Yung *et al.* 306 examined the anti-fibrotic mechanism of XN on TGF-β1-induced intestinal fibrosis in human intestinal fibroblasts (HIFs). XN reduced fibrosis-related gene expression, restored altered cell shape, and inhibited both NF-κB and Smad signaling pathways. It interfered with TGF-Receptor I and Smad3 binding, highlighting the potential of XN in mitigating TGF-β1-induced fibrosis through TGF-β/Smad signaling inhibition. Restivo *et al.* 307 assessed the anti-inflammatory effects of a polyethoxylated flavone fraction (PMFF) from *Citrus sinensis* (particularly rich in nobiletin) and a prenylflavonoid fraction (PFF) from Humulus lupulus (with high levels of XN) both individually and combined (MIX), using an IL-1β-stimulated Caco-2 cell model of IBD. PMFF, PFF, and MIX reduced NO production, with MIX also inhibiting prostaglandin E2 release, NF-κB activation, and enhancing Nrf2 activation and antioxidant responses. Notably, the effects of MIX surpassed those of the individual fractions, highlighting the synergistic anti-inflammatory and anti-oxidative potential of nobiletin and XN. Magadán-Corpas *et al.* 308 assessed the effects of intraperitoneal injection of the flavonoids apigenin, luteolin, and XN in reducing inflammation and modulating gut microbiota in a murine model of ulcerative colitis. They observed that both luteolin and XN notably increased anti-inflammatory microorganisms and decreased pro-inflammatory species. All flavonoids reduced pro-inflammatory cytokines, with luteolin significantly alleviating physical UC symptoms. Apigenin showed limited microbiota impact due to solubility issues and accumulation in the mesentery.

##### Intervention studies in humans

To date, no studies have specifically evaluated the role of XN in humans with IBD. However, after a successful phase I trial confirming the safety and tolerability of 24 mg XN daily for 8 weeks his phase II clinical trial protocol aims to evaluate the safety and tolerability of 24 mg of xanthohumol (XN) daily, Langley *et al.* 309 are testing the safety and tolerability of the same dose in adults with active CD. This triple-masked, randomized, placebo-controlled study will involve up to 32 participants over 8 weeks. Outcomes will include adverse events, inflammatory biomarkers, platelet function, CD clinical activity, stool microbial composition, and XN metabolism. The study will compare these results with those from healthy adults in the phase I trial, showing the therapeutic potential of XN in CD and informing its broader clinical applications. Likewise, in a pilot study, Ryan *et al.* 310 aimed to identify the effects of a nutrition support formula on blood nutrient parameters in adults with IBD. The formula contained a mixture of micronutrients (including methylated forms of folate and vitamin B12), macronutrients, and phytonutrients (including curcumin, XN, ginger compounds, and quercetin). 10 participants with Crohn's disease or ulcerative colitis consumed a micronutrient and phytonutrient-rich beverage twice daily for 12 weeks. Significant increases in serum folate and decreases in red cell distribution width were observed. Modulation of leukocyte subtypes was noted, with a decrease in neutrophils and an increase in lymphocytes. Other parameters, including RBC count, hemoglobin, hematocrit, electrolytes, albumin, and inflammatory markers, did not change significantly. Therefore, XN can represent an interesting and potential flavonoid with some preliminary evidence in humans. However, more studies are warranted before drawing any relevant conclusion.

##### B) Isoliquiritigenin

Isoliquiritigenin (ISL) is a major bioactive chalcone compound isolated from the roots of plants belonging to licorice, including *Glycyrrhiza uralensis*, *Mongolian glycyrrhiza*, and *Glycyrrhiza glabra* 311. Among the many effects of ISL, the literature has described its ability to inhibit upstream of the NF-κB, NLRP3, and MAPK pathway, as well as to activate the Nrf2 pathway, thus exerting important anti-inflammatory and antioxidant effects 312. In DSS-induced UC mice, ISL ameliorated the reduction of body weight while improving colon length and structural integrity, diarrhea, bloody stool, DAI scores, and MPO activity 313. These effects were related to the suppression of the phosphorylation of ERK1/2 and p38 and the inactivation of NK-κB. Pretreatment with ISL significantly reduced indomethacin-induced intestinal damage and inhibited increases in cleaved caspase-1 and mature IL-1β protein levels 314. The protective effects of ISL were linked to its inhibition of NLRP3 inflammasome activation, as demonstrated by the lack of protective effect in NLRP3-/- and caspase-1-/- mice. ISL was shown to reduce the expression of the inflammatory markers IL-8, IL-1β, and COX-2, and inhibits NF-κB activation in TNF-α-stimulated HT-29 cells. Besides, it activates Nrf2 and its target genes and prevents the secretion of HMGB1 by reducing its acetylation through histone deacetylase (HDAC) activation 315. Collectively, these preclinical studies show that ISL is a potential agent for being explored in IBD. Also, meta-analysis and systematic reviews support the potential of licorice extract and its active compounds in UC preclinical studies, with potent anti-inflammatory, antioxidative, immunomodulatory, and microbiota-regulating effects as primary mechanisms of licorice extract and its compounds in treating UC 316. However, they acknowledges limitations including study quality variations, publication bias, and unexplored negative outcomes, warranting cautious interpretation and further clinical investigation.

##### C) Cardamonin

Cardamonin is a natural chalcone first discovered found in cardamom spice but it can also be found in various plants, exerting anti-inflammatory, antioxidant, antineoplastic, metabolic, and antimicrobial effects 317. Cardamonin demonstrated anti-inflammatory properties by reducing nitrous oxide production and downregulating iNOS, TNF-α, and IL-6 expression in RAW 264.7 cells without affecting cell viability while inhibiting NF-kB signaling. In a DSS-induced colitis mouse model, cardamonin protected against colitis symptoms, also showing therapeutic potential in colitis-associated cancer models 318. Likewise, cardamomin effectively inhibited necroptosis *in vitro* by blocking RIPK1/3 phosphorylation and disrupting necrosome formation in HT29, L929, and RAW264.7 cell lines, whereas *in vivo,* oral administration of cardamonin attenuates DSS-induced colitis in mice by mitigating intestinal barrier damage, suppressing necroinflammation, and reducing MLKL phosphorylation 319. Cardamonin effectively mitigated DSS-induced symptoms in mice, including body weight loss, diarrhea, colon shortening, and histological damage 320. This was associated with reduced inflammatory markers (MPO, NO, TNF-α, IL-6) in the colon, and inhibition of TLR-4 expression. Mechanistically, cardamonin blocked NF-κB and MAPK signaling pathways, including inhibition of NF-κB p65 nuclear translocation and downstream target gene expression, demonstrating its potential as a therapeutic agent for IBD. Oral and rectal administration of cardamonin significantly improved symptoms and histopathological changes in DSS- and TNBS-induced colitis in mice, as evidenced by reduced DAI scores, MPO activity, and colon length shortening 321. Cardamonin downregulated inflammatory markers (IL-1β, TNF-α, IL-6, NLRP3, cleaved caspase-1, ASC, cleaved IL-1β) in colonic tissues and inhibited NLRP3 inflammasome activation in THP-1 and bone marrow-derived macrophages. Mechanistically, cardamonin activated AhR, leading to enhanced AhR/ARNT complex formation, nuclear translocation, and XRE reporter gene activity. It also upregulated Nrf2 and its target genes, particularly NQO1, and these effects were attenuated by AhR antagonist CH223191 321. Rats treated orally with 10 or 30 mg/kg/day of cardamonin for 14 days before induction of UC with 3% AA showed decreased disease activity and macroscopic damage indices, along with significant histopathological improvement 322. Cardamonin also reduced levels of MPO, iNOS, NF-κB, TNFα, and MDA. Immunohistochemical analysis indicated decreased expression of COX-2 and caspase-3 in cardamonin-treated groups, suggesting its protective effects against AA-induced colitis by mitigating inflammation, oxidative stress, and apoptosis. Overall, these preclinical studies support the potential use of cardamonin in IBDs, although broader efforts in humans are still warranted.

##### D) Phloretin

Phloretin is a chalcone present abundantly in apples, pears, and strawberries acting as a potent antioxidant and modulating several signaling pathways and molecular mechanisms 323. Kapoor and Padwad 324 studied the many effects of this compound *in vitro* models of gut inflammation, developed by co-culture of Caco2 (intestinal epithelial) cells and RAW264.7 macrophages. They reported that phloretin reduced LPS-induced inflammation by lowering NO levels, oxidative stress, and mitochondrial membrane depolarization in Caco-2 cells, as indicated by decreased ROS and enhanced MMP. It also attenuated inflammatory cytokines (IL-8, TNF-α, IL-1β, IL-6) and inhibited NF-κB, iNOS, and Cox-2 expression. Phloretin maintained epithelial integrity by regulating tight junction proteins (ZO-1, occludin, Claudin-1, JAM) and reducing LPS-induced Cox-2 levels through modulation of Src expression. Additionally, phloretin combined with sodium pyruvate showed enhanced anti-inflammatory activity by targeting NF-κB signaling pathways. In a rat model of AA-induced colitis, phloretin, administered orally either before or after induction of colitis (50 mg/kg), effectively reduced plasma ALP and LDH levels, inflammatory markers (MPO, NO, eosinophil peroxidase), and colon ICAM-1 gene expression 325. It also restored tissue GSH levels, and prevented mucosal damage as confirmed by histopathological analysis, suggesting its potential as a natural therapeutic agent for UC management and future clinical applications. Phloretin (60 mg/kg) administered daily attenuated UC symptoms in mice induced by DSS, by reducing inflammation markers, preserving intestinal barrier integrity, and modulating systemic immune responses 326. This effect was attributed to the ability of phroletin to increase beneficial bacteria like Bacteroidetes, Alistipes, and Lactobacillus, while decreasing harmful Firmicutes, Oscillibacter, and Ruminiclostridium_6. Another study also found that this compound restored the disturbed faecal microbiota in DSS-induced mice and improved metabolic pathways by balancing fecal metabolites like norepinephrine, mesalazine, tyrosine, 5-acetyl-2,4-dimethyloxazole, and 6-acetyl-2,3-dihydro-2-(hydroxymethyl)-4(1H)-pyridinone 326. Correlation analysis revealed that various microorganisms were positively or inversely associated with these metabolites, evidencing the relevance of phloretin in the gut microbiota. Finally, phloretin administration reduced DSS-induced colitis by modulating NLRP3, TLR4, and PPARγ pathways, enhancing the expression of ZO-1 and occluding, and reducing serum LPS levels while restoring the balance of *Escherichia coli* and Lactobacillus in the gut 327.

##### E) Other chalcones

Other chalcones have also shown promising but less established therapeutic effects on IBD, as is the case of licochacone A, butein, flavokawain B, or artepillin C. **Licochalcone A (LA),** the predominant chalcone in *Glycyrrhiza inflata*, demonstrated significant protective effects against DSS-induced colitis in mice, reducing weight loss, DAI, histological damage, and gut inflammation 328. LA preserved intestinal barrier integrity by inhibiting apoptosis and maintaining tight junction protein expression. Dose-dependent effects were observed, with lower doses of LA primarily enhancing gut barrier integrity and higher doses focusing on anti-inflammatory actions. Additionally, LA modulated gut microbiota composition, particularly at lower doses, and exerted anti-UC effects partly through MAPK pathway inhibition, highlighting its potential therapeutic role in UC management 328. Likewise, LA seems to ameliorate DSS-induced UC by inhibiting NF-κB-regulated pro-inflammatory signaling and activating Nrf2-regulated cytoprotective protein expression 329. **Butein**, a major constituent of *Toxicodendron vernicifluum* ameliorated colitis in IL-10(-/-) mice by reducing the colonic inflammatory score by > 50%, reducing the expression levels of IL-6, IL-1β, IFN-γ pSTAT3 and MMP-9, also inhibiting IL-6-induced activation of STAT3 in Colo 205 cells 330. **Flavokawain B (FKB),** found in plants from the Zingiberaceae and Kava family, has equally demonstrated significant therapeutic effects in a DSS-induced IBD mouse model by reducing weight loss, restoring colon length, and mitigating inflammation 331. In this study, FKB targeted TLR2 to inhibit the formation of the TLR2-MyD88 complex, thereby suppressing the NF-κB signaling pathway both *in vivo* and *in vitro*. These findings suggest that FKB's anti-inflammatory properties involve direct modulation of TLR2, highlighting its potential as a therapeutic agent for IBD treatment. Finally, **artipilin C (ARC)** contained in Brazilian Green Propolis ameliorates UC and colitis-associated colorectal cancer by targeting p21-activated kinase 1 (PAK1) 332. The inhibition of PAK1 activation by ARC reduces NF-κB-mediated inflammation and enhances PPAR-γ activity, potentially maintaining intestinal integrity under inflammatory and neoplastic conditions.

Despite these studies, future research should focus on validating the effects through i*n vitro* and *in vivo* studies in murine and human models of UC, emphasizing the transition towards clinical trials. ***Table 1*** summarizes the effects of the main flavonoids explored in this section, highlighting the main dietary sources, mechanisms of action described in preclinical studies and clinical trials/studies in humans with doses used, if pertinent.

### 2.2. Phenolic acids

Their basic structure is based on having one carboxylic acid group. They are presented in amides, esters, or glycosides and in occasional situations are in free form 335. Their production is through the shikimic acid by the phenylpropanoid via, they can have chemistry structure possesses a C_6_-C_3_ structure (phenylpropanoid type), or a C_6_-C_1_ (phenylmethyl type), having an ancestor in their synthesis of lignins and other phenolics 336. Also, they are divided into three sub-groups: hydroxybenzoic and hydroxycinnamic acids 337. They are found to have an antioxidant effect, according to some studies, they have a range of activities that promote their antioxidant activity, it is suggested that the main pathway is radical scavenging through hydrogen atom donation.

#### 2.2.1. Hydroxybenzoic acids

Hydroxybenzoic acids have a common structure with benzoic acid in the structure C_6_-C_1_ 336. Flavonoids contribute to the action to promote IBD, by reducing inflammation and promoting mucosal barrier function, in DSS-induced colitis 338. The most important compounds are gallic acid, ellagic acid, protocatechuic acid, and vanillic acid.

##### A) Gallic and ellagic acid

On the one hand, **gallic acid (GA)** also known as 3,4,5-trihydroxy benzoic acid is found in plants, and fruits such as grapes, blackberries, strawberries, and raspberries 339. **Ellagic acid (EA)** is a condensed dimmer of gallic acid found in fruits, nuts, and seeds including pomegranates, raspberries, strawberries, walnuts, and almonds 340. According to past works 47 both GA and EA are able to ameliorate intestinal inflammation through several mechanisms, consequently ameliorating macroscopic colonic damage by mitigating edema, deep ulcerations, and hemorrhage.

In the case of **GA**, past works have shown that this compound downregulates the NLPR3 inflammasome protein and RNA expression in DSS-induced colitis in mice, improving the anti-inflammatory properties of this compound 341. It also is suggested to improve colitis by decreasing the deleterious metabolite ammonia while ameliorating gut microbiota dysbiosis 342. More specifically, GA can raise the proportion of probiotic bacteria like Lactobacillaceae and Prevotellaceae, while reducing some pathogenic species, mainly in the Firmicutes and Proteobacteria phyla 343. These microbial variations are also related to metabolic changes including augmented carbohydrate and bile acid metabolism, decreasing amino acid metabolism. In parallel, Leng *et al.* 344 also reported that the use of GA altered the diversity of the gut microbiota and activated the bile acid metabolic pathway, being this fact associated with enhanced ILC3 cells in mesenteric lymph nodes and lamina propria accompanied by reductions in TNF-α, IFN-γ, IL-6, IL-17A, and IL-23, and elevations in IL-10, TGF-β and IL-22. GA also stimulates the expressions of TJ proteins, ameliorates cell apoptosis, and oxidative stress, and suppresses the activation of the NF-κB/MAPK pathway to alleviate LPS-induced intestinal inflammation in Caco-2 cells 345. Similar results and conclusions were obtained in TNBS-induced UC in mice 346. In DSS-mice, GA also exerted anti-inflammatory mechanisms by suppressing the IL-6/p-STAT3 (Y705) and p65-NF-κB activation 347.

Regarding EA, *in vitro* studies in caco 2 cells show that it acts through NF-κB and ERK1/2 inhibition, breaking the cycle of inflammation, oxidative stress, redox-sensitive pathway activation, and intestinal permeabilization 348. Moreover, a diet supplemented with EA mitigates oxidative stress during colitis by bolstering Nrf2 signaling pathways in piglets treated with paraquat, protecting against intestinal injury by facilitating the maintenance of tight junction structure and intestinal barrier integrity, while also preserving jejunal and ileal morphology, including villus height, goblet cell number, and the ratio of villus height to crypt depth 349. Similar observations were made in TNBS-induced colitis mice, as EA acted by maintaining mucin secretion and intestinal barrier function 350. The anti-inflammatory effects of this compound have also been observed in acute and chronic models of DSS-induced UC mice. In acute models, a dietary supplement of EA (2%) led to reductions in IL-6, TNF-α, and IFN-γ, whereas in the chronic UC model, EA significantly inhibited the progression of the disease, reducing intestinal inflammation and decreasing histological scores, mainly through the downregulation of COX-2 and iNOS and the blockage of signaling pathways like p38 MAPK, NF-κB, and STAT3 351. Similar conclusions were drawn for animal models of CD 352,353. Like GA, EA also seems to have important modulatory effects on gut microbiota. Co-treatment with a relevant dose (60 mg/kg/day) of EA for 7 days significantly reduced DSS-induced gut barrier dysfunction, endotoxemia, and inflammatory injuries in the gut, liver, and brain in mice. This was achieved by modulating gut microbiota composition (reducing Bacteroides and *E. coli* and increasing the abundance of Lactobacillus) and inhibiting elevated oxidative and nitrative stress markers 354. Likewise, EA also shows a favorable effect on gut microbiota and immune response in piglets treated with paraquat 355, showing the multiple potential benefits associated with this molecule.

##### B) Protocatechuic acid and vanillic acid

**Protocatechuic acid (PCA)** is a member of the hydroxybenzoic acids, it is also known as 3,4-dyhyrdroxybenzoic acid found in medical herbs, vegetables, and various plants such as olives, roselle, du-zhong, calamondin, and white wine grapes 356. Yang *et al.* 357 showed that PCA reduced the levels of the disease activity index, inflammatory factors, and histological damage in UC mice, mainly through the modulation of Bacteroidetes. Moreover, PCA seemed to downregulate the level of ferroptosis in the colon tissue, evidenced by a reduced iron overload, decreased GSH depletion, and a lower level of MDA production, whereas similar results were observed in Erastin-treated Caco-2 cells. PCA can also lead to reduced levels of IL-1β, IL-6, and TNF-α, also ameliorating DSS-induced ZO-1 and claudin-2/4 redistribution 358. Crespo *et al.* suggested that PCA could modulate the sphingosine kinase (SphK)/S1P system and related signaling pathways to exert its anti-inflammatory effects in TNBS models of IBD 359. They observed that PCA administration effectively prevented colonic damage, weight loss, and MPO activity increase induced by TNBS, while also modulating antioxidant enzyme expression, proinflammatory cytokines, and signaling molecules such as AKT, ERK, pSTAT3, and NF-κB p65. In another study, PCA administration significantly prevented colitis symptoms, reduced pro-inflammatory cytokines and liver toxicity markers, and protected against oxidative damage in both the colon and liver, highlighting its chemoprotective role 360.

**Vanillic acid (VA)** is a natural benzoic acid derivative commonly found in herbs, rice, maize, and edible plants and fruits 361. Ni *et al.* demonstrated the relevance of the use of VA in treating DSS-induced colitis by restoring intestinal epithelium homeostasis through the inhibition of ferroptosis. They reported that VA acted by direct targeting of carbonic anhydrase IX (CAIX, CA9), leading to the activation of insulin-induced gene-2 (INSIG2) and subsequent interaction with stromal interaction molecule 1 (STIM1), resulting in the translocation of SCAP-SREBP1 and the upregulation of stearoyl-CoA desaturase 1 (SCD1) transcription. This cascade effectively inhibits ferroptosis-mediated excessive death of intestinal epithelial cells, preserving intestinal barrier integrity and mitigating unresolved inflammation. On the other hand, Kim *et al.* 362 showed that VA reduced clinical manifestations of colitis such as weight loss, colon length shortening, and disease activity index. Moreover, VA suppressed the expression of COX-2 and the activation of transcription nuclear factor-κB p65 in DSS-treated colon tissue, also decreasing plasma levels of IL-6 suggesting its efficacy in regulating chronic intestinal inflammation.

#### 2.2.2. Hydroxycinnamic acids

These form part of this group due to their chemical structure in this case has a cinnamic acid structure with one or more hydroxyl groups linked with the phenyl ring, in different from hydroxybenzoic acids, that this last one does not have a cinnamic structure. They are found in fruits, vegetables, grains, coffee, and tea 363. Some common hydroxycinnamic acids are chlorogenic acid, caffeic acid, ferulic acid, sinapic acid, rosmarinic acid, coumarinic acid, and quinic acid.

##### A) Chlorogenic and caffeic acid

Chlorogenic acid and caffeic acid are the two critical phenolic acids found in coffee, representing along with caffeine some of the major bioactive compounds in this drink 364. **Caffeic acid** is also found in various plant-based foods, including fruits and vegetables, and drinks like tea or wine. It consists of a cinnamic acid moiety with a hydroxyl group substituted at the 3-position on the phenyl ring. In RAW 264.7 cells and colon epithelial cells, HT-29 is found that caffeic acid acts as an anti-inflammatory modulator by suppressing the production of nitric oxide (NO), IL-1β, IL-6, IL-8, and TNF-α, suggesting their potential therapeutic in IBD 365. Xiang *et al.* 366 demonstrated that caffeic acid could attenuate DSS-induced murine UC by interfering with the activation of macrophages, thus representing an alternative therapeutic option for this condition. Other works have also demonstrated that caffeic acid could exert its benefits in these animals by increasing the Akkermansia population 367. One derivated of caffeic acid is FA-97 and it is synthetic and is found to reduce DSS-induced colitis against oxidative stress by the activation of the Nrf2/HO-1 pathway 368. In parallel, caffeic acid phenyl ester is a bioactive compound of propolis extract with proven anti-inflammatory actions. Among their protective and therapeutic effects against IBDs demonstrated *in vitro* and in DSS-induced UC mice models, the studies have shown that this compound is able to ameliorate NLRP3 inflammasome activity 369, by reducing myeloperoxidases and proinflammatory cytokines 370 and also through modulating NF-kB activation while reducing intercellular adhesion molecules (ICAM)-1 and vascular cell adhesion molecules (VCAM) 371.

On the other hand**, chlorogenic acid** is also found in a plethora of plant sources like apples, artichoke, betel, burdock, carrots, eggplants, grapes, kiwi, potatoes, tea or tomatoes, among others 372. Chemically, it comprises a caffeic acid moiety and a quinic acid moiety; hence, it is also referred to as 5-O-caffeoylquinic acid. Gao *et al.* 373 claimed that chlorogenic acid is able to reduce DSS-induced colonic mucosal damage, by alleviating DSS-induced inflammation, oxidative stress, and apoptosis in the colon, while reducing ERK1/2, p -ERK, p38, p-p38, JNK, and p-JNK protein expression. Similar findings were made by Vukelić *et al.* 374, who also observed that chlorogenic acid suppressed the activation of pro-inflammatory and apoptotic signaling pathways in these animals. Zeng *et al.* 375 showed in these animal models and in RAW264.7 cells that chlorogenic acid prevented colitis by downregulating miR-155 expression and inactivating the NF-κB/NLRP3 inflammasome pathway in macrophages. Maslin *et al.* 376 observed that despite the addition of quercetin and chlorogenic acid to a DSS-induced UC mice did not protect against indicators of injury and inflammation, or fecal SCFA concentrations compared to the control diet, these compounds influenced the expression of various injuries repair molecules, pro-inflammatory cytokines, SCFA transport proteins, and NF-κB inhibitory molecules. Thus, they suggested that these compounds exert their maximum benefits in healthy individuals or during periods of remission, whereas they may suppress some of the signaling involved in inflammation promotion during active disease stages 376. Among the main benefits of chlorogenic acid in the gut microbiota in DSS-mice, is a marked decrease in the proportion of Firmicutes and Bacteroidetes, and microbial diversity, along with a marked increase in Akkermansia has been reported by Zhang *et al.* 377. Similar observations were made for IL-10 knockout mice models of IBD, as chlorogenic acid was able to reduce the expression levels of iNOS, IL-1β, and TNF-α 378, whereas chlorogenic acid was able to inhibit the growth of *Bacteroides* and the accumulation of *Bacteroides*-derived LPS, in indomethacin-induced colitis 379.

##### B) Ferulic and sinapic acid

**Ferulic acid** has a similar structure as caffeic acid, but it contains an additional methoxy group linked to the phenyl ring. It is presented in oranges, apples, and tomatoes 380. It is suggested to have a protective action in the intestinal tight junctions, and also suppresses the endoplasmic reticulum (ER) stress, NO generation, and inflammation in Caco-2 and T84 cells, demonstrating its anti-inflammatory and anti-oxidant activities 381. *In vivo*, the relevance of ferulic acid has been demonstrated in multiple animal models using 2,4,6-trinitrobenzene sulfonic acid, intrarectal acetic acid, DSS and TNBS-induced UC 382-385. In this sense, Yu *et al.* 385 show that ferulic acid can alleviate intestinal injury in UC rats and inhibit inflammatory factor levels (IL-6, IL-12, and IL-1β), apoptosis-related protein expression (caspase-1 and caspase-3) and the TXNIP/NLRP3 signaling pathway. Besides, greater effects were observed for those animals identified in the middle and high intervention group (20 mg/kg of ferulic acid per day during 14 days and 250 mg/kg, respectively) when compared to the low intervention group (10 mg/kg per day). Simultaneously, Ghasemi-Dehnoo *et al.* 383 showed that ferulic acid administered orally one hour after the UC induction and during five days at 20, 40, and 60 mg/kg doses ameliorated UC through the inhibition of the LPS-TLR4-NF-κB and NF-κB-INOS-NO signaling pathways. Importantly it seems that higher doses were also associated with broader improvements. Sadar *et al.* 382 also reported that ferulic acid could ameliorate TNBS-induced colitis by inhibiting IFN-γ induced inflammatory cascade, driving a concomitant decrease in the release of pro-inflammatory cytokines. Likewise, a greater concentration of the compound (40mg/kg once per day during 14 days) drove to greater improvements when compared to middle doses (20mg/kg day) and low doses (10 mg/day) A ferulic acid derivate designed as C1a (ferulic acid conjugated with octopamine) was shown to effectively alleviates clinical signs and inflammatory mediators *in vitro* and *in vivo* models of IBD, showing even greater benefits that ferulic acid alone at the same doses (1 or 5 mg/kg per day during 14 days) 384.

**Sinapic acid (SA)** forms part of the hydroxycinnamic acids by their similarity in their structure, which has in common with ferulic acid, but in this case, it also has a vinyl group attached to the phenyl ring. It is found in various edible foods like berries, citric fruits, oil seeds, wheat, rice, spices, and plants like *Salvia officinalis* and *Myristica fragrans* 386. Jang *et al.* 387 observed that SA was able to mitigate stimulus-induced delocalization of tight junction proteins, reduce intestinal permeability, and suppress inflammatory cytokine expression both *in vitro* and *in vivo*. SA is directly bound to transforming growth factor beta-activated kinase 1 (TAK1), inhibiting NF-κB and MAPK/ATF-2 pathways, thereby regulating mitogen-activated protein kinase (MLCK) expression. Dietary sinapic acid also improved gut microbiota imbalance and IBD symptoms, suggesting its potential as a nutraceutical and pharmaceutical agent for IBD treatment by targeting TAK1 and inhibiting subsequent NF-κB and ATF-2 signaling. Pretreatment with SA (40 mg/kg/day) significantly mitigated colonic injuries and acetic acid-induced UC symptoms in rats, reducing oxidative stress (MDA, NO levels), restoring antioxidant/oxidant balance (catalase, glutathione levels), suppressing inflammation (TNF-α, IL-6, MPO, PGE2, COX-2, NF-κB), and inhibiting apoptosis (Bax, caspase-3), while increasing anti-apoptotic protein Bcl-2 expression 388. Oral administration of SA (10, 30, and 100 mg/kg) to female Balb/c mice post-TNBS instillation significantly improved colonic weight and length, reduced macroscopic and microscopic colonic changes, and lowered myeloperoxidase activity, malondialdehyde levels, and tumor necrosis factor-alpha expression compared to TNBS control 389. Li *et al.* 390 reported that SA supplementation could significantly improve clinical symptoms, reduce pathological changes, repair intestinal mucosal barrier function, and maintain epithelial homeostasis by inhibiting NLRP3 inflammasome activation and decreasing pro-inflammatory cytokine expression. SA also increased antioxidant enzyme expression via the Nrf2/keap1 pathway, enhanced autophagy through the AMPK-Akt/mTOR signaling pathway, and reduced intestinal fibrosis-associated proteins Collagen-I and α-SMA. Similarly, Qian *et al.* 391 observed that SA administration attenuated oxidative damage by enhancing SOD, GSH, and CAT activity while decreasing serum and colonic mRNA levels of proinflammatory cytokines. Mechanistically, SA reduced NLRP3 inflammasome activation and enhanced intestinal barrier integrity by upregulating ZO-1, occludin, and claudin-1 expression. *In vitro*, SA also influenced cell viability, decreasing epithelial permeability and restoring the protein and mRNA expression of claudin 1, and ZO-1 in LPS-treated Caco-2 cells 392.

##### C) Rosmarinic, coumaric and quinic acid

**Rosmarinic acid (RA)** is a type of polyphenol belonging to the class of phenolic acids. It is specifically an ester of hydroxycinnamic acid, found in various plants, including rosemary, basil, and mint, among others. RA has been studied for its potential antioxidant, anti-inflammatory, and antimicrobial properties, making it an important component of many herbs and plant-derived foods 393. In the event of IBDs, RA has proven its efficacy in alleviating intestinal inflammation, ameliorating tight junction damage, gut dysbiosis, endoplasmic reticulum stress, cell death, and smooth muscle contractile dysregulation 394. Formiga *et al.* 395 observed that RA had an important anti-inflammatory activity in the gut through cytoprotection, mucosal barrier maintenance, and modulation of antioxidant and immunomodulatory systems. Indeed, they observed that oral administration of RA (25-200 mg/kg) reduced macroscopic lesions, ulcerative area, intestinal weight/length ratio, and diarrheal index. At higher doses (200 mg/kg), RA decreased MDA and MPO, restored GSH levels, enhanced SOD activity, reduced IL-1β and TNF-α levels, and also modulated T cell populations, reducing mRNA transcription of inflammatory markers, and enhanced gene expression and positive cell staining for MUC-2 and ZO-1. In a mouse model of DSS-induced colitis, RA significantly reduced colitis severity, as evidenced by decreased disease activity index scores, colonic damage, and colon length. RA also lowered levels of inflammatory cytokines (IL-6, IL-1β, IL-22) and protein expression of COX-2 and iNOS, inhibiting NF-kappa B and STAT3 activation and subsequently reducing pro-survival gene activity dependent on these transcription factors 396. Similarly, the use of chitosan/nutriose-coated niosomes to increase RA local bioavailability led to significant improvements in acute colitis through the downregulation of the protein expression of inflammasome components such as NLR family pyrin domain-containing 3 (NLRP3), adaptor protein (ASC) and caspase-1, and the consequent reduction of IL-1β levels 397. Also, this nanovesicle drove a significant increase in the expression of Nrf2 and HO-1.

**Coumaric acid** is a phenolic acid from the hydroxycinnamic acid family synthesized through the shikimate pathway with phenylalanine and tyrosine as precursors 398. It is abundantly found in fruits (apples, pears, grapes, oranges, tomatoes, and berries), vegetables (e.g. beans, potatoes, and onions), cereals (e.g. maize, oats, and wheat), mushrooms, and medicinal herbs 399. **Quinic acids** are polyphenol esters formed of hydroxycinnamic acids and quinic acid mainly found in plants (Yerba mate, white, green teas, and coffee), microalgae, and cyanobacteria 400. One study conducted in 64 male Wistar rats evaluated the use of quinic acid in treating UC induced by acetic acid in rats. This acid, especially at higher doses, demonstrated promising therapeutic potential for UC by significantly increasing the expression of HO-1, Nrf2, and NQO1 mRNA, while decreasing tissue levels of TNF-α and IL-1β protein in colon tissue 401. Regarding quinic acid, one study 402 showed that this compound attenuated UC by inhibiting the TLR4-NF-κB and NF-κB-iNOS-NO signaling pathways, thereby reducing colitis-related complications such as oxidative stress, inflammation, apoptosis, and histopathological damage in the same animal models. ***Table 2*** summarizes the effects of the main phenolic acids explored in this section.

### 2.3. Stilbenes

They are considered phytochemicals, some of them, also are included as phytoalexins, and their chemical composition is formed by a 1,2-diphenylethylene backbone. They are found in berries, grapes, peanuts, and red wine 403. The three most important stilbenes are, resveratrol, piceatannol and pterostilbene.

#### A) Resveratrol

**Resveratrol (RES)** also known as 3,4,5-trihydroxystilbene is presented mainly in wine and grapes 404. RES is a potent antioxidant and anti-inflammatory molecule widely explored in the management of immunoinflammatory diseases like IBD 405. The mechanism of action of RES involves multiple immune responses and signaling pathways. RES is absorbed quickly and metabolized into various derivatives. However, the poor water solubility of this molecule and its low bioavailability limit its clinical applications 406. RES also presents a bidirectional relationship with gut microbiota, showing modulatory effects on the microbial community, which in turn influence its metabolism and action 407. More specifically, *in vitro* studies have shown that RES reduces inflammation in UC by decreasing IL-1β levels and increasing IL-11 production, primarily through the modulation of the Nrf2 pathway in both TNF-α challenged Caco-2 cells and patient tissue samples 408. RES and 3-(4-hydroxyphenyl)-propionic acid (4HPP), a microbial metabolite of RES, significantly reduced paracellular permeability and proinflammatory cytokine secretion in LPS-treated Caco-2 cells, correlating with increased TJ protein expression 409. Both compounds ameliorated intestinal barrier dysfunction and colonic inflammation in colitis mice, mediated through AMPK activation and regulation of the SIRT1/NF-κB pathway, whereas dihydro resveratrol (DHR), another microbial metabolite of RES, showed no significant effects either *in vivo* or *in vitro*. RES was equally able to reverse the LPS-induced downregulation of occludin, ZO-1, and claudin-1 in HT-29 cells as well as attenuating the Notch1 pathway and subsequently reducing IL-6 and TNF-α levels 410.

Serra *et al.* 411 compared the role of cyanidin-3-glucoside and RES with 5-aminosalicylic acid (5-ASA) in HT-29 intestinal cells. The results showed that both polyphenols, at lower concentrations than 5-ASA, activated Nrf2, increased HO1 and glutamate cysteine ligase mRNA expression, enhanced the reduced-to-oxidized glutathione ratio, and inhibited reactive species production, with resveratrol and 5-ASA also increasing nuclear PPAR-γ levels. They concluded that these compounds could act as complementary nutraceuticals in managing intestinal inflammation in inflammatory bowel disease. The stimulation of the Nrf2/HO‑1 pathway by RES reduced clinical symptoms, inflammatory responses, and intestinal mucosal barrier damage in experimental UC mice as well 412. On the other hand, Garcia *et al.* 413 demonstrated the antifibrotic effects of RES on intestinal smooth muscle cells from a CD rat model, finding that this compound decreased cell numbers through cell cycle arrest and apoptosis, and reduced collagen synthesis and procollagen mRNA expression. Similar conclusions were obtained in the PG-PS rat model of CD, as the use of RES significantly reduced inflammatory cytokines and TGF-β1 and demonstrated a promising trend in decreasing tissue fibrosis 414. Resveratrol also enhances xenophagy, promoting autophagy-dependent clearance of intracellular bacteria *in vitro* (in intestinal epithelial cells and macrophages) and *in vivo* (transgenic GFP-LC3 zebrafish), suggesting potential for developing pro-autophagic nutrients to maintain intestinal homeostasis and combat infections 415.

Multiple animal models have also validated the relevance of RES in the management of IBD through several additional mechanisms. These actions included down-regulation of Wnt signaling pathway 416, regulation of gut microbiota diversity 417, regulation of arginine metabolism in macrophages 418, modulation of SUMO1 through the Wnt/β-catenin pathway 419, inhibition of STAT3 O-GlcNAcylation and reduction JAK2/STAT3 pathway activity 420, suppression of the intestinal inflammatory cascade reaction, and regulation of autophagy and SIRT1/mTOR signaling 421,422, modulation of the PI3K/Akt/VEGFA pathway 423, reduction of neutrophil infiltration, inhibition of adhesive molecules, restoration of the NO and redox status 424,425, recovery of the Treg/Th17 balance and the HIF-1α/mTOR signaling pathway 426, induction of immunosuppressive CD11b(+) Gr-1(+) cells 427 promotion of MUC2 synthesis via the ANRIL-miR-34a axis 427, NLRP-3 inflammasome repression 428, AMPK-mediated activation of CDX2 and the regulation of the SIRT1/NF-κB pathway 429, downregulation of miR-31 430,431, inhibition of SphK1 , and downregulation of the p38 MAPK 432. Overall, *in vivo* and *in vitro* studies show that RES can have important effects in alleviating IBD, modulating a plethora of mechanisms associated with this chronic condition.

#### Intervention studies in humans

Human studies have demonstrated significant benefits from resveratrol supplementation in people with UC. One work 433 analyzed the effects of the Mediterranean diet (MD) combined with curcumin and resveratrol supplementation on disease activity, serum inflammatory markers, and quality of life in patients with mild to moderate UC. In this multicenter three-arm randomized controlled trial, participants were randomized into the MD, MD + curcumin, and MD + resveratrol groups. All groups followed the MD for 8 weeks, with the addition of either curcumin (1600 mg/day) or resveratrol (500 mg/day) supplementation for the respective groups. Results demonstrated that all interventions effectively reduced disease activity and inflammation while improving the quality of life in UC patients. Similar results were obtained in a randomized, double-blind, placebo-controlled study 434, in which 50 patients with active mild to moderate UC were given either a 500-mg resveratrol or placebo capsule for 6 weeks. The resveratrol group showed significant reductions in serum inflammatory markers, including TNF-α, c reactive protein (CRP) and NF-κB activity in PBMCs with no significant changes in the placebo group. Quality of life scores (IBDQ-9) increased and clinical colitis activity index scores decreased significantly in the resveratrol group compared to the placebo group. Likewise, 500 mg/day resveratrol capsules or the same amount of placebo for 6 weeks given to 56 patients with mild to moderate UC also reported that the intervention group show an increase in total antioxidant capacity, serum levels of SOD, and the quality of life, along with a decrease in MDA levels and disease activity 435.

Overall, these studies support that 500 mg resveratrol supplementation can improve quality of life and reduce disease activity in UC patients, especially in combination with a healthy diet such as MD. However, the long-term effects of resveratrol in these patients require further investigation.

#### B) Piceatannol

**Piceatannol** (PIC) is a natural compound found in various plants, including grapes, peanuts, and blueberries. It is structurally similar to resveratrol and has gained attention due to its potential health benefits. For instance, past works have demonstrated that PIC is able to reduce several proinflammatory mediators in activated immune cells and induce regulatory T cells *in vitro* while modulating adipocyte function 436. PIC was shown to repress inflammation, inhibit cell apoptosis, and regulate microbiota composition, with some synergic effects when combined with 3'-hydroxy pterostilbene 437. Both compounds seemed to increased representative probiotic species, including Akkermansiaceae and *Lactobacillus intestinalis*, while exerting inhibitory effects on several bacterial species (Spiroplasmataceae and Acholeplasmataceae). Another study showed that oral administration of resveratrol or PIC (10 mg/kg body weight each) for 7 constitutive days attenuated the DSS-induced inflammatory injury, upregulation of iNOS expression, and activation of NF-kappaB, STAT3, and ERK 438. Likewise, PIC ameliorated the disruption of the colonic architecture, along with a significant reduction in colonic myeloperoxidase (MPO) activity, and a decrease in the production of inflammatory mediators such as nitric oxide (NO), prostaglandin (PGE2, as well as various pro-inflammatory cytokines 439.

#### C) Pterostillbene

**Pterostilbene (PTS)** (3,5-dimethoxy-4-hydroxystilbene), is an analogue of resveratrol that has the same health-promoting properties as resveratrol 440. This compound is mainly found in blueberries and *Pterocarpus marsupium* heartwood 441. In DSS-induced mouse colitis models, PTS is shown to inhibit the activity of NLRP3 inflammasome, demonstrating its anti-inflammatory properties 442. In UC mice induced by high-fat diet (HFD) and DSS, the use of PTS significantly reduced inflammation, aberrant crypt foci formation, and colon weight-to-length ratio, by ameliorating IL-1β, C/EBP homologous protein (CHOP), COX-2, and TGF-β1 while maintaining mucin2 and E-cadherin expressions 443. PTS can also inhibit DC-mediated T cell proliferation, reducing Th1 and Th17 populations, and increasing Treg populations. Likewise, this compound suppressed DC-induced inflammatory cytokine production by attenuating the transcription factor PU.1 and promoting Foxp3+ Treg differentiation, effectively alleviating symptoms of DSS-induced colitis and decreasing TNF-α expression in mice 444. ***Table 3*** summarizes the effects of the main stilbenes explored in this section.

### 2.4. Lignans

This group is formed by two phenylpropane motives united by a C6-C3 bond. They are found in plants such as the latter, flaxseed, and seams seed, and also can be found in fish, meat, oilseed, and beverages 446.

Firstly, it should be highlighted the multiple studies conducted with flaxseed and flaxseed oils, particularly rich in lignans. Indeed, flaxseeds represent the first plant and food with the highest content of lignans, with approximately 301,000 μg of lignans /100 g 447, at present the maximal known content of any foodstuff. The major lignan in flaxseed is called secoisolariciresinol diglucoside (SDG) 448. Because of this, a significant number of preclinical studies support the relevance of lignans in the treatment of IBDs, whereas some clinical trials have also been conducted in this sense. However, additional lignans present in sesame and other sources should also be highlighted. In this section, these groups of lignans will be summarized.

#### A) Flaxseed and secoisolariciresinol diglucoside

Some *in vivo* models have found that flaxseed oil supplementation had a beneficial health effect in a physically active mouse model of CD susceptibility, leading to favorable changes in the gut microbiota 449. Aqueous-methanolic crude extracts of Flaxseed (Fs.Cr) and Flaxseed oil were evaluated for their therapeutic effects on AA-induced colitis in mice, with microscopic analysis of colon tissue and assessments of antispasmodic and antibacterial activities 450.

Flaxseed oil reduced mortality and colonic ulcers more effectively, while Fs.Cr increased mucin content, exhibited stronger anti-inflammatory effects, and demonstrated significant antispasmodic and antibacterial activities in these animals. Fs.Cr was found to regulate several additional key molecules involved in IBD, reducing the levels of TNF-α, IFN-γ, MDA and MPO, and increasing IL-17 SOD, GPX, CAT, and total GSH, thus demonstrating their role in decreasing UC severity by reducing oxidative damage, inflammation, and promoting mucosal repair 451. Silva *et al.* 452 explored the fractions containing phenolic compound isolate (Phi) and phenolic reduced-flaxseed protein hydrolysate (phr-FPH) from flaxseed in TNBS-induced UC mice. They observed that both Phi and phr-FPH reduced levels of TNF-α and NO in stimulated macrophages. Moreover, Phi and phr-FPH treatments prevented weight loss and colon inflammation in colitic BALB/c mice, also decreasing T cell proliferation, Th1 and Th17 cells, and pro-inflammatory cytokines, while increasing Treg cells in spleen cell cultures from Phi-treated mice. SDG also showed promising effects in the DSS-induced colitis model and LPS-stimulated RAW264.7 macrophages. In more detail, SDG supplementation attenuated colitis severity, reduced macrophage infiltration, and lowered inflammatory cytokine levels while inhibiting NLRP1 inflammasome activation, partly through NF-κB disruption, suggesting its potential as an IBD treatment 453. Lignans and flaxseed oligosaccharides and proteins seem to benefit significantly in animal models of IBD 454,455. Conversely, another work 456 found that neither the administration of extra virgin olive oil (EVOO) nor flaxseed oil alone or in combination had significant benefits for preventing or ameliorating any symptoms in DSS-induced acute UC mice. Indeed, their use was associated with slight adverse effects such as increased spleen weight-to-body weight ratio and inflammatory markers. In a similar line, ground flaxseed supplementation seemed to ameliorate the benefits from a reduced-fat diet in a *Citrobacter rodentium*-induced model of colitis 457.

#### Intervention studies in humans

Interestingly some studies in humans evaluating the role of flaxseed supplementation have been conducted by Morshedzadeh *et al.* 458-461. In a first open-labeled randomized controlled trial with 75 UC patients, Morshedzadeh *et al.* 459 randomly assigned subjects into one of the following groups for 12 weeks: group 1) receiving 30 g/day of grounded flaxseed (GF); group 2) receiving 10 g/day of flaxseed oil (FO) and group 3) receiving placebo. They observed that both flaxseed and flaxseed oil, attenuate inflammatory markers, disease severity, blood pressure, and waist circumference when compared to the placebo. In more detail, a significant decrease was observed in fecal calprotectin, erythrocyte sedimentation rate, INF-γ, IL-6, waist circumference, diastolic blood pressure, and systolic blood pressure, and a significant increase was noted in TGF-β and Inflammatory Bowel Disease Questionnaire-Short form (IBDQ-9) score in the GF and FO groups compared to the control. No significant difference was observed between the FO and GF groups except for TGF-β. Similarly, they showed significant reductions in hs-CRP and Mayo score, alongside increased quality of life in both GF and FO groups compared to the control. FO showed a significant increase in IL-10 concentration but no significant changes were observed in TLR4 between intervention and control groups 458. Besides, the severity of the disease positively correlated with the decrease in Mayo score. When they compared the effects on metabolic syndrome parameters of Flaxseed supplementation (30 g/day GF) with placebo in 70 patients with mild to moderate UC, reduced insulin, HOMA-IR, triglyceride, and total cholesterol levels were observed along with increasing HDL levels 460. Improvements were also noted in SCCAI score and TNF-α although no significant differences were observed in body weight, BMI, waist circumferences, or blood pressure between the intervention and control groups. In another trial, 64 with mild and moderate Flaxseed supplementation (30g/day of GF) resulted in a significant reduction in resistin and visfatin concentration and a significant increase in adiponectin levels 461. Therefore, flaxseed supplementation (30 g/day GF or 10g/day FO) during 12 weeks seemed to have positive effects on patients with mild to moderate UC, improving a broad spectrum of inflammatory, metabolic, and clinical markers. However, additional long-term studies are required before drawing any definitive conclusion.

#### B) Sesame seeds, sesamin and sesamol

Sesame seeds exhibit the second-highest lignan concentration, with a total of 39,348μg/100g 462. Major lignans of sesame are sesamin and sesamolin, although other important members include sesamol, sesaminol, its epimers, and episesamin 463. The use of sesame oil has demonstrated significant benefits in the treatment of IBD in preclinical models. For instance, sesame oil can decrease the levels of MPO 464, whereas other studies have found that 4 mL/kg for 7 days of this product can ameliorate TNBS-induced UC in rats by reducing the degree of inflammation, fibrosis, and acidic mucin while increasing neutral mucin 465. On the other hand, sesame cake, a by-product of sesame oil production, improved symptoms of DSS-induced colitis, ameliorated histopathological damage of the mucus layer in colon tissues, and decreased pro-inflammatory cytokines apoptosis and oxidative stress in colitis-induced colon tissues 466. Likewise, the use of Kanjangs (Fermented Soy Sauce and Sesame Sauce) exerted an anticolitic effect partially by reducing the serum levels of proinflammatory cytokines and inhibiting the mRNA expression of these factors in the colon tissue of DSS-induced C57BL/6 mice of UC 467. Interestingly, low-dose treatment (4 mL/kg) with the fermented sauces resulted in broader anticolitic effects than consumption of a higher quantity (8 mL/kg).

Regarding specific lignans from sesame, **sesamin (SES)** exerts a protective action against UC by activating AKT/ERK and the increasing signaling of Nrf2 468. Likewise, SES treatment reduced the DAI values and improved the histopathology of the colon in the DSS-treated mice, diminishing TNF-α, IL-1β, and IL-6 production caused by DSS through the modulation of the NF-κB and MAPK signaling pathways 469. **Sesamol** has also obtained some interesting results in this field. Zhao *et al.* 470 demonstrated that sesamol treatment (100 mg/kg per day) for 6 weeks inhibited the DSS-induced bodyweight loss of mice, along with the DSS-induced histopathological changes and inflammatory responses by targeting the NF-κB signaling pathway in mice colon. Moreover, they showed that sesamol prevented gut barrier damage by enhancing the expression of tight junction proteins (occludin, claudin-1, and ZO-1), recovering the loss of gut mucus layer while increasing SCFAs through changes in the gut microbiome structure by enhancing the relative abundance of Coprococcuscus, Butyricicoccus, Odoribacter, and AF12 470. In a similar experimental design, Xia *et al.* 471 observed that sesamol treatment (100 mg/kg per day) for 6 weeks prevented inflammatory response, epithelial barrier dysfunction, and depression-like and anxiety-like behaviors in DSS-induced UC mice via gu the-brain axis. In more detail, sesamol alleviated neuroinflammatory reactions by suppressing the TLR-4/NF-κB pathway, protected against oxidative stress, and upregulated the Nrf2 antioxidant signaling pathway, improving brain-derived neurotrophic factor (BDNF) by upregulating the BDNF/TrkB/CREB signaling pathway, restored synaptic impairments and enhanced norepinephrine and serotonin levels. Their correlation analysis also showed that the gut barrier and lipopolysaccharide (LPS) content in the serum were strongly associated with behavioral performance and the biochemical indexes of the brain. Similarly, sesamol also can reduce the histological damage, MPO, and nitrite content in albino rats after 1 week of treatment 472.

#### C) Other lignans

Apart from the lignans from flaxseed or sesame, we must emphasize the potential applications of other lignans like schisandrin, magnolol, magnolin, honokiol, arctigenin, enterolactone, enterodiol, koreanaside A and fargesin.

**Schisandrin (SCH)** is a lignan and one of the primary active compounds from the widely used traditional medicinal plant *Schisandra chinensis* with sedative, hypnotic, anti-aging, antioxidant, and immunomodulatory properties 473. There are various types of SCH including SCH-A, B, or C. In a study 474 where mice were divided into six groups (control, model, 5-ASA, and SCH at varying doses), SCH-treated mice showed substantial weight gain, alleviated colitis severity, decreased inflammatory factors, and improved gut microbiota (GM) composition and bile acid conversion, suggesting SCH's potential as a UC treatment through the regulation of the SGK1/NLRP3 pathway and GM balance. Another study 475 demonstrated that SCH-B reduced IL-17A production in CD4+ T cells by targeting STAT3 *in vitro*. Notably, Sch B showed therapeutic effects on DSS-induced acute and chronic colitis and CD4+CD45RBhigh T cell-induced colitis, identifying TH17 cells as the direct target mediating its anti-inflammatory effects. The preventative effect of SCHB on UC and colitis-associated cancer was evaluated, showing that SCH-B enhanced intestinal epithelial barrier protection through FAK activation and regulates gut microbiota, both of which are crucial for its protective effects 476. SCH-B significantly reduced TNF-α, IL-1β, INF-γ, and IL-6 concentrations and mRNA expression levels in colon tissue and inhibited phosphorylation of IκBα, NF-κB p65, p38 MAPK, c-Jun NH2-terminal kinase, and extracellular signal-regulated kinase in DSS-induced UC mice 477. Schisandrin B demonstrated anti-inflammatory effects in both *in vivo* and *in vitro* colitis models by suppressing the NLRP3 inflammasome and inducing the AMPK/Nrf2 signaling pathway, protecting against ROS-induced mitochondrial damage and alleviating epithelial cell damage via regulation of pyroptosis 478. SCH-C also improved intestinal permeability dysfunction across three IBD model systems by enhancing epithelial barrier integrity through upregulation of ZO-1 and occluding 479. In Caco-2 cells, Schisandrin C reversed IL-1β-induced increases in MLCK and p-MLC expression, preventing cytoskeletal contraction and subsequent intestinal permeabilization. Additionally, Schisandrin C inhibited NF-ĸB and p38 MAPK signaling pathways, which regulate MLCK expression and tight junction complex reorganization. On the other hand, deoxyschisandrin, another lignan found in this Schisandra could ameliorate symptoms of UC, reducing the levels of inflammatory cytokines, suppressing CD4 T cell infiltration, and effectively inhibiting apoptosis in the colon of DSS-induced UC mice 480. Another study shows that this compound demonstrated a cytoprotective effect against H2O2-induced apoptotic cell death in human intestinal epithelial cells (HCT116). Deoxyschisandrin inhibited H2O2-induced apoptosis as evidenced by Annexin V and propidium iodide flow cytometry assays, and it blocked caspase-3 activation by preventing pro-caspase-3 cleavage. Additionally, deoxyschisandrin suppressed H2O2-induced NF-kappaB activation by inhibiting IkappaBalpha degradation and NF-kappaB translocation to the nucleus, suggesting its potential as a protective agent against oxidative stress-induced intestinal cell apoptosis 481.

**Magnolol and honokiol** are two lignans abundantly found in *Magnolia officinalis* plant with pleiotropic and anti-inflammatory actions 482. The use of *Magnolia officinalis* bark extract (MBE) formed by both magnolol and honokiol prevented weight loss and suppressed the activation of the proinflammatory cytokine IL6 in DSS-induced colitis. Besides, it restored the length of the damaged colon and decreased the expression of necroptosis markers in mice with DSS-induced colitis 483. Considering their properties separately, **magnolol** is a lignan that decreases the activity of pro-inflammatory cytokines such as IL-1β, IL-6, and TNF-α in DSS-treated mice 484. Magnolol demonstrated dose-dependent enhancement of phagocytosis and inhibition of NO production at the concentration range of 10-40 μM, whereas, in a DSS-induced colitis model, magnolol improved colitis symptoms, including body weight loss and colon length, attenuating pro-inflammatory cytokine levels and histopathological manifestations via modulation of MAPK and NF-κB signaling pathways 485. In rats with TNBS-induced colitis, magnolol at various doses significantly reduced colonic MPO activity and serum levels of IL-17. Histological analysis showed medium and high doses of magnolol improved DAI and thymus index, and downregulated NF-κB p65 mRNA and TLR-4 protein expression. It can also suppress IL-12 expression through modulation of NF-κB and PPAR-γ pathways and enhanced ZO-1 and occludin expression in colonic tissue 486. Similarly, **honokiol** can significantly improve DAI, colon length, and histopathological scores *in vivo*, and reduce inflammatory mediators while enhancing TJ proteins *in vitro* 487. In a mouse model of UC induced by DSS, honokiol administration suppressed proinflammatory cytokines TNF-α, IL-6, IL-1β, and IFN-γ, upregulated PPAR-γ expression, and inhibited the TLR4-NF-κB signaling pathway, attenuating gasdermin-D-mediated macrophage pyroptosis 488. Through bioactivity-guided chromatography and molecular experiments, HON was identified as a Transient receptor potential vanilloid 4 (TRPV4) antagonist, targeting the Q239 residue to inhibit channel opening and improve endothelial permeability 489. This mechanism enhanced VE-Cadherin expression and localization, protecting the endothelial barrier and suggesting potential therapeutic applications for alleviating IBD. Honokiol also inhibited caspase-1 activation, and apoptosis speck-like protein oligomerization, suggesting dual inhibition of NLRP3 inflammasome priming and activation processes through the modulation of the SLC3A2/L-leucine/mTORC1/NLRP3 pathways 490. Finally, Chen *et al.* 490 also demonstrated that honokiol could partially protect against colitis by regulating Th17 differentiation through activating SIRT3, leading to inhibition of the STAT3/RORγt signaling pathway.

**Magnolin** (MGL) is another lignan produced by plants of the Magnolia genus 491. Animal experiments demonstrated that MGL treatment mitigated weight loss, colon shortening, DA, colitis histological scores, and inflammatory factor expression 492. MGL also improved intestinal barrier function by preserving tight junction proteins (ZO-1 and Claudin-1) and inhibiting intestinal epithelial cell apoptosis induced by TNBS and TNF-α in mice and colon organoids. Furthermore, MGL exerted its protective effects by inhibiting the PI3K/AKT signaling pathway 492 and the arachidonate 5-lipoxygenase (ALOX5)-mediated ferroptosis, inhibiting M1 while promoting M2 macrophages 493.

**Arctigenin** is a natural lignan extracted from *Arctium lappa* with potential actions in IBD.

Arctigenin significantly mitigates colitis by reducing body weight loss, DAI, and histological damage in the colon. It enhances intestinal epithelial cell recovery, reduces neutrophil and macrophage infiltration, and suppresses pro-inflammatory cytokines and oxidative stress markers while inhibiting MAPK and NF-κB pathways 494. Similarly, this compound inhibits Th17 and Th1 cell differentiation by reducing STAT3 and STAT4 phosphorylation, respectively, and suppresses mTORC1 pathway activity independently of PI3K/AKT and ERK signaling in DSS-induced colitis mice 495. Orally administered arctigenin alleviated colitis in mice by enhancing mucosal healing, primarily through accelerating colonic epithelial cell migration without affecting proliferation through the activation of the focal adhesion kinase (FAK) 496.

**Enterolactone and enterodiol** are two lignans resulting from the metabolism of other lignans by the gut microbiota 497. Enterolactone was useful to mitigate inflammation-induced loss of intestinal epithelial barrier integrity and oxidative stress *in vitro* 498. In more detail, enterolactone at 200 nM maintained or improved Trans-Epithelial Electrical Resistance (TEER) values in HCT-8 and Caco-2/RAW-264.7 coculture models, enhancing ZO-1 protein expression in HCT-8 cells under inflammatory conditions. Likewise, **Koreanaside A**, a lignan isolated from the flower of *Forsythia koreana* alleviated inflammatory responses by downregulating AP-1, NF-κB, and JAK/STAT signaling in LPS-induced macrophages and DSS-induced colitis mice 499. **Fargesin**, another bioactive lignan isolated from *Flos Magnoliae*, has demonstrated anti-inflammatory effects on chemically induced IBD through NF-κB signaling suppression 499. Collectively, lignans seem to be interesting compounds to be used in patients with IBDs, although further studies are still required. ***Table 4*** summarizes the effects of the main lignans explored in this section.

### 2.5. Other types of polyphenols

#### 2.5.1. Curcuminoids (Curcumina, demetoxicurcumina, bisdemetoxicurcumina)

Curcuminoids are phenolic compounds commonly used as a spice, pigment, and additive also utilized as a therapeutic agent in several foods derived from the plant Turmeric (*Curcuma longa Linn*) 500. The main type of curcuminoid is the **curcumin**. Curcuminoids in general and curcumin, in particular, have proven to act as a potent anti-inflammatory and antioxidant agent 501. Likewise, they can work as anti-tumor, anti-apoptotic, anti-fibrosis, or immunomodulatory agents, having been investigated in a plethora of human diseases 502. In the context of IBDs, curcumin has presented a consistent body of evidence, acting through several mechanisms. The principal anti-inflammatory activity of this agent is through the inhibition of IBD inflammation by modulating the NF-κB pathway 503. Furthermore, it inhibits inflammatory cascades by suppressing the COX enzyme and prostaglandin E-2 (PGE2), also influencing various molecular signaling pathways related to inflammation, apoptosis, and oxidative stress, like PPARγ, PI3K, TLR-4, Akt, mTOR, ERK5, AP-1, TGF-β, PAK1, Wnt, β-catenin, Shh, Rac1, p38MAPK, EBPα, NLRP3 inflammasome, Nrf2, Notch-1, AMPK, STAT3, and MyD-88, crucial in IBD development 504. It also interacts with IL-1, IL-6, and IL-12, and decreases the levels of ROS having a good result in UC and CD patients 505. Also, curcumin reduced the expression of TNF-α, IL-6, and IL-17 while increasing the anti-inflammatory cytokine IL-10 506. In parallel, curcumin also modulates iron metabolism proteins and reduces iron stores in a DSS-induced colitis mouse model, which should be considered in IBD management by monitoring erythroid parameters 507. It may also interact with transient receptor potential vanilloid receptor 1 (TRPV1), attenuating visceral hyperalgesia and colitis 508. Finally, curcumin has also the ability to regulate autophagy, reducing the expression of key autophagy-related genes such as Beclin-1, ATG5, and LC3II, while increasing B cell lymphoma 2 (bcl-2), thereby improving colitis 509. Interestingly, preclinical models have shown that curcumin can decrease the expression of Th1 cytokines (IL-12, IFN-gamma, TNF-alpha, IL-1) and increase the expression of Th2 cytokines (IL-4 and IL-10) in colon mucosa, promoting a shift from Th1 to Th2 510. It should also be noted that curcumin use has some important challenges that need to be addressed. For instance, it is known that curcumin is unstable under physiological conditions and the bioavailability after oral administration of this compound is very low 511. Following oral administration, curcumin is quickly metabolized through reduction, sulfation, and glucuronidation in the liver, kidneys, and intestinal mucosa, leading to low intestinal absorption. The most common strategy to improve curcumin's poor pharmacokinetic profile involves combining it with piperine, a natural alkaloid found in black pepper (Piper nigrum), which effectively inhibits the glucuronidation process 512. To enhance its properties, researchers have also explored various approaches, including the development of compounds, the use of liposomes, and the synthesis of nanoparticles or curcumin analogs 513. Therefore, curcumin has the potential to modulate IBDs through several mechanisms described in preclinical models. However, distinctively compared to other polyphenols, curcumin stands out as the most extensively studied component in clinical trials and human studies. In the following subsection, the principal studies conducted in this field and their most relevant conclusions will be summarized.

##### Intervention studies in humans

Curcuminoids represent one of the most broadly explored polyphenols in the management of multiple diseases in humans, including in IBD. Indeed, various doses of curcumin have been tested and explored in patients with IBDs (i.e. 550 mg /three times daily-1 per month, and 1 g /twice times a daily-6 month) showing promising results that remain to be fully explored 514. In this subsection, the design and results obtained from the main clinical trials will be presented.

Most studies evaluating curcuminoids in humans have been conducted in UC patients. In a randomized, double-blind, multicenter trial 515, 89 patients with quiescent UC were given either curcumin (2g/day) plus sulfasalazine or mesalamine, or a placebo plus sulfasalazine or mesalamine for 6 months. Only 4.65% of the curcumin group relapsed compared to 20.51% in the placebo group, with significant improvements in both clinical activity index (CAI) and endoscopic index (EI) for the curcumin group. During a 6-month follow-up, 8 additional patients in the curcumin group and 6 in the placebo group relapsed, suggesting curcumin is a promising and safe maintenance therapy for UC remission. In another randomized, double-blind pilot trial involving 45 patients with mild-to-moderate distal UC participants received either NCB-02 (standardized curcumin preparation) enema plus oral 5-ASA or placebo enema plus oral 5-ASA. After 8 weeks, the NCB-02 group showed a 56.5% treatment response compared to 36.4% in the placebo group, with clinical remission in 43.4% versus 22.7% and endoscopic improvement in 52.2% versus 36.4%. Per protocol analysis indicated significantly better outcomes for the NCB-02 group in clinical response, clinical remission, and endoscopic improvement, suggesting that NCB-02 may offer greater improvements in disease activity for patients with mild-to-moderate distal UC 516. Lang *et al.* 517 developed a multicenter randomized, placebo-controlled, double-blind study of 50 mesalamine-treated patients with active mild-to-moderate UC. Curcumin (3 g/day) was given to 26 patients while 24 received a placebo for one month. Clinical remission (measured by Simple Clinical Colitis Activity Index- SCCAI ≤2) was achieved by 53.8% of the curcumin group compared to none in the placebo group, and clinical response (≥3 point reduction in SCCAI) occurred in 65.3% of the curcumin group versus 12.5% in the placebo group. Additionally, endoscopic remission (partial Mayo score ≤1) was seen in 38% of curcumin-treated patients compared to none in the placebo group, with adverse events being rare and similar between the groups. Similarly, Salomon *et al.* 518 conducted a placebo-controlled double-blind study to assess the efficacy of curcumin in inducing remission in 5-ASA treatment-resistant patients with mild-to-moderate UC. 50 patients with SCCAI scores between 5-12 were randomized to receive either 3 g of curcumin or placebo daily for 30 days alongside maximal 5-ASA therapy. Clinical remission, defined as SCCAI ≤2, was achieved in 54% (14/26) of curcumin-treated patients compared to none in the placebo group. A significant clinical response (≥3-point decrease in SCCAI) was observed in 65.3% of curcumin-treated patients versus 12.5% in the placebo group (P < 0.001, OR 13.2). Endoscopic remission (partial Mayo score ≤1) was achieved by 36% (8/22) of curcumin-treated patients and none in the placebo group (P = 0.035, OR 23.5). These findings highlight curcumin as a promising adjunct therapy for mild-to-moderate active UC, demonstrating efficacy in both clinical and endoscopic outcomes without notable side effects.

Simultaneously, in a randomized, double-blinded controlled trial involving 56 adults diagnosed with mild to moderate UC based on the SCCAI, participants were randomly assigned to receive either curcuminoids nanomicelles (80 mg, three times daily, orally) plus mesalamine (3 g/day, orally) or placebo plus mesalamine for four weeks 519. Assessments at baseline and weeks 2 and 4 showed a significant reduction in the urgency of defecation and improved general condition in the curcuminoid nanomicelles group compared to the placebo group. Additionally, the mean SCCAI score was significantly lower in the curcuminoid nanomicelles group at week 4 (1.71 ± 1.84) compared to the placebo group (2.68 ± 2.09, p = 0.050). This study suggests that adding curcuminoid nanomicelles to standard mesalamine therapy improves symptoms and reduces clinical activity in patients with ulcerative colitis In another clinical trial, Banerjee *et al.* 520 randomized mild to moderately active UC patients on standard mesalamine to receive either 50 mg twice daily bio-enhanced curcumin (BEC) or a placebo. At 6 weeks, clinical and endoscopic remission rates were 44.1% and 35.3% in the BEC group, respectively, compared to none in the placebo group, with significantly higher clinical response in the BEC group (52.9% vs. 14.3%). At 3 months, the BEC group had clinical remission, clinical response, and endoscopic remission rates of 55.9%, 58.8%, and 44%, respectively, compared to 5.7%, 28.6%, and 5.7% in the placebo group, with 95% and 84% of BEC responders maintaining remission at 6 and 12 months, and no significant side effects observed. In a randomized double-blind clinical trial conducted by Sadeghi *et al.* 521, 70 patients with mild-to-moderate UC were assigned to receive either curcumin (1,500 mg/day) or a placebo for 8 weeks. Curcumin supplementation significantly improved the SCCAI and scores on the Inflammatory Bowel Disease Questionnaire-9, enhancing quality of life Additionally, curcumin reduced serum hs-CRP levels and erythrocyte sedimentation rate (ESR) compared to placebo, highlighting its potential benefit as an adjunct therapy for mild-to-moderate UC alongside conventional treatments. Very recently, Ben-Horin *et al.* 522 evaluated the efficacy of the herbal combination of curcumin-QingDai (CurQD) in patients with active UC. The study consisted of two parts: In Part I of the study, an open-label trial evaluated the initial efficacy of CurQD in patients with active UC identified by specific clinical and endoscopic criteria. Part II involved a placebo-controlled trial where active UC patients were randomized in a 2:1 ratio to receive enteric-coated CurQD 3 g/d or placebo for 8 weeks, with continuation of maintenance therapy for responders. Then they focused on clinical response and endoscopic improvement. Part I demonstrated promising initial responses, with 70% achieving clinical improvement. Part II confirmed the efficacy of CurQD, showing significantly higher rates of clinical response (85.7% vs. 30.7%) and remission (50% vs. 8%) compared to placebo, alongside increased mucosal expression of CYP1A1, suggesting potential therapeutic benefits via the aryl-hydrocarbon receptor pathway. Also and as aforementioned, one study 445 analyzed the effects of the MD combined with curcumin and resveratrol supplementation on disease activity, serum inflammatory markers, and quality of life in patients with mild to moderate UC, showing that these interventions effectively reduced disease activity and inflammation while improving quality of life in UC patients.

Other studies have also found positive results regarding the role of curcumin in the management of CD. In a pilot study conducted by Holt *et al.* 523 five patients with ulcerative proctitis and five with CD were enrolled. Patients with ulcerative proctitis were treated with 550 mg of curcumin twice daily for one month, followed by 550 mg three times daily for another month, while CD patients received 360 mg of curcumin three times daily for one month and then four times daily for the remaining two months. Assessments included blood tests, inflammation markers (sedimentation rate and C-reactive protein), sigmoidoscopies, and biopsies at baseline and the end of the study. Results showed improvements in symptoms and reduced medication use in ulcerative proctitis patients, while CD patients exhibited lowered DAI scores and sedimentation rates following curcumin therapy. Theracurmin®, a highly bioavailable curcumin derivative with potent anti-inflammatory properties, was evaluated in a randomized, double-blinded study involving patients with active mild-to-moderate CD 523. Administered at 360 mg/day for 12 weeks, Theracurmin® demonstrated significant reductions in clinical disease activity by week 12 (p = 0.005) and achieved clinical remission rates of 35%, 40%, and 40% at weeks 4, 8, and 12, respectively. Endoscopic remission rates and healing of anal lesions were also notable in the Theracurmin® group compared to placebo, with no serious adverse events reported. In a clinical trial conducted in a pediatric population 524, children with CD or UC in remission or with mild disease (Pediatric Crohn's Disease Activity Index [PCDAI] <30 or Pediatric Ulcerative Colitis Activity Index [PUCAI] score <34) received curcumin alongside standard therapy. Starting at 500 mg twice daily for 3 weeks, doses were escalated to 1 g twice daily at week 3 and to 2 g twice daily at week 6 using a forced-dose titration design. Measures of disease activity (PUCAI/PCDAI) and side effects were assessed at weeks 3, 6, and 9. Curcumin was well tolerated overall, with increased gassiness reported by only 2 patients consistently across visits. This study suggested that curcumin could be considered as adjunctive therapy alongside conventional treatments for patients seeking combined conventional and alternative medicine approaches.

Despite these positive results, some studies have failed to find significant benefits from curcumin in the management of IBD. A double-blind randomized controlled trial across 8 centers in France included 62 patients with CD post-bowel resection who received azathioprine and were randomly assigned to either oral curcumin (3 g/day) or placebo for 6 months 525. The evaluation included a colonoscopy, CD activity index, laboratory tests, and quality of life questionnaires. At month 6, postoperative recurrence (Rutgeerts' index score ≥i2) was observed in 58% of the curcumin group versus 68% of the placebo group, with a higher incidence of severe recurrence (Rutgeerts' index score ≥i3) in the curcumin group. Clinical recurrence rates and quality of life scores did not significantly differ between groups. Severe adverse events were reported in 6% of the placebo group and 16% of the curcumin group. The study concluded curcumin showed no superior efficacy over placebo in preventing CD recurrence post-surgery with thiopurine treatment. Likewise, Kedia *et al.* 526 conducted an 8-week randomized clinical trial to investigate the effectiveness of oral curcumin in achieving clinical remission among patients with mild-to-moderate ulcerative colitis. Participants were assigned to receive either mesalamine combined with curcumin (450 mg/day) or mesalamine with a placebo. The study outcomes showed no significant differences between the curcumin and placebo groups in terms of rates of treatment failure, mucosal healing, clinical response, or clinical remission by the end of the trial. These findings suggest that a daily dose of 450 mg of curcumin did not successfully induce remission in patients with mild-to-moderate ulcerative colitis.

Based on the collective findings from various clinical trials exploring the efficacy of curcuminoids in IBD, including UC and CD, several conclusions can be drawn. Curcuminoids, investigated at varying doses and formulations, demonstrate promising potential in managing IBD, particularly in inducing and maintaining remission in patients with mild-to-moderate disease activity. Studies have consistently shown significant improvements in clinical response, endoscopic remission, and reduction of inflammatory markers with curcumin supplementation, suggesting its role as an effective adjunct therapy alongside conventional treatments. Systematic reviews and meta-analysis agree that the use of curcumin and curcuminoids in the management of both UC and CD show promising results, with a greater level of evidence in the case of the former 527. However, they also sustain that there is a need to standardize the dose and the formulations of curcumin, the time of treatment, and the route of administration before drawing definitive conclusions 528. Moreover, conflicting results from certain trials underscore the variability in outcomes and highlight the need for further large-scale, multicenter studies to validate these findings.

#### 2.5.2. Polyphenols from olives and extra virgin olive oil

Olives and EVOO stand out as one of the most representative foods of MedDiet. EVOO contains over 30 phenolic compounds, including the most represented oleuropein (OLE), both in the glycated and in the aglycone form, hydroxytyrosol (HT), and others like verbascoside, oleocanthal or oleachin 529. Olives and EVOO act as potent immunomodulatory, antioxidant, antiapoptotic, anti-inflammatory, and antimutagenic agents, with significant benefits in the different organs and systems of the body, including the digestive system and the gut 530,531. Numerous preclinical studies have exhibited solid evidence of the mechanisms by which polyphenol-rich EVOO or specific polyphenols isolated from these compounds provide their anti-inflammatory, antioxidant, antitumor, and microbiota-modulation effects whereas some human studies that explored the effects of EVOO on patients with IBD further support its relevance 532. Herein we will summarize the main evidence around EVOO, HT, and oleuropein conducted in the field of IBDs.

##### A) Extra virgin olive oil

Preclinical models conducted *in vitro* and *in vivo* have shown multiple roles from EVOO in the management of IBD. The impact of olive oil phenolic extract in the inflammatory response was evaluated by Muto *et al.* 533 in Caco-2 cells treated with LPS or IL-1β. This compound was found to prevent IL-8 expression and secretion in LPS-treated cells, while in IL-1β-treated cells, it was able to inhibit IL-8 promoter activity but enhanced IL-8 mRNA stability, leading to increased protein expression, with involvement of the p38 and ERK signaling pathways. Also, different variants of EVOO from Spain were shown to exert protective effects on cell integrity and a reduction in ROS production in Caco-2 cells, highlighting the health benefits of the Picual variety 534. Likewise, antioxidant polyphenols from EVOO can decrease cytotoxicity and ROS production in Caco-2 cells exposed to alternariol, a mycotoxin that can contaminate olives 535. In animal models, EVOO diets exerted a noteworthy beneficial effect in chronic DSS-induced colitis by cytokine modulation and COX-2 and iNOS reduction via downregulation of p38 MAPK, with greater results if the diet is supplemented with HT 536. In another study 537, mice on an EVOO+polyphenol extract (PE) diet showed significantly reduced DAI, cell proliferation, and levels of MCP-1, TNF-α, COX-2, and iNOS compared to the control group, as well as down-regulated JNK phosphorylation, prevented IκBα degradation, and PPARγ deactivation. They concluded that EVOO-PE supplementation effectively mitigates experimental colitis through PPARγ up-regulation and inhibition of nuclear transcription factor-kappa B and MAPK signaling pathways. Similarly, Takashima *et al.* 538 showed the effect of chronic intake of 5% EVOO on inflammation, cell proliferation, and signal transducers and activators of transcription (STAT) in a DSS-induced colitis rat model. EVOO significantly attenuated inflammation as assessed by disease activity index, body weight loss, and histological scores. It also reduced the expressions of STAT3, phosphorylated STAT3 (pSTAT3), COX-2, and iNOS induced by DSS, while mitigating increases in cell proliferation (PCNA) and restoring apoptosis (cleaved caspase-3).

Despite these favorable results, other studies have failed to find any significant benefits from EVOO in the management of IBD. HLA-B27 transgenic rats fed diets containing corn oil (CO), EVOO with high phenolic content, or olive oil with low phenolic content (ROO) for 3 months showed that CO-induced intestinal inflammation characterized by diarrhea, increased myeloperoxidase activity, and mucosal injury, which was not mitigated by EVOO 539. Nonetheless, EVOO significantly reduced TNFα gene expression in the colon mucosa and lowered total cholesterol levels compared to CO-fed rats, suggesting potential benefits in managing hypercholesterolemia and minimizing statin-associated myotoxicity. Another work 540 investigated the effects of dietary interventions with EVOO and fish oil (FO) on a DSS-induced colitis mouse model. Despite dietary supplementation with EVOO, FO, or a combination of both, none of the interventions ameliorated symptoms or inflammatory markers associated with colitis. Additionally, mice supplemented with FO showed an increased spleen weight-to-body weight ratio, while the combination of EVOO and FO led to elevated TNF-α levels compared to controls.

##### Intervention studies in humans

To date, few clinical trials have been conducted evaluating the effectiveness of EVOO and their polyphenols in the clinical management of IBD. In a crossover clinical trial involving forty patients with UC, Morvaridi *et al.* 541 showed that consumption of EVOO significantly decreased erythrocyte sedimentation rate and hs-CRP. Gastrointestinal symptoms including bloating, constipation, fecal urgency, incomplete defecation, and overall gastrointestinal symptom severity (GSRS) were also notably reduced after EVOO consumption. In a randomized, controlled, double-blind, crossover trial involving 12 participants 542, ingestion of three raw virgin olive oils differing in phenolic compound (PC) content was assessed over 3-week periods with intervening washout periods. PC-enriched virgin olive oil containing a mixture of olive oil and thyme (FVOOT) significantly decreased serum ox-LDL levels and increased bifidobacteria populations and phenolic metabolite protocatechuic acid compared to baseline and to virgin olive oil naturally containing PC (VOO) (P < 0.05). These findings suggest that PC-enriched olive oils, particularly FVOOT, may confer cardioprotective benefits through modulation of microbial populations and antioxidant metabolites. It must be highlighted that despite additional studies in humans are still required, the inclusion of EVOO and other food sources contained in MedDiet has proven significant benefits in patients with IBDs. Following 6 months of MedDiet resulted in improved BMI, reduced waist circumference, and decreased prevalence of liver steatosis in both UC and CD patients 543. Furthermore, fewer patients experienced active disease and elevated inflammatory biomarkers after adhering to the MedDiet, indicating potential benefits for managing IBD-related symptoms and metabolic health. In a prospective, randomized controlled trial from 2017 to 2021 involving adults with quiescent ulcerative colitis (UC), participants were assigned to either a Mediterranean diet pattern (MDP) or a control high-fiber diet (CHD) for 12 weeks 544. The MDP group showed higher tolerance and significantly lower levels of fecal calprotectin (>100 μg/g) compared to the CHD group (75% vs. 20%). Additionally, the MDP group exhibited increased levels of total fecal short-chain fatty acids (SCFAs), specifically acetic acid and butyric acid, and favorable changes in gut microbiota associated with protective roles in colitis, such as Alistipes finegoldii and Flavonifractor plautii, as well as SCFA-producing Ruminococcus bromii. These findings suggest that the MDP may serve as a beneficial and sustainable dietary approach for maintaining clinical remission and managing UC. Importantly, MedDiet shows similar benefits to other well-established diets like Specific Carbohydrate Diet (SCD) to achieve symptomatic remission and regulate different clinical variables, defining the adherence to a diet as the most important factor to maximize the benefits from these approaches 545.

Overall, despite more clinical trials and human studies specifically focusing on EVOO and its polyphenols are warranted, the inclusion of this food and nutrients in MedDiet is a promising translational strategy for patients with IBDs.

##### B) Hydroxytyrosol

HT (3,4-dihydroxyphenyl-ethanol) is one of the most important compounds found in EVOO and is broadly studied in preclinical models of IBD. Elmaksoud *et al.* 546 investigated the therapeutic potential of olive leaf extract standardized with 25% hydroxytyrosol (OLES-25%HYT) in treating induced ulcerative colitis in albino rats. Compared to untreated ulcerative colitis rats, OLES-25%HYT significantly reduced mortality rate and DAI while also decreasing oxidative stress markers such as MDA, MPO, and NO and increasing antioxidant enzymes including SOD, CAT, and GPX in colon tissue. Additionally, OLES-25%HYT downregulated pro-inflammatory cytokines and the apoptotic gene Bax, while upregulating the anti-apoptotic gene Bcl2, demonstrating its intestinal anti-inflammatory, antioxidant, and anti-apoptotic effects in experimental ulcerative colitis models. Mao 547 also showed that HT supplementation ameliorated colon pathology and apoptosis, increased antioxidant capacity, and reduced expression of NLRP3 inflammasome components and pro-inflammatory cytokines in the DSS-induced colitis model. Additionally, HT promoted a favorable shift in gut microbiota composition towards probiotics and increased levels of short-chain fatty acids, highlighting its potential therapeutic benefits in ulcerative colitis through multiple mechanisms. HT is also able to counteract the effects of Benzo[a]pyrene (B[a]P) in human colonic epithelial cells (HCoEpC), decreasing the production of pro-inflammatory cytokines and ERK1/2 and mTOR activation while promoting autophagy and mitophagy 548. HT alone or combined with pectin/alginate and olive oil seems to be effective against inflammation in TNBS-induced colitis, alleviating inflammatory infiltration 549. *In vivo* and *in vitro* models show that medium-chain triglycerides led to greater damage and colitis symptoms, while the use of HT with eicosapentaenoic acid and docosahexaenoic acid significantly reduced damage and inflammation, as well as the combined use of fish and olive oil 550. Sanchez-Fidalgo *et al.* 551 divided six-week-old mice into three dietary groups: standard, EVOO, and HT-enriched EVOO, and then induced with colitis using 3% DSS. Diets enriched with EVOO significantly reduced clinical and histological damage, decreased mortality by about 50%, and improved cytokine profiles, including maintaining TNF-α near control levels and increasing IL-10, while downregulating COX-2 and iNOS. HT supplementation further enhanced these benefits, especially in reducing iNOS levels and adding antioxidant effects, suggesting improved outcomes for chronic colitis management.

##### C) Oleuropein

OLE is a secoiridoid, an ester of elenolic acid and HT with a oleosidic skeleton. Past works have remarked on the potential translational role of OLE in the context of IBDs. An experimental study using rat models of UC compared the effects of this compound with normal controls and untreated colitis-induced 552. Colonic tissue analysis showed OLE significantly reduced oxidative stress markers (MDA, MPO, NO) and increased antioxidant enzyme levels (SOD, CAT, GPX) while downregulating pro-inflammatory cytokines and the pro-apoptotic gene Bax and upregulating the anti-apoptotic gene Bcl2. OLE demonstrated significant intestinal anti-inflammatory, antioxidant, and anti-apoptotic effects, reducing both the mortality rate and disease activity index in ulcerative colitis. In another work, the anti-inflammatory effects of OLE in a mouse model of chronic colitis induced by DSS were evaluated 553. Mice receiving a diet supplemented with 0.25% OLE for 56 days showed reduced inflammatory symptoms, improved disease activity index, and histopathological changes, alongside decreased inflammatory cell recruitment and lower levels of IL-1β and IL-6, with increased IL-10 in colon tissue. OLE also reduced COX-2 and iNOS expression, suppressed p38 MAPK phosphorylation, potentially upregulated annexin A1, and improved intestinal wound healing, indicating its promise as a treatment for UC. Biopsies from 14 patients with active UC were cultured with *Escherichia coli* lipopolysaccharide (EC-LPS) and OLE OLE treatment significantly reduced the expression of cyclooxygenase-2 (COX-2) and interleukin-17 (IL-17), as well as IL-17 levels in culture supernatants, compared to EC-LPS treatment alone. Histological analysis showed that OLE-treated samples had reduced inflammatory cell infiltration and improved inflammatory damage, demonstrating OLE's anti-inflammatory activity in UC colonic biopsies 554.

#### 2.5.3. Coumarins

Coumarins are a class of benzopyrones, consisting of a benzene ring fused to an alpha-pyrone ring, also entailing important effects on human health and promising translational implications 555. Coumarins can be found in vegetables, spices, fruits, and medicinal plants including all parts of the plants-fruits, roots, stems and leaves; however cinnamon stands out as one of the edible foods with the highest concentration of coumarins 556. The main types of coumarins include esculetin, aesculin, umbelliferone, fraxetin, daphnetin, coumarin, and paepalentin.

Esculetin is a coumarin compound derived from the bark of *Fraxinus chinensis Roxb* and its glycoside form is called aesculin. Both compounds are characterized by exerting potent antioxidant and anti-inflammatory properties 557. **Esculetin**, administered at 5 mg/kg in a rat model of TNBS-induced IBD, demonstrated potent intestinal anti-inflammatory effects, preventing an increase in MDA levels, countering GSH depletion, reducing epithelial cell apoptosis, and inhibiting the secretion of pro-inflammatory cytokines (IL-1β, IL-2, IFN-γ) *in vitro*. *In vivo*, esculetin reduced colonic TNF-α and IL-1β levels and inhibited MPO and alkaline phosphatase activities, being effective at a 10-fold lower dose than sulphasalazine and showed comparable efficacy to prednisolone 558. Using network pharmacology and molecular docking, one study 559 identified 50 potential gene targets of esculetin against UC, including core genes such as AKT1, STAT1, CCND1, and PTGS2. Pathway enrichment analysis revealed the prolactin (PRL) signaling pathway as crucial in esculetin's action against UC, supported by strong molecular docking affinity to PRL and its receptor. Another research explored the role of esculetin and **4-methyl-esculetin** in a rat model of colitis induced by TNBS 560. Esculetin reduced lesion extension, and diarrhea incidence, and restored GSH levels in acute colitis, whereas 4-methyl-esculetin seemed to exert a superior efficacy, also inhibiting MPO and alkaline phosphatase activities, beneficial in acute and relapse colitis models likely due to its methyl group at C-4. Indeed, more studies have proven the efficacy of 4-methyl-esculetin in IBD models.

In a TNBS-induced rat colitis model, 4-methyl-esculetin exhibited efficacy comparable to prednisolone and sulfasalazine, reducing macroscopic damage scores, restoring intestinal architecture, and preventing GSH depletion and alkaline phosphatase activity 561. During colitis relapse, it improved histological inflammation and biochemically inhibited MPO, alkaline phosphatase, and metalloproteinase 9 activities, while also decreasing MDA and IL-1β levels. *In vitro*, 4-methyl-esculetin inhibited IL-1β, IL-8, IL-2, and IFN-γ production, indicating its potential as a potent intestinal anti-inflammatory agent. Similar results were obtained in DSS-induced colitis mice, as 4-methyl-esculetin administered orally at 25 mg/kg daily, improved microscopic parameters, reduced MPO activity, lowered colonic IL-6 levels, and prevented GSH depletion compared to the DSS-control group 562.

On the other hand **aesculin** demonstrated minimal cytotoxicity *in vivo* and in RAW264.7 macrophages, significantly alleviated DSS-induced colitis symptoms, and reduced inflammatory factors such as iNOS, IL-1β, and TNF-α in both peritoneal macrophages and colonic tissues 563. Importantly, aesculin attenuated NF-κB signaling activity and enhanced PPAR-γ nuclear localization in DSS-induced mice and LPS-stimulated macrophages, suggesting its therapeutic potential in UC through modulation of these pathways.

Other types of coumarins have also shown significant benefits in preclinical models of IBD. Witaicenis *et al.* 564 evaluated six coumarin derivatives of plant origin (scopoletin, scoparone, fraxetin, 4-methyl-umbelliferone, esculin, and daphnetin) in TNBS-induced colitis in rats. They reported that treatment with aesculin, **scoparone, and daphnetin** produced the best protective effects. In their experiments, all coumarin derivatives showed antioxidant activity in the DPPH assay, while daphnetin and **fraxetin** also showed antioxidant activity by inhibiting lipid peroxidation. Coumarins, except for 4-methyl-umbelliferone, also showed antioxidant activity through the counteraction of GSH levels or the inhibition of MPO activity. He *et al.* observed that **daphnetin** effectively alleviated colitis severity in mice by improving intestinal structure and increasing levels of ZO-1, occludin, and BCL-2, while reducing Bax and cleaved caspase 3 expression 565. Daphnetin also suppressed MDA and SOD activities, inflammatory cytokine levels, and apoptosis in both *in vivo* and *in vitro* models. Mechanistically, daphnetin inhibited JAK2/STAT signaling via REG3A activation, highlighting its potential therapeutic role in UC treatment by targeting these pathways 565.** Fraxetin** mitigated DSS-induced symptoms including body weight loss, colon shortening, tissue damage, and DAI. Mechanistically, this compound can inhibit the NF-κB pathway and NLRP3 inflammasome activation, reducing inflammatory responses, restoring gut barrier function by enhancing goblet cells and tight junction proteins (ZO-1 and occludin) and modulating intestinal microbiota diversity 566.

**Umbelliferone (UMB)**, a potent coumarin derivative with antioxidant and anti-inflammatory properties, was investigated in an acetic acid-induced UC rat model 567. UMB (30 mg/kg, oral) significantly improved macroscopic and histological tissue damage, reduced colonic TNF-α, IL-6, MPO, and VCAM-1 levels, and downregulated TLR4, NF-κB, and iNOS gene and protein expression, indicating strong anti-inflammatory effects. Additionally, UMB upregulated SIRT1 and PPARγ signaling pathways, mitigating oxidative injury and inflammation 568.

**Paepalantine** (9,10-dihydroxy-5,7-dimethoxy-1H-naptho(2,3c)pyran-1-one) is an isocoumarin previously isolated from the capitula of the Brazilian endemic *Paepalanthus bromelioides.* Administered at doses of 5 and 10 mg/kg, paepalantine significantly mitigated TNBS-induced colonic damage in both intact mucosa and mucosal recovery settings, as demonstrated histologically and biochemically 569. This protective effect was attributed to paepalantine's ability to enhance colonic oxidative status by preventing glutathione depletion and inhibiting colonic nitric oxide activity.

Lastly,** Coumarin** and its derivate, 4-hydroxycoumarin were evaluated in TNBS-induced colitis models of rats 570. Both compounds, administered at doses of 5 and 25 mg/kg, significantly reduced colonic damage, observed macroscopically, microscopically, and biochemically. This protective effect was associated with the prevention of GSH depletion due to colonic inflammation. Overall, the literature supports the relevance of coumarins and derivates in preclinical models of IBD, although studies in humans to confirm their applications are warranted.

#### 2.5.4. Flavonolignanes (Silimarin)

**Flavonolignans** are the major bioactive components presented in the Milk thistle (*Silybum marianum*) mostly known for their hepatoprotective properties, but also standing out for their immunomodulatory, antioxidant, and antitumoral role 571. Flavonolignans in *S. marianum* (collectively known as silymarin) are structurally diverse, with 23 constituents being isolated from purple- and white-flowering variants. Flavonolignans, compounds found in silymarin, are often mischaracterized by their name, as they are not composed of separate flavonoid and lignan units. Instead, they are biogenetically related to lignans and neolignans, sharing similar biosynthetic pathways, and are derived from two phenylpropanoid units with an additional structure that classifies them under flavonoids 572. These compounds exhibit a wide structural diversity due to various linkages of the C6C3 unit to the flavonoid nucleus, often forming dioxane, furan, cyclohexane rings, or simple ether side chains, and they frequently exist as stereoisomers in nature. The 3 more important isomers are silybin (or silibinin), silydianin, and silychristin, being the former the most active of these compounds 573.

**Silymarin (SM)** is a potent anti-inflammatory molecule capable of inhibiting NF-κB pathways and optimizing the redox balance in the cell through activating antioxidant enzymes and non-enzymatic antioxidants via Nrf2 activation 574. Also, some studies have remarked on the relevance of SM and quercetin as non-toxic and safe bioactive compounds with a marked antiviral activity, being suggested as a potential tool against viral-associated IBD 575. Mechanistically, the protection and antiviral effects of these bioactive compounds include a decrease in oxidative damage, inhibition of viral binding and replication, RNA synthesis, caspase enzymes, viral proteases, and viral assembly. Another study 576 investigated the effects of SM and L-arginine (L-Arg) on IBD progression in a TNBS-induced colitis rat model, finding that both treatments ameliorated colitis symptoms, decreased serum TNF-α levels, inhibited colonic iNOS, NF-κB, and cytochrome c expression, and increased HSP70 expression. The oral bioavailability of SM is poor due to rapid metabolism, low intestinal permeability, and poor water solubility; and varies significantly between species and is influenced by the methods of preparation 574. Because of this, some studies have developed different systems to improve silymarin's bioavailability, with promising results obtained when compared to conventional approaches 577,578.

**Silibin** has also proven significant benefits in preclinical models of IBD. A rat model of colitis induced by TNBS to evaluate the anti-inflammatory effects of silibinin and ursodeoxycholic acid (UDCA), alone and in combination, with dexamethasone as a control. The treatments significantly reduced NF-κB activity, IL-1β, TNF-α, TBARS, protein carbonyl, and MPO levels, while enhancing antioxidant power, with combination therapy showing the most notable improvements 579. Chemopreventive effects of silibin were evaluated on colitis-associated cancer (CAC) mouse model, finding that this component significantly inhibited intestinal tumor cell viability, reduced tumor size and number, decreased colitis and tumor scores, and promoted apoptosis while inhibiting proliferation 580. Additionally, silibinin reduced inflammatory cytokine production, improved the colonic mucosal barrier, and suppressed STAT3 phosphorylation, indicating its potential as a chemopreventive agent against CAC.

##### Intervention studies in humans

A randomized, double-blinded, placebo-controlled clinical trial involving 80 UC patients in remission was conducted from September 2009 to October 2010 581. Patients were randomly assigned to receive either SM (140 mg daily) or placebo (composed of lactose monohydrate, corn starch, and magnesium stearate) alongside their standard therapy for 6 months. Ten patients discontinued the study due to adverse effects (4 in the SM group) or disease flare-ups (6 in the placebo group). The SM group showed significant improvements in hemoglobin levels and erythrocyte sedimentation rate, as well as a decrease in DAI. After 6 months, 35 out of 38 patients in the SM group remained in complete remission without flare-ups, compared to 21 out of 32 patients in the placebo group. These findings suggest that SM supplementation may effectively help maintain remission in UC patients. Overall, ***table 5*** summarizes the effects of the rest of the polyphenols explored in this section.

## 3. Understanding the precise role of polyphenols as part of an integrative therapy in IBD

As explained before IBDs, represented by UC and CD, are two major inflammatory conditions potentially characterized by an abnormal mucosal immunological response associated with a heterogeneous phenotypic spectrum, triggered by gastrointestinal and extraintestinal affectations, or with atypical or non-specific symptoms 582. Although conventional treatments have been shown to be effective in controlling symptoms, many patients experience significant side effects derived from their use, and a percentage of them do not respond adequately to these therapies 583. In this context, the use of natural compounds, such as dietary polyphenols could be of great aid to avoid the negative effects of normal therapies, acting as complementary therapies from the available armamentarium 584. Throughout the present manuscript, the relevance of the main types of polyphenols and their mechanisms of action in IBDs have been reviewed. However, to fully understand the role of polyphenols in the medical management of these conditions, it is necessary to focus on the critical points and potential limitations derived from their use. These issues mainly revolve around the bioavailability of polyphenols, potential interactions with other treatments, and security considerations.

### 3.1. Bioavailability of polyphenols

Regarding the formulations and dosages of polyphenols, it is crucial to consider the bioavailability and stability of these compounds, as well as the most appropriate route of administration.

The poor bioavailability of polyphenols represents one of the major concerns to be addressed. The literature 585 highlights that polyphenols show a low bioavailability due to their interaction with other nutrients along with metabolic processes mediated by the liver (phase I and II metabolism), intestine, and microbiota. Oral administration is the most usual pathway for polyphenols given pharmacologically, leading to these problems of bioavailability 586. In the case of humans, it is difficult to imagine other pathways of administration, due to the confortability of using oral pills. Various formulations, such as liposomes, pro-liposomes, micro- and nanocapsules, polymeric capsules, phospholipid-polyphenol complex, micro- and nanoemulsions, micro- and nanoparticles, or even more complex systems are being explored to improve the delivery and therapeutic efficacy of polyphenols in patients with IBD 587,588. However it should be noted that the biological activities and therapeutic effects of polyphenols are also potentially mediated by their metabolites formed in the gut, liver, or gut microbiota, therefore demonstrating the complexity of polyphenols in this context 589,590. In general, gallic acid and isoflavones are the most well-absorbed polyphenols, followed by catechins, flavanones, and quercetin glucosides, although with different kinetics. Proanthocyanidins, the galloylated tea catechins, and the anthocyanins are the least well-absorbed polyphenols 590. These properties are directly related to other important points such as finding adequate doses of polyphenols. In the case of clinical applications of polyphenols in IBD, the optimal dosage may vary depending on the type of polyphenol, and also on the severity of the disease. Thus, in the case of polyphenols with poor bioavailability like quercetin or RES, a long period of administration together with high daily doses of these compounds are generally required for observing their therapeutic effects, as previously described 591. Therefore, issues regarding the optimal dosage, vehicles of administration, or specific considerations related to the bioavailability of each polyphenol a pivotal points of research to be explored by further studies.

The inclusion of polyphenols from diets and foods rich in these compounds is also a critical point of study. The literature has shown the promising adjuvant effects of different dietary patterns characterized by high content of polyphenols on the clinical management of IBDs including the anti-inflammatory 592, Mediterranean 593, and FODMAP diets 594. With specific differences and punctualizations, these dietary patterns include plant-based sources rich in polyphenols, including vegetables, legumes fruits and spices. However, to understand the role of polyphenols contained in different food sources, a critical concept should be introduced herein: the food matrix. The literature defines a food matrix as the “physical domain that contains and/or interacts with specific constituents of a food (e.g., a nutrient) providing functionalities and behaviors which are different from those exhibited by the components in isolation or a free state” 595. This means that for example, isolated quercetin exerts different effects than quercetin contained in food sources like broccoli, as quercetin would be included in a broad and complex food matrix, interacting with other nutrients that may affect its activity. The relevance of the food matrix in the bioavailability of polyphenols is perhaps one of the most interesting topics of nutrition research. Indeed, the literature recognizes that the food matrix can either enhance or diminish the bioavailability of polyphenols, although the mechanism by which it works remains to be fully elucidated 596. Bohn 597 conducted an extensive review detailing how dietary factors and food matrix affected polyphenol bioavailability. In this review, it is highlighted that dietary fiber (such as hemicellulose), divalent minerals, and viscous and protein-rich meals are likely to cause detrimental effects on polyphenol bioaccessibility; whereas digestible carbohydrates, dietary lipids (especially for hydrophobic polyphenols, e.g., curcumin), and additional antioxidants may enhance polyphenol availability Likewise, after epithelial uptake, polyphenols such as flavonoids may diminish phase II metabolism and excretion to enhance polyphenol bioavailability, whereas various polyphenols may act synergistically due to their influence on efflux transporters such as p-glycoprotein 597. Other examples more specific include the effects of pectin with quercetin, improving its bioavailability through gut flora and function changes; the sugar content in matrices affecting RES bioavailability, with lower absorption in grape juice compared to pure forms and also there are mixed results on milk's impact on polyphenol bioavailability 598. The fat content of cocoa enhances the digestibility of some phenolic compounds (especially procyanidins) 599, whereas EVOO polyphenols appear to be highly bioavailable with greater absorption in a dose-dependent way and when administered as an olive oil solution compared to an aqueous solution 598. Likewise, as occurring with oral supplementation, the bioavailability of polyphenols present in foods is in general low, and *in vitro,* studies observed clear differences in the mechanisms of polyphenols when used in their pure form and at high concentrations versus when ingested through foods rich in these compounds 600. This, however, does not mean that they do not have favorable or preventive effects against different pathologies, but it demonstrates that the food matrix is an important element to be considered in the therapeutic effects of polyphenols. Therefore, the consideration of the food matrix and specific combinations of polyphenols with other nutrients can be used to maximize the benefits of these compounds.

### 3.2. Safety considerations and interaction of polyphenols with available therapies

Notwithstanding we have strongly supported the benefits of polyphenols in the clinical management of IBDs, it is important to remark that polyphenols can also have certain harmful effects that are worth mentioning. Among other effects, polyphenols can block iron uptake, inhibit digestive enzymes, alter intestinal microbiota, and hormonal balance, and interact with different drugs 601, which may lead to undesired adverse effects in these patients.

Regarding the point, it is well-established that one of the most common complications seen in IBD patients is iron deficiency anemia (IDA) 601. Despite iron can be supplemented intravenously, oral supplementation of this mineral is the most common therapeutic option. Polyphenols are able to chelate the ions of transition metals (e.g., Fe and Cu), inhibiting the formation of free radicals in the Fenton and Haber-Weiss reactions 601. Catechins are one of the most important inhibitors of iron absorption 602. Despite in general this action of polyphenols is considered positive as part of their antioxidant properties; this effect could be particularly detrimental for IBD patients with IDA. For instance, past works have demonstrated that 20-50 mg total polyphenols/serving reduced Fe absorption from the meal by 50-70%, whereas beverages containing 100-400 mg total polyphenols/serving reduced Fe absorption by 60-90% 603. Therefore, the studies discouraged the use of black and herb teas, coffee, and coca for patients with low levels of iron, with the greatest detrimental effects for black tea.

On the other hand, flavonoids can form complexes with proteins through nonspecific forces such as hydrogen bonding and hydrophobic effects, as well as by covalent bond formation 604. This effect can affect the utilization of food proteins and digestive enzymes, which may lead to impaired function and disturbances during biochemical reactions or processes that a given enzyme catalyzes. Digestive enzymes have been shown to present significant benefits in the clinical management of gastrointestinal diseases like IBD 605,606. Therefore, if digestive enzymes are used in IBD patients, the effect of flavonoids in regulating these proteins should also be considered.

The role of polyphenols on gut microbiota and hormonal balance are perhaps two concerns more complex and less studied. In the case of the former, and as it has been remarked throughout the whole manuscript, polyphenols partly exert their therapeutic action by modulating the gut microbiota. In general, these effects are considered positive; however, the precise role of gut microbiota in IBDs is not fully understood yet, and some specific individual factors and variations should be considered to understand if the changes that occurred in these microbial are favorable, neutral or harmful 607. Therefore, more efforts are needed in this sense. Secondly, the available literature recognizes a possible role of isoflavones in the alteration of the hormonal balance, affecting both estrogen and thyroid hormone regulation. However, there is a great heterogeneity in the available studies regarding these effects. As IBD might be associated with estrogen and thyroid hormone dysregulation 608,609, further studies should be conducted to evaluate potential side effects derived from the use of isoflavones in IBD.

**Figure 4:**
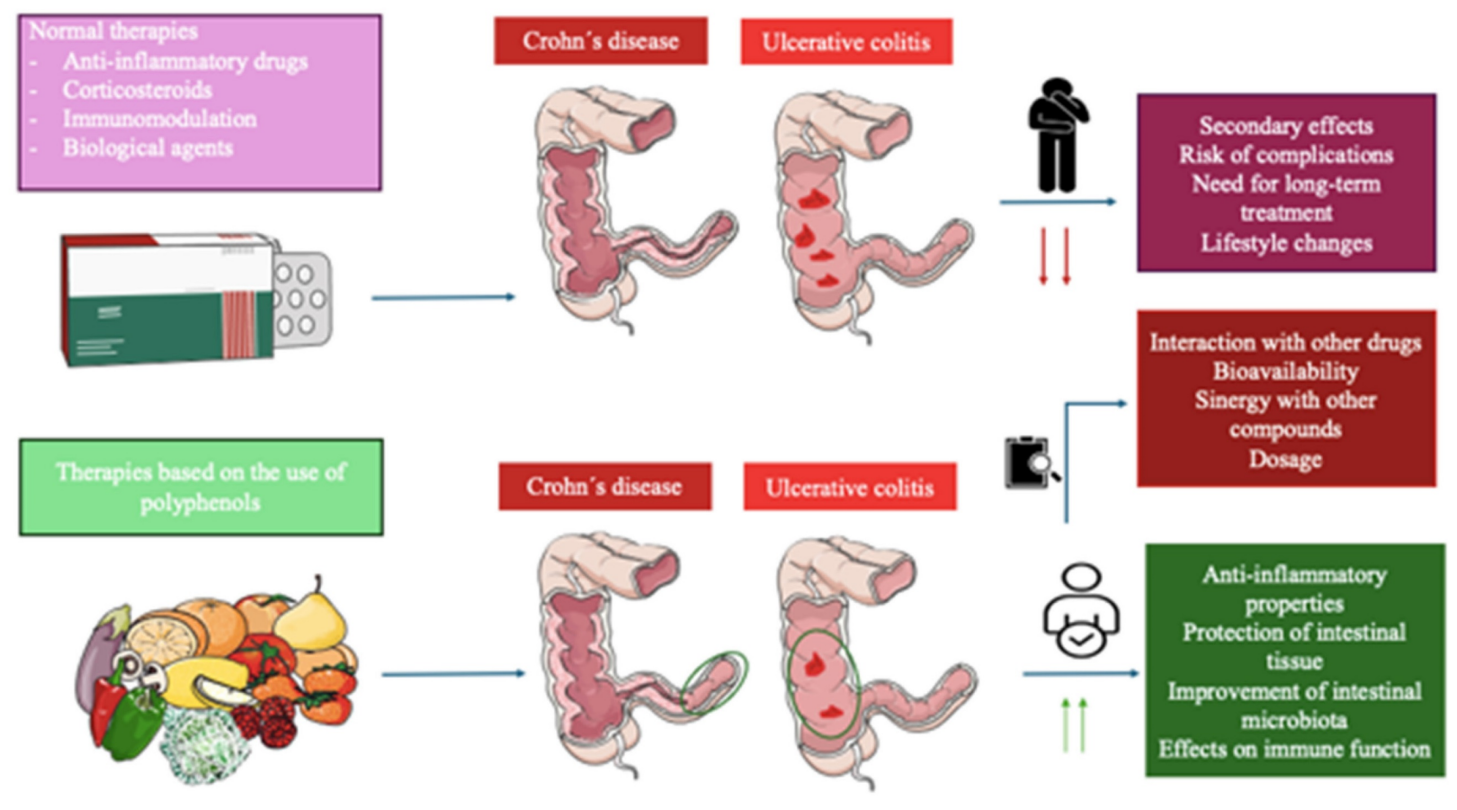
Understanding the precise role of polyphenols as part of integrative therapy in IBD. By their anti-inflammatory properties, polyphenols have been shown to reduce inflammation in patients with Chron´s disease, while also affording protection to intestinal tissues and decreasing ulceration in those with ulcerative colitis. As a result, there is a growing utilization of polyphenols, as their administration offers improved outcomes and increases comfort for patients, especially when compared to conventional therapies. However, more investigations are needed to improve the effects of polyphenols such as their interaction with other drugs, or synergies with other biological compounds, and also the dosage and bioavailability of polyphenols.

Finally, the interaction of polyphenols with different treatments should also be explored. The main pharmacological therapies for IBDs include aminosalicylates, corticosteroids, immunomodulators, antibiotics, and biologics 610. While a certain percentage of patients may benefit from several of these therapies, many others either do not respond to treatment at all or lose response over time. Furthermore, the negative side effects of conventional therapies frequently place restrictions on them. Polyphenols might interact with drug components and enzymes like cytochrome P450, which can alter drug metabolism and affect their therapeutic effects 611. Likewise, dietary polyphenols are potential substrates and/or inhibitors of Phase II enzymes like UDP-glucuronosyltransferases due to the presence of hydroxylic groups (-OH) that give them structural similarity with drugs' metabolites. Known polyphenols that inhibit drug-metabolizing enzymes include quercetin, resveratrol, chrysin, anthocyanins, naringenin, apigenin, coumarins, kaempferol, acacetin, luteolin, diosmetin, caffeic acid, and gallic acid 601. Additionally, polyphenols can affect drug transport through interactions with drug transporters 612. In parallel, certain plants and herb-based products rich in polyphenols when consumed with medications, require careful monitoring due to potential interactions. Healthcare providers need to educate patients about these risks, emphasizing that natural products are not always safe, and patients should consult pharmacists before using herbal supplements alongside medications. Effective communication about potential side effects is crucial, especially for drugs with a narrow therapeutic index like warfarin, cyclosporine A, and digoxin 601. Besides, the effects of long-term use of polyphenols remain to be better understood. In this sense, it is important to consider the possible synergy or antagonism between polyphenols and conventional medications used in the treatment of IBD. More research is needed to evaluate how polyphenols may interact with drugs such as corticosteroids, immunomodulators, and biological drugs and whether these interactions may influence the effectiveness or safety of the treatment. **Figure 4** explains the context of polyphenols and future issues to address in IBD patients.

## 4. Conclusions

Polyphenols are a class of natural compounds that have garnered significant interest due to their extensive biological effects, particularly their potent antioxidant and modulatory properties on the immune system and gut microbiota. To date, most research has demonstrated the benefits of polyphenols in preclinical models, acting through several well-reported mechanisms. Proportionally, few studies have been conducted in humans, with curcumin standing out as the most studied polyphenol, with some observational or translational studies performed on quercetin, isoflavones, resveratrol, silymarin xanthohumol, along with EVOO, flaxseed and anthocyanins from berries. Throughout the tables, important data relative to the studies conducted in humans are summarized. Also and as a limitation of our study, polyphenols are part of the bioactive compounds of different medicinal plants that could have been potentially investigated in humans not collected here. Finally, clinicians and patients must understand the precise role of polyphenols as an adjunctive therapy for IBDs. There are still many issues to be investigated before drawing clear recommendations related to their use, including concerns related to their bioavailability, dosage, formulation, administration, possible interactions with treatments, and potential adverse effects for some patients in certain contexts. To date, the most feasible recommendation would be to incorporate plant-based sources rich in polyphenols, including fruits, berries, cocoa and derivates, vegetables, legumes, nuts or spices, and to some extent coffee or tea, always included as part of a healthy dietary pattern such as MedDiet, considering the food matrix and dietary advice to maximize the bioavailability and effects of polyphenols. Further clinical trials should be conducted evaluating the use of isolated polyphenols in IBD subjects, whereas the use of curcumin, resveratrol, or biphenol-rich nutraceuticals could also be considered or tested by clinicians due to their promising benefits 613,614, although many cautions should be taken, as to date it is difficult to find standardized protocols, formulas or recommendations regarding their use. As interest in integrative approaches to healthcare continues to grow, polyphenols may emerge as valuable adjuncts to conventional treatments, offering patients additional avenues for managing their condition and enhancing their quality of life.

## Figures and Tables

**Table 1:** Flavonoids explored in the context of inflammatory bowel diseases (IBDs)

Subgroups	Compounds	Dietary/Exogenous sources	Mechanisms of action (Preclinical models)	Studies in humans (including observational and intervention studies	References
**Flavones**	**Apigenin**	Parsley, chamomile, celery, vine spinach, artichokes and oregano	Shows anti-inflammatory and antioxidant properties by inhibiting TNF-α transactivation and blocking inflammasome pathways. It alleviates intestinal injury in animal models, promotes anti-inflammatory cytokine expression, and maintains the intestinal epithelial barrier. Also, it inhibits inflammation related to carcinogenesis by repressing STA3-NF-κB signaling and regulating gut microbiota and SCFA production	-	63,65,66,333
	**Baicalein**	*Oroxylum indicum*	Reduces IBD impact by inhibiting COX-2 activity, modulating AhR/IL-22 pathways, and inactivating the NLRP3 inflammasome, among other mechanisms	-	70-75
	**Baicalin**	*Scutellaria baicalensis*	Act as an anti-inflammatory, anticarcinogenic, and immunomodulatory agent, improving intestinal health by regulating pathways such as TLR4/NF-κB, AhR/IL-22 and autophagic flux. And also, it is efficacy in human UC cells, reducing pro-inflammatory markers	-	76,78-80,82,84,88,90,92
	**Luteolin**	Carrots, parsley, broccoli, peppers, celery, olive oil, onion leaves, cabbages, apple skins, chrysanthemum flowers, peppermint, thyme, rosemary, and oregano	Inhibits pro-inflammatory mediators such as COX-2, TNF-α, and IL_6, and regulates pathways like NF-κB, JAK/STAT, and MAPKs, reducing inflammation in ulcerative colitis	-	93,95-97,100,102,103
	**Wogonoside**	*Schulletaria*	Alleviate colitis by protecting against intestinal barrier dysfunction through the reinforcement of tight junctions via the MLCK/pMLC2 signaling pathway in Caco2 cells. It also leads to the dual inhibition of NF-κB and the NLPR3 inflammasome in DSS-induced UC mice	-	105,106
	**Wogonin**	*Schulletaria*	Prevents colonic ulceration, neutrophil infiltration, oxidative stress, proinflammatory cytokines, and histological changes in DSS-induced colitis models. It promotes apoptosis by inhibiting Bcl-2 and iNOS to suppress NF-κB. Also, it regulates the Nrf2 and TLR-4/ NF-κB pathways and modulates ILC3/ILC1 plasticity	-	107,108
	**Tangeretin**	*Citrus spp pericarp*	Improves colonic tissue damage reduction and enhances gut microbiota activity in DSS-induced colitis mice. Oral administration inhibits UL-12 ND TNF-α expression through interaction with the NF-κB pathway	-	109,110
	**Nobiletin**	Citrus peels	Exhibits anti-inflammatory effects in TNBS-induced colitis by downregulating iNOS and COX-2 expression. It restores barrier functions by inhibiting the Akt/ NF-κB MLCK pathway	-	111
	**Chrysin**	Honey, propolis, and various plants, fruits, and fungi	Downregulates the PXR/ NF-κB pathway in induced colitis *in vivo*	-	112-114
**Flavonols**	**Quercetin**	Citrus, and green leafy vegetables like broccoli, flowers, and nuts	Enhances intestinal integrity, modulates microbiota, and reduces inflammation in IBD. It acts through pathways including inhibition of NF-κB, modulation of PI3K/AKT signaling, activation of AhR for TJ enhancement and suppression of NLPR3 inflammasome	Two observational studies linked higher quercetin intake to lower IBD risk and improved outcomes in UK biobank participants. An intervention study found higher patient satisfaction with quercetin containing flavonoid mixtures for hemorrhoidal disease in IBD patients. A pilot study showed potential benefits of quercetin-rich beverages on blood nutrient parameters in IBD patients, indicating the need for further research	119,120,122,124,126,128-130
	**Kaempferol**	Edible plants such as tea, broccoli, cabbage, kale, beans, endive, leek, tomato, strawberries and grapes, and herbal medical plants	It is protective against DSS-induced UC by enhancing colonic mucosal integrity and regulating gut microbiota. It reduces inflammatory markers like IL-1β, IL-6, and TNF-α, while increasing IL-10 expression through pathways involving TLR4, NF-κB, and STAT	-	131,132,134,135
	**Galangin**	Ginger, gangal, honey, and propolis	Inhibits HSP90β, elevated in UC mucosal biopsies and colitis, correlating with disease severity. It alleviates colitis by blocking HSP90β and suppressing NLRP3 inflammasome activation, reducing inflammatory markers *in vitro* and *in vivo*. Also downregulates TLR4, inhibits NF-κB p65, and promotes autophagy proteins	-	136-139
	**Myricetin**	Cranberry, dock, sweet potato leaves, chard, broad beans, and immature seeds	Reduces inflammation in acute UC by increasing IL-10 and TGFβ levels, enhancing Treg cell proportions	-	140-144,334
	**Fisetin**	Strawberries, apples, mangoes, persimmons, kiwis, grapes, tomatoes, onions, cucumbers, nuts and wine	Inhibits senescence markers and upregulates miRNAs and Akkermansia muciniphila in DSS-induced UC mice, correlating with reduced senescence and inflammation. It also exerts anti-inflammatory effects by inhibiting akt, p38, MAPK, and NF-κB signaling in colonic tissues, fomenting GSH levels, and reducing MDA	-	145-147
	**Isorhamnetin**	*Opuntia ficus-*indica, *Hippophae rhamnoides,* and *Ginkgo biloba*	It can alleviate IBD through PXR-mediated up-regulation of xenobiotic metabolism and down-regulation of NF-κB signaling. Also, it can inhibits ferroptosis	-	148,149
**Flavanones**	**Naringin**	Lemon, orange, mandarin, and grapefruit	Reduces inflammation, oxidative stress, and improves intestinal microbiota in UC models. It enhances antioxidant enzymes, decreases inflammatory cytokines, and modulates PPARγ, NF-κB, and NLPR3 inflammasome pathways. Also exhibits potential in preventing intestinal fibrosis, colorectal carcinogenesis, bone loss in IBD, and alleviating depressive behaviour in colitis models	-	152-156,158,160
	**Naringingenin**	Lemon, orange, mandarin, and grapefruit	Act as an important immunomodulator against T cell-dilated autoimmune diseases like IBDs	-	162,163
	**Hesperidin**	Lemon, sweet oranges, bitter oranges, citron, clementines, and mandarins, as well as in *Menthae piperitae, Hypericum perforatum,* and *Salvie officinalis*	Reduces neutrophil infiltration, colon damage, and inflammation by inhibiting pro-inflammatory cytokines (TNF-α, IL-6, IL-1β and IL-33, and NF-κB activation	-	128,165-168
	**Hesperetin**	Lemon, sweet oranges, bitter oranges, citron, clementines, and mandarins, as well as in *Menthae piperitae, Hypericum perforatum,* and *Salvie officinalis*	Amilorates DSS-induced colitis by maintaining an epithelial barrier through blocking the intestinal epithelial necroptosis. Also, alleviates TNBS-induced ulcerative colitis through antioxidant, anti-inflammatory properties, and by modulating JAK2/STAT3/SOCS3	-	169,170
	**Eriodictyol**	Citrus fruits, vegetables, and most medical plants	Decreases MPO expression and modulates cytokines and oxidative stress in TNBS-induced colitis in rats, inhibiting the TLR4/NF-κB pathway. It also upregulates the Hh pathway, reducing DAI, colon shortening, histological scores, and apoptosis while increasing tight junction proteins ZO-1 and occluding	-	
	**Eriocitrin**	Lemons	Significantly alleviated DSS-stimulated severe colitis in experimental animals, reducing body-weight loss, colon shortening, histopathological injury, inflammatory cell infiltration, and inflammatory cyrokine secretion	-	173,177
	**Poncirin**	Hardy oranges and mandarins. *Poncirus trifoliata*	Seems to extert antidepressant effects in mice by restoring vascular endothelial cell integrity in the hippocampus and controlling the neuroinflammatory responses of CA1 and DG regions of the hippocampus	-	178
**Isoflavones**	**Daidzein**	Soy, legumes, currants and raisins	*In vitro* studies show daidzein upregulates metallothionein gene expression, induces CAT activity, and improves tight junction integrity in Caco-2 cells. Also, attenuates LPS-induced inflammatory responses via NF-κB dependent mechanism and reduces DSS-induced UC inflammation by modulating p38, JNK, and NF-κB pathways	Observational studies suggest daidzein may reduce mucus in UC patients' feces but increase fecal pus with high intake, while isoflavone consumption may raise UC risk, especially in females. European studies linked higher daidzein intake to reduced gastrointestinal plain in UC patients. These findings highlight the importance of dosage in the impact of daidzein and polyphenols on IBD	182-186,189-191,193
	**Genistein**	Soy, legumes, currants and raisins	It has reduced DSS-induced colitis by shifting macrophages to an M2 phenotype, inhibiting the NLPR3 inflammasome, and modulating gut microbiota and inflammatory pathways. It enhances the expression of protective proteins and reduces pro-inflammatory markers.	-	194-202
	**Glycitein**	Soybeans	Is identified as a critical modulator in UC treatments like Fuzi-Lizhong Pill and Huangquin Decotion, alleviates UC by inhibiting the PI3K7Akt signaling pathway. Also, UC patients in remission reported less constipation and lower glycitein intake	-	193,209,210
	**Formononetin**	Soybeans	Bioactive compound that explains the success of UC therapy of FLP and HQT, and others.	-	212-215,217,218
	**Biochanin A**	Soybeans	Inhibits the elevation of ROS, IL-1β, IL-18, and TNF-α release, nitrite production, and the expression of iNOS and COX-2 in RAW 264.7 cells under LPS stimulation. Also promotes the restore of the intestinal barrier and promotes autophagy.	-	219,220
	**Equol**	Soybeans	A bacterial metabolite of isoflavones, has controversial roles I IBD. One study shows that equol promotes DSS-induced UC by downregulating IL-10 production, while in other study observed that equol production alleviated colitis by promoting beneficial gut microbiota	-	222,223
**Flavanols**	**Catechins (Focus on EC and EGCG)**	Epicatechin (EC), epicatheni gallate (ECG), epigallocatechin (EGC), and epigallocatechin-3-gallate (EGCG)	Catechins exter anti-inflammatory effects in IBD by modulating immune cell infiltration, oxidative stress pathways (MAPK, NF-κB , Nrf2, STAT 1/3)m and gut microbiota. EC specially reduces inflammation markers and increases antioxidant enzymes in UC models, while EGCG improves colitis symptoms by lowering pro-inflammatory cytokines and enhancing intestinal health	-	224-237
	**Proanthocyanidins (condensed tanins)**	Fruits, nuts, bark, chocolate, wine, and some plant seeds and flowers	GSPE benefits animal models of UC by reducing inflammation markers and oxidative stress while increasing antioxidant proteins like SOD and Nrf2. Also improves gut health by rebalancing microbiota, promoting beneficial bacteria, and enhancing tight junction protein expression	-	242,244-246
	**Procyanidins**	Catechin and epicatechin molecules	Show promise in treating UC by preventing M1 macrophage polarization and downregulating pro-inflammatory factors via STAT3 and NF-κB pathways. They also enhance ROS clearance, suppress MMP9 and NLPR3 inflammasome expression, and modulate gut microbiota and SCFA production	-	247-257
**Anthocyanins**	**Anthocyanidins (Cyanidin, delphidin, malvidin, peonidin, pelargodin, petudin)** **+ 1-3 sugar molecules (arabinose, galactose, glucose, rhamnose, and xylose)**	Barberry, bilberry, blueberry, cranberry, currants, grapes, mulberry black/red raspberry, grapes, strawberry, purple (sweet) potatoes, dark/purple rice and red cabbage.	Barberry anthocyanins reduce inflammation and ulcer induces UC models. Bilberry, blueberry, and cranberry anthocyanins mitigate colitis symptoms, enhance antioxidant capacity, and regulate gut microbiota, with cranberries showing superior efficacy in reducing colitis severity. Mulberry, raspberry, strawberry, and anthocyanins from purple potato, rice, and red cabbage also demonstrate significant anti-inflammatory and gut microbiota modulation effects, suggesting their potential in managing IBDs	In a pilot study, 63.4% of UC patients achieved remission and 90.9% showed a response to a daily anthocyanin-rich bilberry preparation, with decreased total Mayo scores and fecal calprotectin levels during the treatment phase. Molecular analysis revealed reduced pro-inflammatory cytokines and increased immunoregulatory cytokines in responsive patients, with no serious adverse effects reported. However, calprotectin levels and DAI increased after stopping bilberry intake	261-300
**Chalcones**	**Xanthohumol**	*Humulus lupulus L.*	Demonstrates anti-inflammatory and anti-fibrotic effects in colitis by inhibiting pathways such as TLR4/MD-2, NF-κB, and TGF-β/Smad, ad reducing oxidative stress and pro-inflammatory cytokines. Studies show XN alleviates colitis symptoms, prevents colonic lesions, and modulates gut microbiota, increasing anti-inflammatory microorganism	No studies have specially evaluated xanthohumol (XN) in humans with IBD, but a phase II clinical trial is testing its safety and tolerability in adults with active Chron´s disease. A pilot study with a micronutrient and phytonutrient-rich formula, including XN, showed significant increases in serum folate and changes in leukocyte subtypes in IBD patients. Although preliminary evidence is promising, further studies are needed to confirm XN´s potential in IBD treatment	101,301-307,309,310
	**Isoliquiritigenin**	*Glycyrrhiza uralensis, Mongolian glycyrrhiza,* and *Glycyrrhiza glabra*	Exhibits anti-inflammatory and antioxidants effects by inhibiting NF-κB, NLRP3, and MAPK pathways, and activating Nrf2. In DSS-induced UC mice, ISL improved symptoms and colon integrity by suppressing ERK1/2 and p38 phosphorylation, and reducing inflammation markers. Preclinical studies suggest ISL and licorice extract as potential IBD treatments	-	311-316
	**Cardamonin**	Various plants	Exhibits anti-inflammatory properties by reducing NO production and downregulating iNOS, TNF-α, and IL-6 expression, while inhibiting NF-κB signaling. In DSS-induced colitis mice, it mitigates symptoms, reduces inflammatory markers, and blocks NF-κB and MAPK pathways	-	317-322
	**Phloretin**	Apples, pears, and strawberries	Acts as a potent antioxidant and anti-inflammatory agent by modulating signaling pathways and maintaining epithelial integrity. It reduces inflammation markers, oxidative stress, and mitochondrial membrane depolarization *in vitro* and in a rat and mouse colitis models, while also preserving gut microbiota balance	-	323-327
	**Liochalcone A**	*Glycyrrhiza inflata*	Significantly protected mice against DSS-induced colitis, reducing weight loss, disease activity index, histological damage, and inflammation while preserving intestinal barrier integrity and modulating gut microbiota	-	328,329
	**Butein**	*Toxicodendron verniciflumm*	Significantly ameliorated colitis in Il-10 (-/-) mice by reducing the colonic inflammatory score by over 50%. It lowered the expression of Il-6, IL-1β, IFN-γ, pSTAT3, and MMP-9, and inhibiting IL-6 induced STAT3 activation in colon 205 cells	-	330
	**Flavokawain B**	Zingiberaceae and Kawa family	Demonstrated significant therapeutic effects in a DSS-induced IBD mouse models by reducing weight loss, restoring colon length, and mitigating inflammation. It targeted TLR2 to inhibit the TLR2-MyD88 complex formation, suppressing the NF-κB signaling pathway *in vivo* and *in vitro*	-	331
	**Artipilin C**	Brazilian green apples propolis	It ameliorates UC and colts-associated colorectal cancer by targeting p21-activated kinase 1 (PAK1).	-	332

**Table 2:** Phenolic acids explored in the context of inflammatory bowel diseases (IBDs)

Subgroups	Compounds	Dietary/Exogenous sources	Mechanisms of action (Preclinical models)	Studies in humans (including observational and intervention studies	References
Hydroxybenzoic	**Gallic acid**	Grapes, blackberries, strawberries, and raspberries	Downregulates the NLPR3 inflammasome and improves colitis in DSS-induced mice by reducing ammonia levels and gut microbiota dysbiosis, increasing beneficial bacteria like *Lactobacillaceae* and *Prevotellacea.* It modulates metabolic pathways, enhancing carbohydrate and bile acid metabolism while decreasing amino acid metabolism, and reduces pro-inflammatory cytokines while increasing anti-inflammatory cytokines	-	341-347
	**Ellagic acid**	Fruits, nuts, and seeds, such as pomegranates, raspberries, strawberries, walnuts, and almonds	Inhibits NF-κB and ERK1/2 in Caco-2 cells, reducing inflammation, oxidative stress, and intestinal permeability. Dietary EA supplementation bolsters Nrf2 pathways, protecting against oxidative stress and maintaining gut barrier integrity and morphology in piglets and TNBS-induced colitis mice. Also, reduces inflammation and histological scores in the acute and chronic DSS-induced UC models by downregulating COX-2, iNOS, and blocking p38, MAPK, NF-κB, and STAT3	-	348-353
	**Protocatechuic acid**	Olives, roselle, du-zhong, calamondin, and white wine grapes	One study demonstrated that PCA reduced disease activity, inflammation, and histological damage in UC mice by modulating Bacteroidetes and downregulating ferroptosis, with similar effects in Erastin-treated Caco-2 cells. Also, it reduced IL-1β, IL-6, and TNF-α levels, and improved tight junction protein distribution in DSS-induced colitis	-	356-360
	**Vanillic acid**	Herbs, rice, maize, and edible plants and fruits	It is demonstrated that VA treats DSS-induced mice colitis by inhibiting ferroptosis and restoring intestinal epithelium homeostasis through a CAIX-INSIG2-STIM1-SCAP-SREBP1-SCD1 pathway, preserving barrier integrity and reducing inflammation. In another study, it is found that VA alleviated colitis symptoms, reduced COX-2 expression, NF-κB p65 activation, and plasma IL-6 levels	-	361,362
Hydroxycinnamic	**Caffeic acid**	Plant-based foods, including fruits and vegetables, and drinks like tea or wine	Act as an anti-inflammatory modulator by suppressing NO. IL-1β, IL-6, IL-8, and TNF-α in RAAW 264.7 and HT-29 cells. It attenuates DSS-induced murine UC by interfering with macrophage activation and increasing Akkermansia populations	-	364-371
	**Chlorogenic acid**	Apples, artichoke, betel, burdock, carrots, eggplants, grapes, kiwi, potatoes, tea or tomatoes, among others	Alleviates DSS-induced colonic damage by reducing inflammation, oxidative stress, and apoptosis while modulating ERK1/2. P38, and JNK pathways. It downregulates miR-155 and inactivates the NF-κB/NLRP3 pathway in macrophages, offering protective effects against colitis	-	373-379
	**Ferulic acid**	Orange, apples, and tomatoes	It demonstrates effects on intestinal tight junctions, suppressing ER stress, NO generation, and inflammation *in vitro. In vivo,* it alleviates colitis in various animal models by inhibiting inflammatory pathways, and reducing cytokine levels	-	380-385
	**Sinapic acid**	Berries, citric fruits, oil seeds, wheat, rice, spices, and plants like *Salvia officinalis* and *Myristica fragans*	It mitigates intestinal permeability, reduces inflammatory cytokine expression, and restores tight junction protein localization both *in vitro* and *in vivo.* It inhibits the NF-κB and MAPK/ATF-2 pathways by binding to TAK1, improves gut microbiota imbalance, and reduces IBD symptoms, showing potential as a nutraceutical and pharmaceutical agent for IBD treatments	-	386-392
	**Rosmarinic acid**	Rosemary, basil, and mint, among others	Demonstrates significant antioxidant, anti-inflammatory, and antimicrobial properties, proving effective in alleviating intestinal inflammation, ut dysbiosis, and tight junction damage in IBD	-	393-397
	**Coumaric acid**	Apples, pears, grapes, oranges, tomatoes, berries, beans, potatoes, onions, maize, oats, wheat, mushrooms and medicinal herbs	-	-	399,401
	**Quinic acid**	Yerba mate, white, green teas, coffee, microalgae, and cyanobacteria	One study demonstrated the therapeutic potential for UC by significantly increasing HO-1, Nrf2, and NQO1 mRNA expression while reducing TNF-α and IL-1β protein levels in colon tissue. Also attenuates UC by inhibiting the TLR4-NF-κB and NF-κB-iNOS-No signaling pathway, reducing oxidative stress, inflammation, apoptosis, and histopathological damage	-	400,402

**Table 3:** Stilbenes explored in the context of inflammatory bowel diseases (IBDs)

Subgroups	Compounds	Dietary/Exogenous sources	Mechanisms of action (Preclinical models)	Studies in humans (including observational and intervention studies	References
Stilbenes	**Resveratrol**	Wine and grapes	Is a potent antioxidant and anti-inflammatory molecule explored for managing IBD. It modulates responses and signaling pathways, but its poor water solubility and low bioavailability limit clinical applications. It influences gut microbiota, reducing inflammation, enhancing tight junction protein expression, and modulating several pathways	Human studies show that resveratrol supplementation benefits people with UC. In a trial with the Mediterranean diet (MD) plus resveratrol or curcumin, all groups showed reduced disease activity and inflammation, and improved quality of life. Another study with 500 mg/day resveratrol for 6 weeks reported significant reductions in inflammatory markers and improved quality of life compared to placebo. Overall, 500 mg resveratrol supplementation enhances life quality and reduces disease activity in UC patients, but long-term effects need further study	405-415,417-429,431,432,434,435
	**Piceatannol**	Grapes, peanuts, and blueberries	Is similar to resveratrol, has shown promise in reducing inflammation and modulating immune response. It decreases proinflammatory mediators, inhibits cell apoptosis, and improves microbiota composition, particularly beneficial probiotics. Oral administration of PIC attenuates DSS-induced colonic inflammation, decreases inflammatory mediators, and improves colonic architecture	-	436-439
	**Pterostilbene**	Blueberries and *Pterocarpus marsupium* heartwood	Exhibits anti-inflammatory effects in DSS-induced models. It reduces inflammation, aberrant crypt foci, and maintains mucin2 and E-cadherin expression, while also inhibiting Th1 and Th17 cell proliferation and promoting Treg differentiation. Also decreases, TNF-α expression and alleviates colitis symptoms	-	440-444

**Table 4:** Lignans explored in the context of inflammatory bowel diseases (IBDs)

Subgroups	Compounds	Dietary/Exogenous sources	Mechanisms of action (Preclinical models)	Studies in humans (including observational and intervention studies	References
**Lignans**	**Flaxseed and Secoisolariciresinol diglucoside**	Flaxseed and flaxseed oil	Reduces mortality and colonic ulcers, while aqueous-methanolic crude extracts (Fs.Cr) enhance mucin content and exhibit anti-inflammatory, antispasmodic, and antibacterial effecs. Phenolic compounds from flaxseed and SGD also reduce inflammation and improve mucosal repair in colitis models	Human studies on flaxseed oil found that flaxseed supplementation benefits UC patients. In a 12-week trial with 75 UC patients, both 30 g/day of grounded flaxseed (GF) and 10 g/day of flaxseed oil (FO) significantly reduced inflammatory markers, disease severity, blood pressure, and waist circumference compared to placebo. Additional trials showed improvements in metabolic syndrome parameters, inflammatory markers, and quality of life. However, long-term studies are needed for definitive conclusions	449-461
	**Sesame seeds, sesamin, and sesamol**	Sesame seeds, sesamin, and sesamol	Have shown notables benefits in treating IBD in preclinical models. Sesame oil and sesame cake reduce inflammation, fibrosis, and oxidative stress, while sesamin and sesamol specially mitigate UC symptoms by modulating inflammatory and oxidative pathways, enhancing gut barrier function, and improving both physical and mental health in affected animals	-	462-472
	**Schisandrin**	*Schisandra chinesis*	Show promise in treating UC. SCH treatments alleviate colitis severity, improve gut microbiota, and modulate inflammatory pathways, including SGK1/NLRP3 and NLRP3 inflammasome. Specifically, Schinsandrin B reduces pro-inflammatory cytokines and enhances epithelial barrier protection, while Schisandrin c improves intestinal permeability and Deoxyschinsandrin offers cytoprotection against oxidative stress-induced cell apoptosis	-	474-480
	**Magnolol and Honokiol**	*Magnolia officinalis*	Magnolol reduces pro-inflammatory cytokines and improves colitis symptoms by modulating MAPK and NF-κB pathways, while honokiol enhances epithelial barrier integrity, reduces cytokines, and inhibits NLRP3 inflammasome activation	-	482-492
	**Magnolin**	*Magnolia genus*	Animal studies showed that MGL treatment alleviates weight loss, colon shortening, disease activity, and inflammation in colitis models. MGL also enhances intestinal barrier functions by preserving tight junction proteins and preventing epithelial cells apoptosis	-	491-493
	**Arctigenin**	*Arctium lappa*	Alleviates colitis by reducing body weight loss, disease activity index, and colon histological damage. It promotes intestinal epithelial cell recovery, decreases immune cell infiltration and suppresses inflammation and oxidative stress by inhibiting MAPK and NF-κB pathways	-	494-496
	**Enterolactone and enterodiol**	Resulting from the metabolism of other lignans by the gut microbiota	Mitigates inflammation-induced loss of intestinal epithelial barrier integrity and oxidative stress *in vitro*	-	497,498
	**Koreanaside A**	*Forsythia koreana*	Alleviates inflammatory response by downregulating AP-1, NF-κB, and JAK/STAT signaling in LPS-induced macrophages and DSS-induced colitis mice	-	499
	**Fargesin**	*Flos magnoliae*	Anti-inflammatory effects on chemically induced IBD through NF-κB, signaling suppression	-	499

**Table 5:** Curcuminoids, olive-associated polyphenols (including secoiridoids and 5-HT), coumarins, and flavonolignans

Subgroups	Compounds	Dietary/Exogenous sources	Mechanisms of action (Preclinical models)	Studies in humans (including observational and intervention studies	References
Curcuminoids	Curcumin	Turmeric	Is a potent anti-inflammatory and antioxidant with broad therapeutic potential, including anti-tumor, anti-apoptotic, and immunomodulatory effects. It modulates multiple inflammatory pathways, including NF-κB, COX, and various cytokines, and influences iron metabolism and autophagy in IBDs	Human studies show curcumin´s promise managing IBD, particularly UC. Clinical trials report significant improvements in disease activity, inflammatory markers, and quality of life with curcumin supplementation. However, some studies yielded mixed results, underscoring the need for standardized dosages, formulations, and further large-scale trials to validate curcumin´s efficacy as an adjunct therapy in IBd management	501-528
Secoiridoids and polyphenols from olives/EVOO	Extra virgin olive oil	Olives/EVOO	Preclinical studies demonstrated the potential of EVOO in managing IBD, with findings showing that EVOO can reduce inflammation, oxidative stress, and cell damage in various models. It has been found to modulates cytokines, COX-2, and iNOS while influencing signaling pathways like MAPk and PPARγ	Clinical trials on EVOO and its poluphenols for IBd are limited. Studies have shown that EVOO can reduce inflammation markers and gastrointestinal symptoms in Uc patients and that phenolic-rich EVOO can enhance cardioprotective benefits and gut microbiota. Also, Mediterranean diet, including EVOO, has demonstrated over all benefits for IBD management, such as improved BMI, reduced inflammation, and better disease management compared to other diets	533-535,537-545
	Hydroxytyrosol		Has shown promising results in preclinical IBD models. Studies demonstrate that HT reduces inflammation, oxidative stress, and apoptosis while enhancing antioxidant defenses and gut microbiota composition	-	546-551
	Oleuropein		Studies reveal that Ole reduces oxidative stress, inflammation, and apoptosis, and improves disease activity in UC models. It decreases pro-inflammatory cytokines and COX-2 expression while promoting healing and enhancing antioxidant defenses	-	552-554
Coumarins	Esculetin and 4- methylesculetin	Cinnamon; Vegetables, spices, fruits, and medicinal plants including all parts of the plants-fruits, roots, stems and leaves	Esculetin at 5 mg/kg in a rat TNBS-induced IBD model, exhibited strong anti-inflammatory effects, reducing MDA levels, preventing GSH depletion, and decreasing pro-inflammatory cytokines. It showed efficacy comparable to prednisolone and was 10 tomes more effective than sulfasalazine. Similarly, 4-methyl-esculetin demonstrated superior efficacy in reducing colitis symptoms, preventing GSH depletion, and inhibiting inflammation, with effects comparable to established treatments	-	557-562
	Aesculin		Showing minimal cytotoxicity, significantly alleviated DSS-induced colitis symptoms by reducing inflammatory factors like iNOS, IL-1β, and TNF-α. It also attenuated NF-κB signaling and enhanced PPARγ nuclear localization	-	563
	Daphnetin		He *et al.* found that daphnetin alleviated colitis severity in mice by improving intestinal structure, increasing ZO-1, occludin, and BCL-2 levels, and reducing Bax and cleaved caspase 3 expression. It also suppressed MAD, SOD activities, inflammatory cytokines, and apoptosis, while inhibiting JAK/STAT signaling via REG3A activation	-	565
	Fraxetin		Demonstrated notable efficacy in mitigating DSS-induced colitis symptoms, including body weight loss, colon shortening, tissue damage, and disease activity index. It also inhibits NF-κB pathway, suppression of NLRP3 inflammasome, restoration of Gut barrier function, and modulation of gut microbiota	-	566
	Umbelliferone		Improves macroscopic and histological damage in an acetic acid-induced UC rat model. It reduced inflammatory markers, downregulated TLR4, NF-κB, and iNOS, and upregulated SIRT1 and PPARγ signaling pathways	-	567
	Paepalantine		Alleviates TNBS-induced colonic damage in mice at 5 and 10 mg/kg. it improved mucosal recovery and reduced oxidative stress by preventing glutathione depletion and inhibiting nitric oxide activity	-	569
	Coumarin		Reduces colonic damage in TNBS-induced colitis models. Their protective effect was linked to preventing glutathione depletion caused by inflammation	-	570
Flavonolignanes	Silymarin	Milk thistle *(Sylybum marianum)*	Is a potent anti-inflammatory that inhibits NF-κB pathways and enhances antioxidant defenses via Nrf2 activation. It shows potential as a tool against viral-associated IBD due to its properties and effectiveness in reducing colitis symptoms	In a randomized, double-blinded trial with 80 UC patients, silibin supplementation improved hemoglobin levels, erythrocyte sedimentation rate, and disease activity index, helping maintain remission better than placebo	573,575-578,581
	Silibin	*Sylybum marianum*	Has ant-inflammatory and chemopreventine benefits I preclinical IBD models. In a TNBS-induced colitis rat model, silybin reduced NF-κB activity and inflammatory markers, enhancing antioxidant defenses	-	579,580
